# High prevalence of *Plasmodium ovalewallikeri* in co-infections with *Plasmodium falciparum* in symptomatic than asymptomatic *Plasmodium parasite* infections in Kisumu, Kenya

**DOI:** 10.1371/journal.pone.0334188

**Published:** 2026-10-07

**Authors:** Jackline A. Juma, Oliver J. Watson, Dennis W. Juma, Benjamin H. Opot, Gladys Chemwor, Mary Mutahi, Raphael Okoth, Edwin W. Mwakio, Redemptah Yeda, Charles O. Okello, Agnes Cheruiyot, Duke Omariba, Liliana Dayo, Hellen A. Ogola, Gladys Biwott, Charles Adega, Fredrick Oluoch, Vincent Omondi, George Otieno, Edwin Kamau, Ben Andagalu, Peter Sifuna, Amanda Roth, Sammy Khagayi, Eric C. Garges, Bernhards Ogutu, Hoseah M. Akala

**Affiliations:** 1 Department of Emerging and Infectious Diseases, Walter Reed Army Institute of Research-Africa/Centre for Clinical Research, Kenya Medical Research Institute, Kisumu, Kenya; 2 Department of Infectious Disease Epidemiology, Medical Research Council, Centre for Global Infectious Disease Analysis, Imperial College London, London, England; 3 The Kenya Medical Research Institute (KEMRI) / Wellcome Trust Research Programme, Centre for Geographic Medicine Research-Coast, Kilifi, Kenya; 4 Department of Health County Government of Kisumu, Ministry of Health, Nairobi, Kenya; 5 Department of Pathology and Laboratory Medicine, Tripler Amy Medical Center, Honolulu, Hawaii, United States of America; 6 Medical Communications for Combat Casualty Care, Fort Detrick, Maryland, United States of America; 7 Centre for Global Health Research, Kenya Medical Research Institute, Kisumu, Kenya; 8 Walter Reed Army Institute of Research-Africa, Nairobi, Kenya; 9 Centre for Clinical Research, Kenya Medical Research Institute, Nairobi, Kenya; The University of New Mexico, UNITED STATES OF AMERICA

## Abstract

*Plasmodium falciparum* (*Pf*), *Plasmodium ovalewallikeri* (*Pow*), *Plasmodium ovalecurtisi* (*Poc*), and *Plasmodium malariae* (*Pm*) coexists in Kenya sympatrically. Some infections progress to classical symptoms of malaria prompting hospital visits, while others remain asymptomatic. There is limited information on whether the species composition status of infections in endemic areas influences progression to either asymptomatic or symptomatic infection. This cross-sectional study determined the composition of *Plasmodium* species among symptomatic versus asymptomatic participants in Kombewa, Kenya. Between 2015 and 2016, samples from 741 individuals, comprising of 437 from symptomatic participants at Kombewa Subcounty hospital and 304 from asymptomatic participants from within Kombewa Subcounty hospital’s catchment area were analyzed for *Plasmodium* species composition using real-time qPCR assay targeting the small subunit ribosomal RNA (ssrRNA) gene. R Studio was used to describe *Plasmodium* species prevalence, and the odds of occurring within a given infection presentation. Co-infection analysis revealed 741 samples predominantly contained *Pf* 655/741 (88.3%) followed by *Pow* 153/741 (20.6%), *Pm* 70/741 (9.4%) and *Poc* 64/741 (8.6%); 12/741 (1.62%) samples did not amplify any species. A total of 535/741 (72.2%) samples contained single species, and 194/741 (26.18%) samples contained multiple species. Species distribution differed significantly, with *Pf* and *Pow* more prevalent in symptomatic cases, while *Pm* and *Poc* were more common in asymptomatic individuals (p = 0.000). *Pm* and *Poc* single-species infections occurred among asymptomatic infections only. There were significantly higher odds of multiple species occurrence for infections containing *Pf*/*Pow* 92 (95.8%) among symptomatic than asymptomatic infections (OR 1.5, 95% CI [1.36–1.65], p = 0.000). Overall, mixed-species infections involving *Pf* and *Pow* were significantly more common in symptomatic individuals, posing a risk of false diagnosis by *Pf*-Histidine-Rich Protein 2 test. This underscores the need for deployment of interventions that target all *Plasmodium* species. These findings on the burden across symptomatic vs asymptomatic infections suggest a need for determination of the role of species composition in disease progression.

## Introduction

Human malaria infection is attributed to six *Plasmodium* species namely, *P. falciparum, P. ovalecurtisi, P. ovalewallikeri, P. malariae, P.vivax* and *P. knowlesi* [1–4]. *P. ovalewallikeri* and *P. ovalecurtisi* are now considered two morphologically identical but distinct species and the names *P. ovalewallikeri* and *P. ovalecurtisi* are preferred over the classical trinominal names. The burden of these species in natural infections varies globally [5, 6], along with their key vectors [7].

*P. falciparum* is responsible for the most severe form of disease and is the most prevalent in Sub-Saharan Africa [8] where it causes the highest number of fatalities globally [8]. *Plasmodium vivax* is the second most dominant species that causes recurring malaria, with the highest burden in Asia and South America, and in Africa it is mainly in Ethiopia and Sudan [9]. The less prevalent species, *P. ovalewallikeri* and *P. ovalecurtisi,* occur primarily in Africa [10, 11]. *P. malariae* is predominantly found in South America, Asia, and Africa, and occurs mainly as mixed infections with *P. falciparum* at a lower frequency [12]. Consequently, the burden and severity of disease caused by non-falciparum species, which are generally presumed to be milder than those associated with *P. falciparum*, remain poorly characterized in endemic human populations [13–15].

The World Health Organization recommends that all suspected malaria cases receive an accurate parasitological test to confirm infection before antimalarial treatment is administered [16–22]. However, the parasitological diagnosis methods [23, 24], are less accurate in the diagnosis of low parasitemia [25] and distinguishing infecting species [26] in asymptomatic individuals [27] and therefore likely to underestimate the burden and complexity of infection in the population [28, 29]. These methods, therefore, have limited utility in pre-elimination settings. The innovation of molecular diagnostic methods capable of detecting subclinical and submicroscopic *Plasmodium* infections [30, 31], which serve as reservoirs for ongoing transmission [32], is emerging as an important surveillance tool for malaria elimination strategies [33–36].

In Kenya, *P. falciparum* infection occurs predominantly as a mono-infection or sympatrically with *P. malariae* and *P. ovalewallikeri*, and *P. ovalecurtisi*. This co-endemicity of species leads to the occurrence of blood-stage mixed *Plasmodium* species infections [37], which are mostly acquired from separate inoculations when a recently acquired infection of one species might supervene a chronic infection with another [38] or simultaneous inoculation of the sporozoites of two or more species from a doubly infected *Anopheles* mosquito [38–40]. Studies have highlighted variations in the clinical presentation of disease per species relative to the unique parasite biology of each malaria species. Evaluating the species composition of infections among symptomatic individuals seeking care at health facilities and asymptomatic individuals within households is essential for understanding and addressing the rising prevalence of non-*Plasmodium falciparum* species in the context of improved access to accurate diagnosis and timely treatment, which are key for transitioning regions with declining malaria to pre-elimination.

Clinical symptoms of malaria typically develop between 7 and 85 days after initial infection, depending on the infecting *Plasmodium* species [41–45]. Parasitemia may also persist in susceptible individuals for up to eight years before the onset of symptoms [44–47]. Initial symptoms of malaria may include a high temperature of 38°C and above, muscle pains, and generally feeling unwell, often characterized by recurrent fever and chills [44], which often trigger a treatment-seeking response [22] once parasite replication becomes synchronous. In some cases, the infection may generate malarial parasitemia in the absence of fever or other classical symptoms and signs, in otherwise healthy individuals, therefore regarded as asymptomatic *Plasmodium* parasite carriers [23, 48, 49]. Asymptomatic *Plasmodium* infections account for up to 60% of infections [50–53] in some regions. It has been suggested that infections remain asymptomatic due to sustained submicroscopic peripheral parasitemia [54, 55], older age, and therefore partial immunity of the infected individual [56, 57], and varying malaria pyrogenic threshold [58, 59]. In a 2011 study, the complex interactions between infecting *Plasmodium* species [58, 60], were shown to affect the disease outcome such as the occurrence of symptoms [61] and transmissibility, bringing in to focus the role of these interactions in either limiting or promoting disease progression.

In a region with natural infections comprising more than one *Plasmodium* species [62, 63], there is uncertainty about whether infection with multiple species reduces or promotes the incidence of symptoms in the infected individual as a means of maintaining transmissibility. Our previous study showed the prevalence of *Plasmodium* species co-infections changes over time, among symptomatic individuals seeking for treatment at selected hospitals across Kenya [64], but did not describe the burden among asymptomatic infections. Given the varying disease progression among *Plasmodium* species such as longer period of case management for relapsing species, estimating of the burden of species in the population is useful in informing choice of intervention needed to lower malaria in line with the WHO goal that targets 90% reduction of malaria by 2030. This study aimed to estimate the composition of *Plasmodium* parasite species among symptomatic versus asymptomatic infections in Kisumu County in western Kenya.

## Materials and methods

### Ethical considerations

Ethical approval was obtained from Kenya Medical Research Institute (KEMRI), Scientific and Ethics Review Unit (SERU), and Walter Reed Army Institute of Research (WRAIR) institutional review boards (protocol numbers: KEMRI 1330/WRAIR 1384 and KEMRI 2721/ WRAIR 2064. A written informed consent /assent was obtained from each of the study participants. The confidentiality of information obtained from study participants was assured by using unique identifiers.

### Study site and sample collection

Kombewa Health and Demographic Surveillance System (HDSS) grew out of the Kombewa Clinical Research Centre in 2007 and has since established itself as a platform for conducting nested studies and local disease surveillance, according to a study by Sifuna and co-workers. This study was conducted within Kombewa HDSS, which forms the main catchment of the malaria cases for the Kombewa Sub-County hospital, an area that is categorized as a rural Lake Endemic Zone with perennial and intense malaria transmission as per the National Malaria Control Programme 2016 (NMCP).

This sub-study analysed *Plasmodium-*positive samples obtained from two studies that were conducted concurrently between 2015 and 2016. The first study was entitled “Epidemiology of malaria and drug sensitivity patterns in Kenya”, which commenced from 1^st^ September 2015–1^st^ June 2016. The second study was entitled, “Baseline Epidemiology of Malaria Gametocyte Carriage and Transmission Dynamics in a Holoendemic Transmission Setting in Kisumu County, Western Kenya”, which commenced on 18^th^ March 2015–1^st^ February 2016.

The first study enrolled symptomatic participants aged six months and above presenting at Kombewa Sub-County Hospital with symptoms consistent with uncomplicated malaria, characterized by symptomatic, laboratory-confirmed *Plasmodium* infection in a patient who had no clinical or laboratory evidence of severe malaria or vital organ dysfunction and able to tolerate oral antimalarial therapy. These individuals presented with clinical features including fever (≥37.5 °C) or a history of fever within the preceding 48 hours accompanied by symptoms such as Chills, rigors, sweating, Headache, Malaise, fatigue Myalgia and arthralgia, Nausea and/or vomiting [65] Participants who were clinically stable, consistent with uncomplicated malaria, and who tested positive for malaria by rapid diagnostic test (RDT) and/or microscopy were eligible for inclusion. Under this study, these individuals were referred to as symptomatic malaria participants. These individuals were enrolled into the study upon providing written informed consent/assent. Each participant provided 2–3 ml of whole blood for malaria parasite species genotyping alongside other endpoints of the main study reported elsewhere [66, 67]. All *Plasmodium Plasmodium* positive cases were treated with artemether-lumefantrine (AL) (Coartem®), as per the Kenya Ministry of Health’s recommended case management guidelines for uncomplicated malaria. The healthcare workers administered and observed the taking of the first dose of AL 20 mg/120 mg tablets.

The second study enrolled asymptomatic participants and sought to estimate the level of gametocyte carriage in the study population by PCR. These comprised individuals of all ages, without clinical features suggestive of malaria, such as fever (≥37.5 °C) or history of fever within the previous 48 hours, and absence of symptoms such as chills, headache, malaise, or sweating. Under this study, these individuals were referred to as asymptomatic malaria participants. Members of selected households within Kisumu West sub-County, which is the main catchment for the Kombewa sub-County hospital (Fig 1 **Map of Kombewa**), willing to be tested for the presence of malaria parasites were enrolled into the study upon providing written informed consent/assent. During this household visit, vital signs were measured to rule out symptoms of malaria. Based on these readouts, each of the otherwise healthy participants provided 4 ml of whole blood for the diagnosis of malaria. Each sample was tested within 24 hours, and all participants found to have *Plasmodium* genus infection were treated with the attending healthcare worker administering the first dose of AL 20 mg/12 mg tablets.

**Fig 1:**
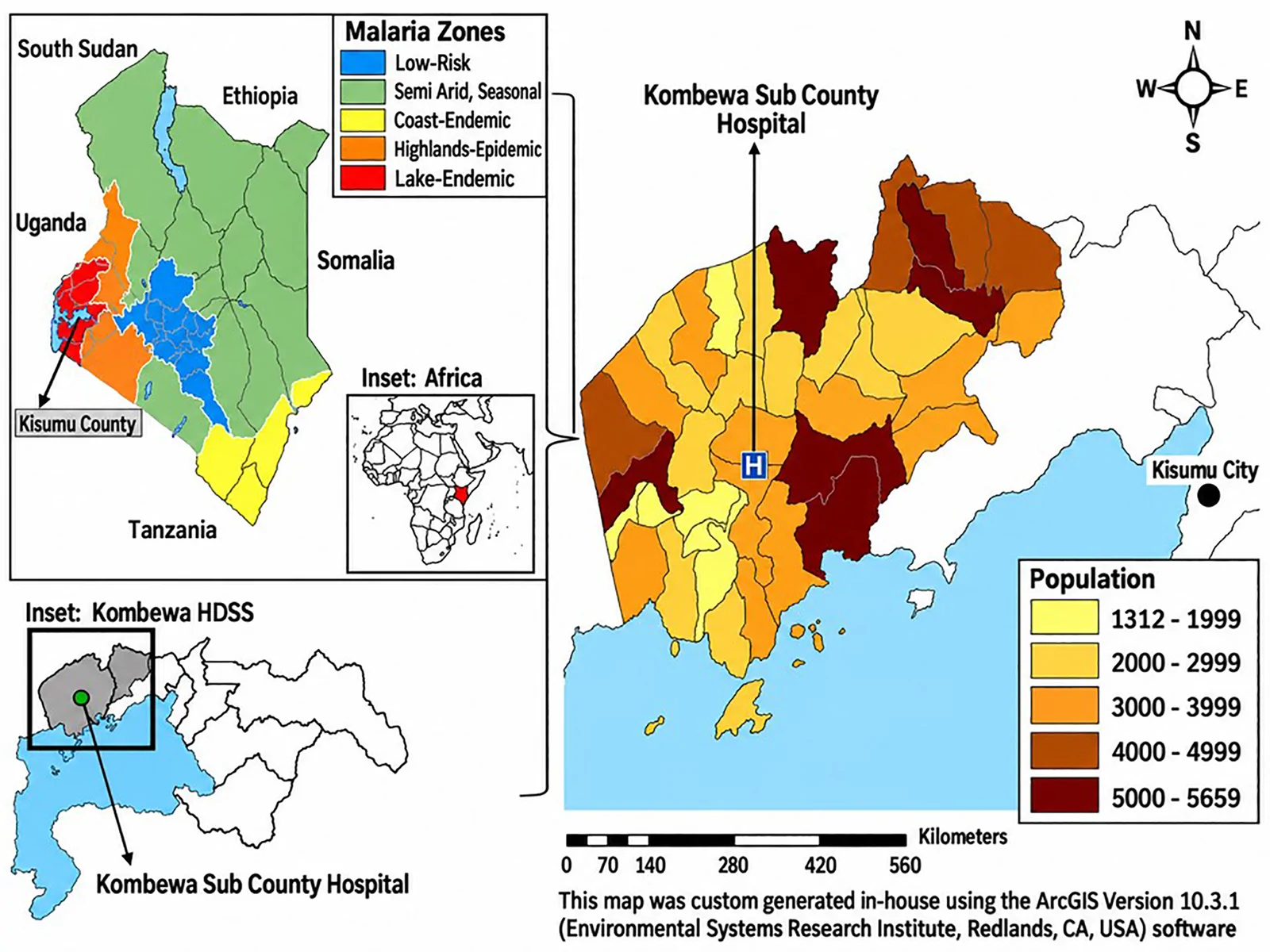
Map of Kombewa. This shows Kombewa region where Kombewa sub-County hospital is located. Map data from OpenStreetMap (https://www.openstreetmap.org/copyright). The base maps used in all three images in Figure 1 were obtained from Open AFRICA. *This map was custom generated in-house using the ArcGIS Version 10.3.1 (Environmental Systems Research Institute, Red-lands, CA, USA) software.

### Molecular assay

#### Nucleic acids extraction.

The parasite nucleic acids were extracted from blood samples obtained from individuals enrolled in the two studies using a commercial extraction kit (QIAamp DNA Blood Mini Kit, Qiagen) as recommended by the manufacturer’s instructions.

#### *Plasmodium* genus diagnosis by molecular methods.

The extracted nucleic acids were analysed for malaria diagnosis using *Plasmodium* genus-specific qPCR assay as previously described [68, 69]. PCR amplified the P*lasmodium* genus 18S ribosomal ribonucleic acid (rRNA) gene using primers listed in Table 1. The RN*ase* P gene, which is a human housekeeping gene, was used as an extraction and qPCR assay control.

**Table 1:** Primers and probes used for screening and identification of *Plasmodium* species.

Primers Probes	Sequence 5’-3’	Target genus/species
PLU F	GCTCTTTCTTGATTTCTTGGATG	*Plasmodium* spp.
PLU R	AGCAGGTTAAGATCTCGTTCG	*Plasmodium* spp.
PLU P	ATGGCCGTTTTTAGTTCGTG	*Plasmodium* spp.
RNaSP F	TGTTTGCAGATTTGGACCTGC	Human RNase p
RNaSeP R	AATAGCCAAGGTGGAGCGGCT	Human RNase p
RNaseP P	TGCGCGGACTTGTGGA	Human RNase p
FAL F	ATTGCTTTTGAGAGGTTTTGTTACTT	*P. falciparum*
FAL R	GCTGAGTATTCAAACACAATGAACTCAA	*P. falciparum*
FAL P	CATAACAGACGGGTAGTCAT	*P. falciparum*
MAL F	GCATGGAATTTTGTTACTTTGA	*P. malariae*
MAL R	ATGCCTGTAGTATTCAACACAGAAAC	*P. malariae*
MAL P	TGTTCAAAGCAAACAGTTAAAACA	*P. malariae*
OVA F	TTTTGAAGAATACATTAGGATACAATTAATG	*P. ovale curtisi*
OVA R	CATGCTTCCTCTAAGAAGCTTTACAAT	*P. ovale*
OVA-V F	TTTTGAAGAATATATTAGGATACATTATAG	*P. ovale Wallikeri*
OVA-V R	CATCGTTCCTCTAAGAAGCTTTACAAT	*P. ovale Wallikeri*
OVA P	CCTTTTCCCTATTCTACTTAATTCGCAATTCATG	*P. ovale curtisi*
OVA-V P	CCTTTTCCCTACTTAATTCGCTATTCATTG	*P. ovale Wallikeri*

#### Detection of species composition of infections.

All samples that were diagnosed as positive for the *Plasmodium* genus were further characterized for species composition as described by [68, 69], using a separate set of primers indicated in Table 1. The assays for characterization of *Plasmodium falciparum* and *P. malariae* had similar PCR reaction components and conditions as the genus-specific qPCR assay except for the primers used. Genus-specific primers were replaced respectively by the species-specific primers [30, 70], (Table 1). Each experiment included at least one reaction mixture without DNA as a negative control.

### Data management and statistical analysis

Descriptive analyses were used to describe categorical data comprising age categories, gender, and qPCR species results summarized as frequencies and proportions. Continuous variables, including age in years, were first assessed for normality using the Shapiro–Wilk test and then expressed as median together with their corresponding interquartile ranges (IQR) when non-normal distributions were observed. The geometric mean was used as a measure of central tendency for parasite density.

Descriptive statistics were employed to illustrate the burden of *Plasmodium* species among the participants with positive infections. For inferential statistics, proportions were compared using the Chi-square test as appropriate for the categorical data. Wilcoxon rank-sum test was used for non-parametric continuous variables. Logistic regression analysis was also conducted to assess the varying odds of *Plasmodium* species composition. All analyses were performed using R Studio version 4.5.1. Statistical analyses were performed at the 5% significance level and the corresponding 95% confidence interval (CI). The critical significance level was set at p ≤ 0.05.

## Results

A total of 741 samples comprising 437 (58.9%) of symptomatic participants and 304 (41.0%) of the asymptomatic participants were analyzed. The description of the study population is summarized in **Table 2**. Metadata was available for 733/741 participants though species analyses data was available for all the 741 samples. The median ages for all age categories and number of participants per age category varied significantly across the two infections presentations χ² test p = 0.000 (**Table 2**). The categories of less than 5 years were highest in symptomatic infections, 258 (79.4%) while over 18 years were highest in asymptomatic infections 81 (66.9%) (Table 2). Wilcoxon rank-sum test revealed a significantly higher age among the asymptomatic, median 9 years; IQR, 5–20.5 than symptomatic 4 years; IQR, 3–6, p = 0.000, r = 0.38. Gender was comparable between the symptomatic and asymptomatic infections. The geometric mean of parasite density per microliter of blood was significantly higher among the symptomatic (47504.83/ul, 95%CI 39131.25–57670.26) than asymptomatic infections (784.11/ul, 95%CI 784.11–1667.1) (Table 2).

**Table 2:** Participants Metadata. Metadata for symptomatic and asymptomatic *Plasmodium* infections.

Variable	Symptomatic	Asymptomatic	p value
**Age years:**			
**median (IQR)**	4 (3–6)	9 (5–21)	0.000
**Age category:**			
**<5 years (n)%**	258 (79.38)	67 (20.62)	0.000
**5-18 years (n)%**	131 (45.64)	156 (54.36)	
**>18 years (n)%**	40 (33.06)	81 (66.94)	
**Gender:**			
**Male (n)%**	195 (60.56)	127 (39.44)	0.323
**Female (n)%**	234 (56.93)	177 (43.07)	
**Parasite density:**			
**Geometric mean**	47504.83	1143.33	0.000
**(95% CI)**	(39131.25-57670.26)	(784.11-1667.107)	

*Plasmodium* co-infections analyses were conducted, and the species composition analyses detected the presence of single *Plasmodium* species in most infections and presence of two or three or four *Plasmodium* species in a subset of infections. Therefore, computing the raw frequencies of any detection of *Plasmodium* species yielded varying percentages that cumulatively exceeded 100%, consistent with the expected classification of infections containing multiple species under two or more species profiles. On this basis, *Plasmodium* species detection by qPCR assay showed that, 655/741 (88.3%) of the infections contained *P. falciparum,* 153/741 (20.7%) *P. ovalewallikeri,* 70/741 (9.4%) *P. malariae,* and 64/741 (8.7%) *P. ovalecurtisi* without consideration of the number of species per infection (**Table 3**). However, 12/741 (1.6%) of the samples that amplified for presence of *Plasmodium* genus did not amplify for any species.

**Table 3:** Overall *Plasmodium* species frequency. Overall *Plasmodium* species frequency detected in the samples.

Species	n	%
*P. falciparum*	655	88.4
*P. ovalewallikeri*	153	20.7
*P.malariae*	70	9.5
*P.ovalecurtisi*	64	8.7

Subsequent analysis with a focus on the presence or absence of more than one species per infection revealed that a total of 535/741 (72.2%) samples had single species while 194/741 (26.1%) had multiple species infections. *P. falciparum* was the most frequent single-species infection occurring in 470/535 (87.9%) infections, followed *by P. ovalewallikeri* in 34/535 (6.4%), *P. malariae* in 18/535 (3.4%), and *P. ovalecurtisi* in 13/535 (2.4%). Notably, all the *P. malariae* and *P. ovalecurtisi* single-species infections were detected among asymptomatic infections only. Multiple species infections included *Pf*/*Pow* 96/194 (49.5%), being dominant followed by *Pf*/*Pm* 41/194 (21.1%), *Pf*/*Poc* 30/194 (15.5), *Pf*/*Pow*/*Poc* 15/194 (7.7%), *Pm*/*Pow* 5/194 (2.6%), *Pf*/*Pm*/*Poc* 2/194 (1.0%), *Pm*/*Poc* 2/194 (1.0%), *Pf*/*Pm*/*Pow* 1/194 (0.5%), *Pm*/*Poc*/*Pow* 1/194 (0.5%), *Pow*/*Poc* 1/194 (0.5%).

### Symptomatic infections

Assessment of frequency of single *Plasmodium* species showed that *P. falciparum* 262/437 (60%) and *P. ovale wallikeri* 15/437 (3.4%) were the only single species detected among the symptomatic infections (Fig 2). The symptomatic individuals harbored multiple *Plasmodium* species, comprising 2 species; *PfPow* 92/437 (21.1%), *PfPm* 28/437 (6.4%), *PfPoc* 22/437 (5%), *PowPoc* 1/437 (0.2%), while three species included; *PfPowPoc* 15/437 (3.4%), *PfPmPoc* 2/437 (0.5%) (Fig 2). Further, neither *P. ovalecurtisi* nor *P. malariae* occurred as a single species infection among symptomatic participants during this study period (Fig 2).

**Fig 2:**
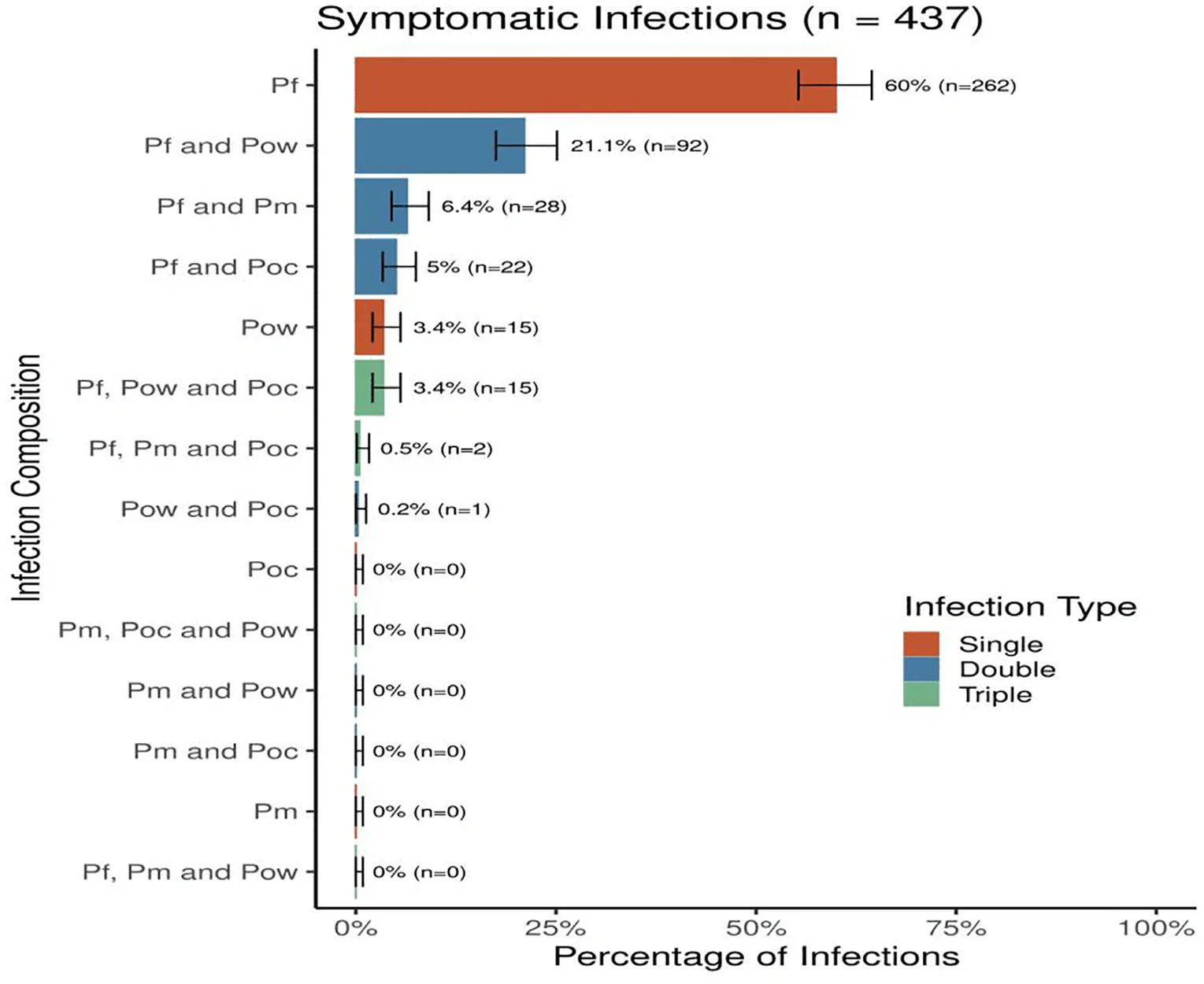
*Plasmodium* single species and mixed species frequency among symptomatic infections by molecular diagnosis. Symptomatic infections refer to infections that generate malarial parasitemia alongside symptoms.

### Asymptomatic infections

Single *Plasmodium* species infections included *P. falciparum* detected in 218/304 (71.7%) of the asymptomatic infections as the most prevalent species, followed by *P. ovalewallikeri* 18/304 (5.9%), *P. malariae* 16/304 (5.3%), and *P. ovalecurtisi* 15/304 (4.9%), respectively (Fig 3). Multiple species infections comprised of double species; *PfPm* 14/304 (4.6%), *PfPoc* 8/304 (2.6%), *PmPow* 5/304 (1.6%), *PfPow* 5/304 (1.6%), *PmPoc* 2/304 (0.7%), or triple species *PfPmPow* 2/304 (0.7%) and *PmPocPow* 1/304 (0.3%) infections (Fig 3).

**Fig 3:**
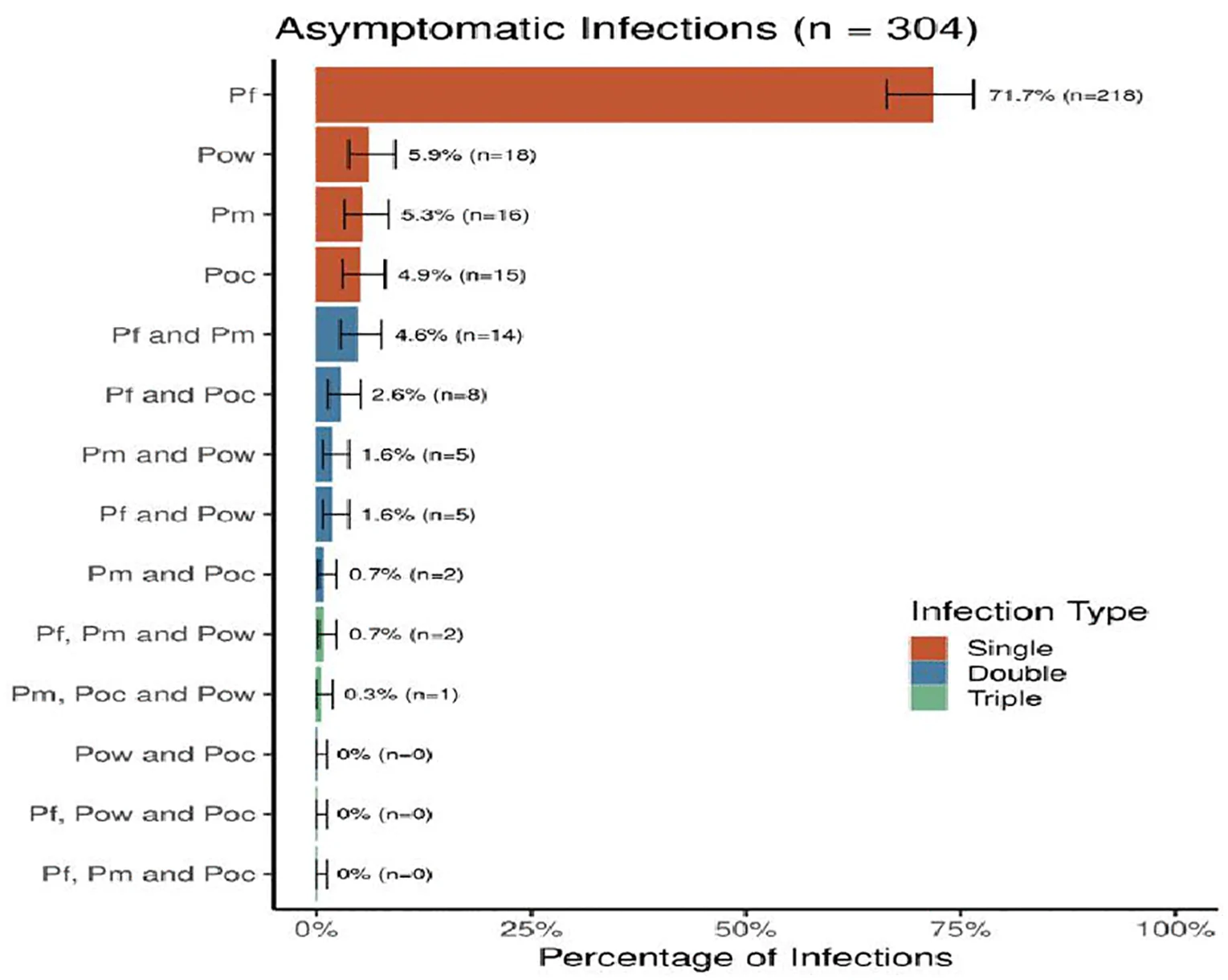
*Plasmodium* mixed and single species frequency among asymptomatic infections by molecular diagnosis. Asymptomatic *Plasmodium* parasite carriers are the infections that generate malarial parasitemia in the absence of fever or other classical symptoms and signs, in otherwise healthy individuals.

The *P. falciparum* single-species infections were more prevalent in asymptomatic than symptomatic individuals (Figs 2 and 3). Multiple species were more likely to be symptomatic than asymptomatic infections. Assessment of the role of infection composition in disease presentation showed that *P. falciparum* with *P. ovalewallikeri*; *P. falciparum* with *P. ovalecurtisi,* and *P. falciparum, P. ovalewallikeri* with *P. ovalecurtisi* mixed-species infections had higher odds of progressing to symptomatic infection than remaining asymptomatic (Fig 4). The mixed species had increased odds of symptomatic infection (Fig 4).

**Fig 4:**
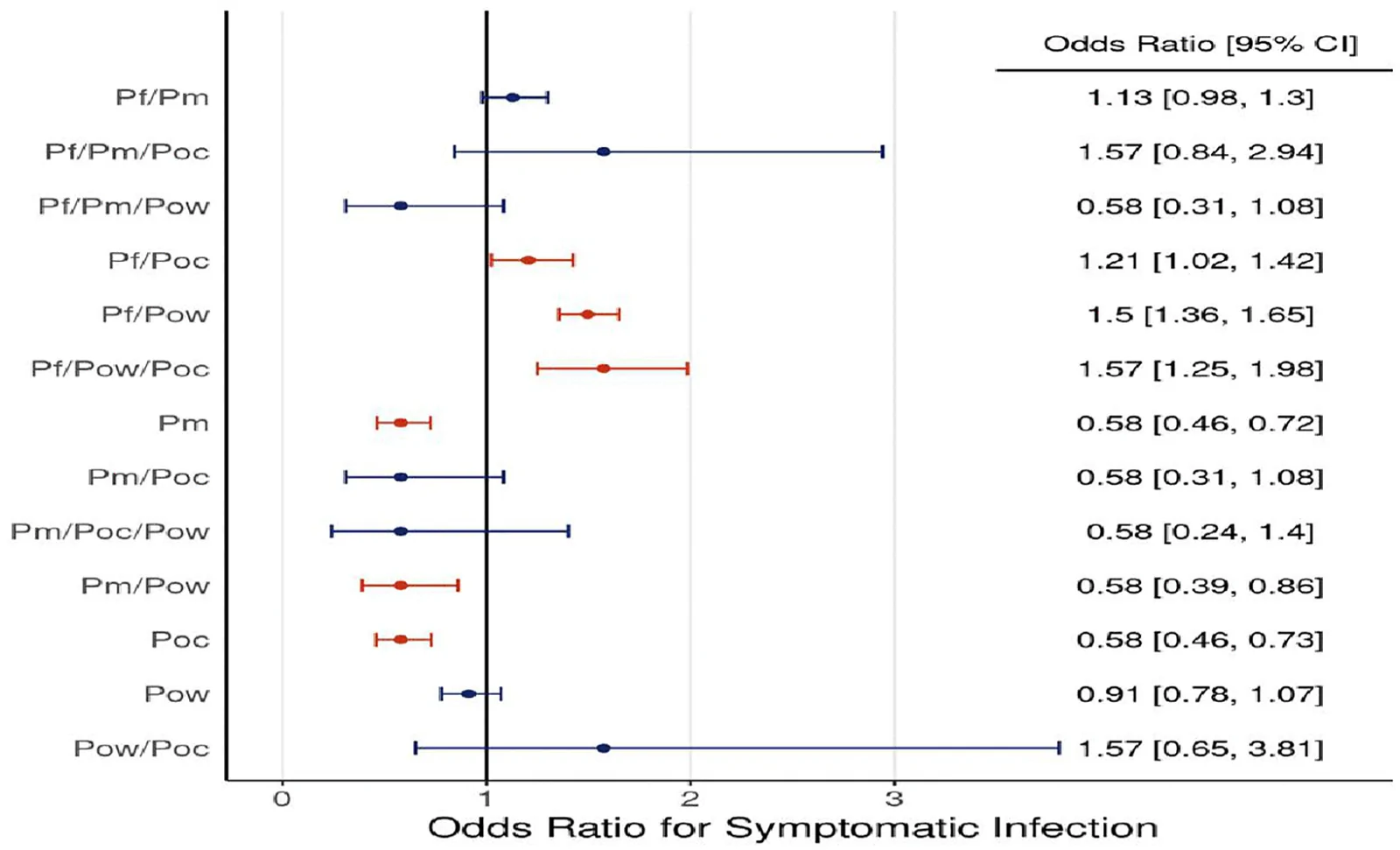
Odds of *Plasmodium* species composition in infection presentation. The blue represents no significant association, while the red color represents a significant association.

## Discussion

This study concurrently compared the prevalence of *Plasmodium* species among symptomatic individuals seeking treatment at the Kombewa Sub-County hospital versus asymptomatic individuals in households within the same geographic location that doubles up as the catchment of the hospital using qPCR. About 13% and 26% prevalence of multiple species was detected among asymptomatic and symptomatic infections, respectively. Studies in West and Central Africa focusing on school-going children have also shown a higher burden of non-*falciparum* species in asymptomatic *Plasmodium* parasite carriers [52, 53]. This finding is contrary to a recent meta-analysis showing no difference in the prevalence of either *P. malariae* or *P. ovale spp* between symptomatic and asymptomatic individuals [71]. Additionally, large-scale studies of non-*falciparum* parasites report conflicting results for those from West Africa [72] versus those from central or East Africa [73]. These varying observations underscore the peculiarity of *Plasmodium* infection presentation across different disease endemicities and are evocative of the need for characterizing species composition in diverse disease geo-ecotypes for effective targeting.

The occurrence of *Plasmodium* parasitemia in the absence of classical malaria symptoms, and the mechanisms that influence the transition from an asymptomatic infection to symptomatic infection, remain subjects of ongoing debate. A prospective study by Chen and coworkers showed that some individuals harboring *Plasmodium* parasites can either remain healthy for an unlimited amount of time and are referred to as asymptomatic [23] while others progress to disease with malaria symptoms within 7–85 days and seek for the treatment depending on various factors, including the species composition of the infection [49, 60, 74, 75]. This study detected a higher frequency of mixed species infection among symptomatic than asymptomatic individuals, an observation that appears to align with the theory that harboring multiple species potentiates the transition of infections to the symptomatic phase.

Studies on inter-parasite interactions within human hosts suggest that parasites often interact with unrelated competitors within the host [76] and use this mechanism to prioritize the resource allocation framework, opting to increase replication to occupy most of the red blood cells at the expense of the development of sexual stages as one of the exclusion strategies. The changes in resource utilization resulting from the presence of the additional species have been associated with modulation of the host response [61, 77–79], toward either the persistence of the asymptomatic phase of the infection [80] or progression to symptomatic disease in the presence of virulence-enhancing species mixtures [81]. The detection of a higher frequency of multiple species infections among the symptomatic than asymptomatic, with a reciprocal higher frequency of single species infections in asymptomatic than symptomatic, by this study appears to suggest that infection with more than one species is likely to progress to a more symptomatic state than single-species infections as reported elsewhere [28, 29]. It was, however, surprising that asymptomatic individuals harbored more diverse species profiles than symptomatic individuals based on PCR-based diagnosis.

The composition of mixed species infections varied significantly between the symptomatic and asymptomatic infections. For example, there was a ten-fold higher prevalence of co-infections containing *P. falciparum* and *P. ovalewallikeri* among the symptomatic individuals than among the asymptomatic individuals. A similar pattern of frequency was observed among infections containing *P. falciparum, P. ovalewallikeri,* plus *P. ovalecurtisi,* contrary to *P. falciparum/P. ovalecurtisi, or P. falciparum/P. malariae* or either of these species in single-species infections, an observation that may suggest between-species interactions.

Evidence from malaria ecology indicates that the acquisition of an additional *Plasmodium* species within a host can modify the within-host ecological environment by introducing competition for limited resources such as red blood cells. For example, in mixed-species infections involving human malaria parasites, including *P. falciparum* and *P. vivax*, parasites may compete for host erythrocytes and other resources, which can alter parasite replication dynamics and suppress the density of competing species. These competitive interactions may consequently influence parasite fitness, disease outcomes, and transmission potential [82]. Parasite biology studies show that *P. falciparum* attacks all ages of red blood cells, hence it has been hypothesized to be a “generalist” while *P. ovale* and *P. malariae* attack younger and older red blood cells, respectively, and are hypothesized to be specialists [80, 81, 83].

Studies show that in mixed-species infections, the presence of an RBC *Plasmodium* species regarded as “generalists” enhances the density of RBC specialists [79]. A study monitoring blood levels of tumor necrosis factor-alpha (TNF-alpha), lactate dehydrogenase (LDH), Thrombin-antithrombin III (TAT) and elastase as drivers of malaria symptoms through modulation of host immune response among uncomplicated malaria was conducted [81, 84]. The study further noted that these parameters were higher per parasitized *P. ovale* erythrocyte levels than *P. falciparum*, with the former attaining full-strength host response at lower parasitemia than the latter [81]. Observation of significantly higher frequency *P. falciparum* and *P. ovalewallikeri* among naturally acquired symptomatic than asymptomatic infection by this study suggests that parasitemia increases in *P. ovalewallikeri,* a specialist, given the presence of *P. falciparum* a generalist, could be contributing to elevated immune response leading to the occurrence of symptoms among these infections. This phenomenon, which is absent in *P. ovalecurtisi* infections, highlights the biological differences between the two *P. ovale* species. Notably, *P. ovalewallikeri* single-species infections were detected among symptomatic as well as asymptomatic infections while *P. ovalecurtisi* alone was not detected among the symptomatic, suggesting that *P. ovalewallikeri* alone causes symptoms more frequently than *P. ovalecurtisi* among individuals.

Single-species infections of *P. falciparum, P. malariae, or P. ovalecurtisi* were mostly detected among asymptomatic individuals than symptomatic. Notably, *P. ovalecurtisi* single-species infections were detected in 4.8% of the asymptomatic infections while none was detected in symptomatic infections. Additionally, infections comprising *P. malariae*/*P. ovalecurtisi* or *P. malariae*/*P. ovalewallikeri* were detected in asymptomatic infections at less than 2% frequency while none were detected among symptomatic infections. *P. malariae* co-infections with *P. ovalecurtisi* or *P. ovalewallikeri* have been associated with low pyrogenic thresholds, occurring in less than 2% of the symptomatic cases [80]. This infection characteristic is a challenge for malaria elimination since the chronically infected individual would remain obscure to healthcare facilities while still capable of transmitting the parasite to mosquitoes and therefore sustaining transmission to humans. Such infections have been proposed as the last battlefield for malaria elimination [85] since they will also remain undetected by the histidine-rich protein based malaria rapid diagnostic test that are expedient for population-based strategies of bringing down diseases.

This study has some limitations. The study depended on the vital signs of the participants at the time of enrolment such as fever ≥37.5 °C or a history of fever within the preceding 48 hours accompanied by symptoms such as chills, headache, malaise, or sweating, to be categorized as symptomatic participants, while those without these signs were categorized as asymptomatic. Because of the study design, it was difficult to determine when, specifically, the time-point in the previous period preceding enrolment day, at which either the asymptomatic or symptomatic individuals acquired the infection. Consequently, it was not possible to establish whether individuals with asymptomatic infections would subsequently develop symptoms as parasite densities increased to the pyrogenic threshold, or whether the infections in these individuals were chronic and would remain asymptomatic. It was again not possible to ascertain which of the infecting species in individuals harbouring multiple species infections came first and which one came later. This, therefore, made it harder to tell whether there was protectiveness proffered by some species or immune silencing by other species to delay symptoms, or associate entry of a given species with the onset of symptoms. Further, the study was not able to filter out and show within-host response to varying species composition of infections or between-species interactions within each of the mixed infection profiles to filter out specific parasite species-specific biology that infer implication to the progression of disease or epidemiology of each of the species versus presently deployed malaria interventions. Finally, host immunity was not characterized in this study because it was not one of the endpoints for the study.

## Conclusion

These findings in a malaria-endemic zone suggest that intervention strategies need to consider the species composition of asymptomatic *Plasmodium* parasite infections to progress the region to the elimination phase. The ten-fold higher frequency of *P. ovalewallikeri* species among the symptomatic than asymptomatic infections and higher *P. falciparum* single-species infections in the asymptomatic than symptomatic appears to associate this infection composition with higher likelihood of symptoms. Interventions need to focus on the mixed infections asymptomatic *Plasmodium* parasite carriers since these two serves as silent reservoirs for transmission and are a lynchpin to malaria elimination in a given region.
