# Supplementary material for: High prevalence of Plasmodium ovalewallikeri in co-infections with Plasmodium falciparum in symptomatic than asymptomatic Plasmodium parasite infections in Kisumu, Kenya
Source: PLoS One. 2026 Oct 7;21(10):e0334188. doi: 10.1371/journal.pone.0334188 (PMC13645224; doi:10.1371/journal.pone.0334188)
Supplement: Supplementary file 1 [file pone.0334188.s002.pdf]

PLOS One

High prevalence of Plasmodium ovalewallikeri in co-infections with Plasmodium falciparum in symptomatic than asymptomatic malaria parasite carriers in Kisumu, Kenya

--Manuscript Draft--

Manuscript Number:	PONE-D-25-45620
Article Type:	Research Article
Full Title:	High prevalence of Plasmodium ovalewallikeri in co-infections with Plasmodium falciparum in symptomatic than asymptomatic malaria parasite carriers in Kisumu, Kenya
Short Title:	Varying burden of Plamodium ovale and Plasmodium malariae based on infection presentation
Corresponding Author:	Hoseah M. Akala, PhD<br>Kenya Medical Research Institute<br>Kisumu, Kenya, KENYA
Keywords:	Plasmodium species; symptomatic; asymptomatic
Abstract:	Plasmodium falciparum (Pf), Plasmodium ovalewallikeri (Pow), Plasmodium ovalecurtisi (Poc), and Plasmodium malariae (Pm) coexists in Kenya sympatrically. Some infections progress to classical symptoms of malaria prompting hospital visits while others remain asymptomatic. This study compared the composition of Plasmodium species among symptomatic versus asymptomatic individuals in Kombewa, Kenya. Blood samples from 741 individuals, comprising 437 symptomatic at Kombewa hospital and 304 asymptomatic from households within the hospital's catchment area between 2015 and 2016, were analyzed for Plasmodium species composition using small subunit ribosomal RNA gene (ssrRNA). Broadly, 741 mostly contained Pf 668 (90·15%) followed by Pow 154 (20·78%), Pm 70 (9·45%) and Poc 64 (8·7%). Of these, 544 (73·41%) infections contained single-species while 197 (26·59%) contained multiple-species. The frequency of Plasmodium species among the symptomatic and asymptomatic infections were Pf 421 (96·3%) and 247 (81·25%), Pow 123 (28·3%) and 31 (10·19%), Poc 40 (9·2%) and 26 (8·55%) while Pm was 30(6·8%) and 40 (13·2%), respectively. Notably, Pf, Pm, and Poc mixed-species infections were more frequent among asymptomatic than symptomatic individuals (OR 0.08, 95% CI ranged [0.02 – 0.36, P = 0.0008). Pm 18 (2.43%) and Poc 13 (1.75%) single-species infections occurred among asymptomatic, yet none were among symptomatic, suggesting that these single-species infections are often benign. Significantly higher multiple species infections containing Pow (OR 15.3, 95% CI range [6.1 – 38.3 and P = 0.0001) was observed among symptomatic than asymptomatic individuals. Single-species infections of Pf, Pm, or Poc were mostly detected among asymptomatic than symptomatic. The high frequency of nonfalciparum infections calls for deployment of interventions that target broad species, especially those on asymptomatic infection for progressing regions to the pre-elimination phase. Significantly higher prevalence of multiple-species infections in symptomatic cases necessitate evaluation of the role of infecting species in disease progression and modulation of treatment-seeking behavior in endemic regions.
Order of Authors:	Hoseah M. Akala, PhD
	Jackline A. Juma, BSc
	Oliver J. Watson, PhD
	Dennis Juma, MSc
	Benjamin H. Opot, BSc
	Gladys Chemwor, MSc
	Mary Mutahi, BSc
	Raphael Okoth, BSc

Powered by Editorial Manager® and ProduXion Manager® from Aries Systems Corporation

	Edwin W. Mwakio, BSc
	Redemptah Yeda, MSc
	Charles O. Okello, BSc
	Agnes Cheruiyot, MSc
	Duke Omariba, BSc
	Iiliana Dayo, MSc
	Hellen A. Ogollah, BSc
	Gladys Biwott, MSc
	Charles Adega, BSc
	Fredrick Oluoch, MBChB
	Vincent Omondi, MBChB
	George Otieno, BSc
	Edwin Kamau, PhD
	Ben Andagalu, MSc
	Peter Sifuna, MSc
	Amanda Roth, PhD
	Sammy Khagayi, PhD
	Erick C. Grages, MBChB
	Bernherds Ogutu, PhD
**Additional Information:**	
**Question**	**Response**
**Financial Disclosure**<br><br>Enter a financial disclosure statement that describes the sources of funding for the work included in this submission. Review the submission guidelines for detailed requirements. View published research articles from *PLOS ONE* for specific examples.<br><br>This statement is required for submission and **will appear in the published article** if the submission is accepted. Please make sure it is accurate.	Yes

**Funded studies**<br>Enter a statement with the following details:<br>• Initials of the authors who received each award<br>• Grant numbers awarded to each author<br>• The full name of each funder<br>• URL of each funder website<br>• Did the sponsors or funders play any role in the study design, data collection and analysis, decision to publish, or preparation of the manuscript?<br><br>Did you receive funding for this work?	
Please add funding details.<br>as follow-up to "**Financial Disclosure**<br><br>Enter a financial disclosure statement that describes the sources of funding for the work included in this submission. Review the submission guidelines for detailed requirements. View published research articles from *PLOS ONE* for specific examples.<br><br>This statement is required for submission and **will appear in the published article** if the submission is accepted. Please make sure it is accurate.<br><br>**Funded studies**<br>Enter a statement with the following details:<br>• Initials of the authors who received each award<br>• Grant numbers awarded to each author<br>• The full name of each funder<br>• URL of each funder website<br>• Did the sponsors or funders play any role in the study design, data collection and analysis, decision to publish, or preparation of the manuscript?<br><br>Did you receive funding for this work?"	Funding for this study was provided by the Armed Forces Health Surveillance Branch and its Global Emerging Infections Surveillance Section (Grant P0209_15_KY). The funder of the study played no role in study design, data collection, data analysis, data interpretation, or writing of the report.
Please select the country of your main research funder (please select carefully as in some cases this is used in fee calculation).	UNITED STATES - US

as follow-up to "**Financial Disclosure**<br><br>Enter a financial disclosure statement that describes the sources of funding for the work included in this submission. Review the submission guidelines for detailed requirements. View published research articles from *PLOS ONE* for specific examples.<br><br>This statement is required for submission and **will appear in the published article** if the submission is accepted. Please make sure it is accurate.<br><br>**Funded studies**<br>Enter a statement with the following details:<br>• Initials of the authors who received each award<br>• Grant numbers awarded to each author<br>• The full name of each funder<br>• URL of each funder website<br>• Did the sponsors or funders play any role in the study design, data collection and analysis, decision to publish, or preparation of the manuscript?<br><br>Did you receive funding for this work?"	
**Competing Interests**<br><br>Use the instructions below to enter a competing interest statement for this submission. On behalf of all authors, disclose any competing interests that could be perceived to bias this work—acknowledging all financial support and any other relevant financial or non-financial competing interests.<br><br>This statement is required for submission and **will appear in the published article** if the submission is accepted. Please make sure it is accurate and that any funding sources listed in your Funding Information later in the submission form are also declared in your Financial Disclosure statement.	The authors have declared that no competing interests exist.

View published research articles from *PLOS ONE* for specific examples.<br><br>**NO authors have competing interests**<br><br>Enter: *The authors have declared that no competing interests exist.*<br><br>**Authors with competing interests**<br><br>Enter competing interest details beginning with this statement:<br><br>*I have read the journal's policy and the authors of this manuscript have the following competing interests: [insert competing interests here]*<br><br>* typeset	
**Ethics Statement**<br><br>Enter an ethics statement for this submission. This statement is required if the study involved:<br><br>• Human participants<br>• Human specimens or tissue<br>• Vertebrate animals or cephalopods<br>• Vertebrate embryos or tissues<br>• Field research<br><br>Write "N/A" if the submission does not require an ethics statement.<br><br>General guidance is provided below. Consult the submission guidelines for detailed instructions. **Make sure that all information entered here is included in the Methods section of the manuscript.**	These studies were approved by Kenya Medical Research Institute (KEMRI), Scientific and Ethics Review Unit (SERU), and Walter Reed Army Institute of Research (WRAIR) institutional review boards (protocol numbers: KEMRI 1330/WRAIR 1384 and KEMRI 2721/ WRAIR 2064. A written informed consent /assent was obtained from each of the study participants. The confidentiality of information obtained from study participants was assured by using unique identifiers.

Powered by Editorial Manager® and ProduXion Manager® from Aries Systems Corporation

**Format for specific study types**<br><br>**Human Subject Research (involving human participants and/or tissue)**<br>• Give the name of the institutional review board or ethics committee that approved the study<br>• Include the approval number and/or a statement indicating approval of this research<br>• Indicate the form of consent obtained (written/oral) or the reason that consent was not obtained (e.g. the data were analyzed anonymously)<br><br>**Animal Research (involving vertebrate animals, embryos or tissues)**<br>• Provide the name of the Institutional Animal Care and Use Committee (IACUC) or other relevant ethics board that reviewed the study protocol, and indicate whether they approved this research or granted a formal waiver of ethical approval<br>• Include an approval number if one was obtained<br>• If the study involved *non-human primates*, add *additional details* about animal welfare and steps taken to ameliorate suffering<br>• If anesthesia, euthanasia, or any kind of animal sacrifice is part of the study, include briefly which substances and/or methods were applied<br><br>**Field Research**<br><br>Include the following details if this study involves the collection of plant, animal, or other materials from a natural setting:<br>• Field permit number<br>• Name of the institution or relevant body that granted permission	
**Data Availability**<br><br>Authors are required to make all data underlying the findings described fully available, without restriction, and from the time of publication. PLOS allows rare exceptions to address legal and ethical concerns. See the PLOS Data Policy and FAQ for detailed information.	Yes - all data are fully available without restriction

A Data Availability Statement describing where the data can be found is required at submission. Your answers to this question constitute the Data Availability Statement and **will be published in the article**, if accepted.<br><br>**Important:** Stating 'data available on request from the author' is not sufficient. If your data are only available upon request, select 'No' for the first question and explain your exceptional situation in the text box.<br><br>Do the authors confirm that all data underlying the findings described in their manuscript are fully available without restriction?	
**Describe where the data may be found in full sentences. If you are copying our sample text, replace any instances of XXX with the appropriate details.**<br><br>• If the data are **held or will be held in a public repository**, include URLs, accession numbers or DOIs. If this information will only be available after acceptance, indicate this by ticking the box below. For example: *All XXX files are available from the XXX database (accession number(s) XXX, XXX.).*<br>• If the data are all contained **within the manuscript and/or Supporting Information files**, enter the following: *All relevant data are within the manuscript and its Supporting Information files.*<br>• If neither of these applies but you are able to provide **details of access elsewhere**, with or without limitations, please do so. For example:<br><br>*Data cannot be shared publicly because of [XXX]. Data are available from the XXX Institutional Data Access / Ethics Committee (contact via XXX) for researchers who meet the criteria for access to confidential data.*<br><br>*The data underlying the results presented in the study are available from (include the name of the third party*	All relevant data are within the manuscript and its Supporting Information files.

and contact information or URL).<br>• This text is appropriate if the data are owned by a third party and authors do not have permission to share the data.<br><br>* typeset	
Additional data availability information:	Tick here if your circumstances are not covered by the questions above and you need the journal's help to make your data available.

Powered by Editorial Manager® and ProduXion Manager® from Aries Systems Corporation

Manuscript

Click here to access/download;Manuscript;Jackline Juma et al 20250820_Final_Manuscript.docx

High prevalence of *Plasmodium ovalewallikeri* in co-infections with *Plasmodium falciparum* in symptomatic than asymptomatic malaria parasite carriers in Kisumu, Kenya

Jackline Juma[1], Oliver J. Watson[4], Dennis Juma[1], Benjamin Opot[1], Gladys Chemwor[1], Mary Mutahi[3], Raphael Okoth[1], Edwin W. Mwakio[1], Redemptah Yeda[1], Charles Okello[1], Agnes Cheruiyot[1], Duke Omariba[1], Liliana Dayo[9], Hellen A. Ogola[9], Gladys Biwott[1], Charles Adega[1], Fredrick Oluoch[9], Vincent Omondi[9], George Otieno[9], Edwin Kamau[6], Ben Andagalu[1], Peter Sifuna[1], Amanda Roth[7], Sammy Khagayi [8], Eric C. Garges[5], Bernhards Ogutu[2] Hoseah M. Akala[1]*

[1]Department of Emerging and Infectious Diseases, Walter Reed Army Institute of Research-Africa/Centre for Clinical Research, Kenya Medical Research Institute, P. O. Box 54 – 40100, Kisumu, Kenya

[2] Centre for Clinical Research, Kenya Medical Research Institute

[3]The Kenya Medical Research Institute (KEMRI) / Wellcome Trust Research Programme, Centre for Geographic Medicine Research-Coast, Kilifi, Kenya.

[4]Medical Research Council, Centre for Global Infectious Disease Analysis, Department of Infectious Disease Epidemiology, Imperial College London

[5]Walter Reed Army Institute of Research - Africa

[6]Department of Pathology and Laboratory Medicine, Tripler Amy Medical Center, Honolulu, HI 96589, United States of America.

[7]Medical Communications for Combat Casualty Care, 1540 Porter Street, Fort Detrick MD21702.

[8] Centre for Global Health Research, Kenya Medical Research Institute, Kisumu, Kenya

[9]Department of Health County Government of Kisumu, Ministry of Health, Kenya.

*Correspondence to Dr. Hoseah M. Akala, Department of Emerging and Infectious Diseases Walter Reed Army Institute of Research-Africa/Centre for Clinical Research, Kenya Medical Research Institute, P. O. Box 54 – 40100, Kisumu, Kenya. Tel. +254722329845. E-mail: Hoseaakala@yahoo.com ; Hosea.akala@usamru-k.org

Abstract

Plasmodium falciparum (*Pf*), *Plasmodium ovalewallikeri* (*Pow*), *Plasmodium ovalecurtisi* (*Poc*), and *Plasmodium malariae* (*Pm*) coexists in Kenya sympatrically. Some infections progress to classical symptoms of malaria prompting hospital visits while others remain asymptomatic. This study compared the composition of *Plasmodium* species among symptomatic versus asymptomatic individuals in Kombewa, Kenya. Blood samples from 741 individuals, comprising 437 symptomatic at Kombewa hospital and 304 asymptomatic from households within the hospital's catchment area between 2015 and 2016, were analyzed for *Plasmodium* species composition using small subunit ribosomal RNA gene (ssrRNA). Broadly, 741 mostly contained *Pf* 668 (90·15%) followed by *Pow* 154 (20·78%), *Pm* 70 (9·45%) and *Poc* 64 (8·7%). Of these, 544 (73·41%) infections contained single-species while 197 (26·59%) contained multiple-species. The frequency of *Plasmodium* species among the symptomatic and asymptomatic infections were *Pf* 421 (96·3%) and 247 (81·25%), *Pow* 123 (28·3%) and 31 (10·19%), *Poc* 40 (9·2%) and 26 (8·55%) while *Pm* was 30(6·8%) and 40 (13·2%), respectively. Notably, *Pf*, *Pm*, and *Poc* mixed-species infections

were more frequent among asymptomatic than symptomatic individuals (OR 0.08, 95% CI ranged [0.02 – 0.36, P = 0.0008). Pm 18 (2.43%) and Poc 13 (1.75%) single-species infections occurred among asymptomatic, yet none were among symptomatic, suggesting that these single-species infections are often benign. Significantly higher multiple species infections containing Pow (OR 15.3, 95% CI range [6.1 – 38.3 and P = 0.0001) was observed among symptomatic than asymptomatic individuals. Single-species infections of *Pf*, *Pm*, or *Poc* were mostly detected among asymptomatic than symptomatic. The high frequency of nonfalciparum infections calls for deployment of interventions that target broad species, especially those on asymptomatic infection for progressing regions to the pre-elimination phase. Significantly higher prevalence of multiple-species infections in symptomatic cases necessitate evaluation of the role of infecting species in disease progression and modulation of treatment-seeking behavior in endemic regions.

Key words

Plasmodium species, symptomatic, asymptomatic

Introduction

Human malaria infection is attributed to six *Plasmodium* species namely, *falciparum, ovalecurtisi, ovalewallikeri, malariae, vivax* and *knowlesi* (1, 2) (3). The burden of these species in natural infections varies globally (4) along with their key vectors (5). *P. falciparum* is responsible for the most severe form of disease and is the most prevalent in Sub-Saharan Africa (6) where it causes the highest number of fatalities globally (6) *Plasmodium vivax* is the second most dominant species that causes recurring malaria with the highest burden in Asia and South America and in Africa it is mainly in Ethiopia and Sudan (7). The less prevalent species *P. ovalewallikeri*, and *P. ovalecurtisi* occur primarily in Africa (8, 9). *P. malariae* is predominantly found in South America, Asia, and Africa, and occurs mainly as mixed infections with *P. falciparum* at a lower frequency (10).

P. falciparum infection occurs predominantly as mono-infection or sympatrically with *P. malariae* and *P. ovalewallikeri*, and *P. ovalecurtisi* in Kenya. This co-endemicity of species leads to the occurrence of blood-stage mixed *Plasmodium* species infections (11) which are mostly acquired from separate inoculations when a recently acquired infection of one species might supervene a chronic infection with another (12) or simultaneous inoculation of the sporozoites of the two or more species from a doubly infected *Anopheline* mosquito (13) (12) (14). Studies have highlighted variations in the clinical presentation of disease per species (15-17) (18-20) relative to the unique parasite biology of each malaria species. Emerging reports of increasing non-falciparum prevalence during a period of improved access to accurate diagnosis and prompt treatment necessitate an assessment of the

species composition of infections among individuals seeking treatment in healthcare facilities with symptoms versus those in households without symptoms in order to halt the trend.

Clinical symptoms of malaria starts between 7 to 85 days after initial infection depending on the infecting *Plasmodium* species (21-23) (24). Parasitemia may also persist in susceptible individuals for up to eight years before the onset of symptoms (25) (26) (27) (28). Initial symptoms of malaria may include a high temperature of 38°C and above, muscle pains and generally feeling un-well often characterized by recurrent fever and chills (24), which often trigger treatment-seeking response (29) once parasite replication becomes synchronous. In some cases, the infection may generate malarial parasitemia in the absence of fever or other classical symptoms and signs, in otherwise healthy individuals, therefore regarded as asymptomatic malaria parasite carriers (30-32). Asymptomatic malaria accounts for up to 60% of infections (33-36) in some regions. It has been suggested that infections remain asymptomatic due to sustained submicroscopic peripheral parasitemia (37, 38), older age, and therefore partial immunity of the infected individual (39, 40) and varying malaria pyrogenic threshold (41, 42). In a 2011 study, the complex interactions between infecting *Plasmodium* species (17, 41) were shown to affect the disease outcome such as the occurrence of symptoms (43) and transmissibility. As global efforts continue to focus on malaria elimination, the role of this interaction in either restraining or fostering infection progression to symptomatic necessitating treatment seeking in different settings of disease burden remains unclear.

The World Health Organization (WHO) guidelines on the treatment of malaria is based on parasitological detection of infection (44, 45) and is globally adopted (46) through national treatment guidelines. The WHO scaled-up accurate diagnosis and treatment of

malaria (47) regardless of case presentation whether symptomatic or asymptomatic (29, 48, 49). This guideline relies on the gold standard light microscopy and enhanced Rapid diagnostic test (RDT) diagnostic methods with limitation in low-level parasitemia (30, 50). The RDTs are limited in the estimation of parasitemia(51) distinguishing infecting species (52) which are common among asymptomatic individuals (53) and therefore likely to underestimate the burden and complexity of infection in a population (54, 55). The recent innovation of molecular methods of diagnosis of subclinical submicroscopic *Plasmodium* infections (56, 57) that are a reservoir for transmission (58) becomes an emerging surveillance tool for malaria elimination strategies (59-62). In a region with natural infections comprising more than one *Plasmodium* species (63, 64), there is uncertainty about whether infection with multiple species reduces or promotes the incidence of symptoms in the infected individual as a means of maintaining transmissibility. Our previous study showed the prevalence of *Plasmodium* species co-infections changes among symptomatic individuals seeking treatment at selected hospitals across Kenya (65), but did not describe the burden among asymptomatic infections. Given the varying disease progression among *Plasmodium* species such as longer period of case management for relapsing species, estimating of the burden of species in the population is useful in informing choice of intervention needed to lower malaria in line with the WHO goal that targets 90% reduction of malaria by 2030. The aim of this study was to estimate the composition of *Plasmodium* species among symptomatic versus asymptomatic individuals in Kisumu County in western Kenya.

Materials and Methods

Ethical consideration

These studies were approved by Kenya Medical Research Institute (KEMRI), Scientific and Ethics Review Unit (SERU), and Walter Reed Army Institute of Research (WRAIR) institutional review boards (protocol numbers: KEMRI 1330/WRAIR 1384 and KEMRI 2721/ WRAIR 2064. A written informed consent /assent was obtained from each of the study participants. The confidentiality of information obtained from study participants was assured by using unique identifiers.

Study sites and sampling collection

The study was conducted within the Kombewa Health and Demographic Surveillance System (HDSS) is in a rural part of Kisumu County, Western Kenya according to a study by Sifuna and co-workers. The area is categorized as a rural Lake Endemic Zone with perennial and intense malaria transmission as per the National Malaria Control Programme 2016 (NMCP).

This sub-study analysed *Plasmodium* positive samples obtained from the two studies that were running concurrently between 2015 and 2016. The first study was entitled "Epidemiology of malaria and drug sensitivity patterns in Kenya" That commenced from 1st September 2015 to 1st June 2016. This study enrolled symptomatic patients aged six months and above presenting at Kombewa sub-county hospital with symptoms of uncomplicated malaria and testing positive by malaria RDT and microscopy. Each participant provided 2-3 ml of whole blood for malaria parasite species genotyping alongside other endpoints of the main study reported elsewhere (66, 67). All *Plasmodium*-positive cases were treated with artemether-lumefantrine (AL) (Coartem®), as per the Kenya Ministry of Health's

recommended case management guidelines for uncomplicated malaria. The healthcare workers administered and observed taking of the first dose of AL 20mg/120mg tablets.

The second study was entitled, “Baseline Epidemiology of Malaria Gametocyte Carriage and Transmission Dynamics in a Holoendemic Transmission Setting in Kisumu County, Western Kenya” that commenced on 18th March 2015 to 1st February 2016. The study sought to estimate the level of gametocyte carriage in the study population by PCR. Members of selected households within Kisumu West sub-county that is the main catchment for the Kombewa sub-county hospital (**Fig 1_Map of Kombewa**) willing to be tested for the presence of malaria parasites were enrolled into the study upon providing written informed consent/assent. During this household visit, vital signs were measured to rule out symptoms of malaria. Based on these readouts, each of the otherwise healthy participant provided 4 ml of whole blood for diagnosis of malaria. Each sample was tested within 24 hours and all participants found to have malaria were treated with the attending healthcare worker administering the first dose of AL 20mg/12mg tablets.

Fig 1: Map showing Kombewa region where Kombewa Sub County hospital is located.

Molecular Assay

Nucleic acids extraction

Nucleic acids were extracted from blood samples obtained from individuals enrolled in the two groups using a commercial extraction kit (QIAamp DNA Blood Mini Kit, Qiagen) as recommended by the manufacturer's instructions.

Plasmodium genus diagnosis by molecular methods

The nucleic acids were used for malaria diagnosis using *Plasmodium* genus-specific qPCR assay as previously described (68, 69). PCR amplified the *Plasmodium* genus 18S ribosomal ribonucleic acid (rRNA) gene using primers listed in **Table 1**. The RN*ase* P gene, which is a human housekeeping gene, was used as an extraction and qPCR assay control.

Speciation

All samples that were diagnosed as positive for the *Plasmodium* genus were further characterized for species composition as described by (68, 69) using a separate set of primers indicated in **Table 1.** The assays for characterization of *Plasmodium falciparum* and *P. malariae* had similar PCR reaction components and conditions as the Genus-specific qPCR assay except for the primers used. Genus-specific primers were replaced respectively by the species-specific primers (56, 70) **(Table 1**). Each experiment included at least one reaction mixture without DNA as a negative control.

Table 1: Primers and probes used for screening and identification of *Plasmodium* species

Primers Probes	Sequence 5'-3'	Target genus/species
PLU F	GCTCTTTCTTGATTTCTTGGATG	*Plasmodium* spp.
PLU R	AGCAGGTTAAGATCTCGTTCG	*Plasmodium* spp.
PLU P	ATGGCCGTTTTTAGTTCGTG	*Plasmodium* spp.
RNaSP F	TGTTTGCAGATTTGGACCTGC	Human RNase p
RNaSeP R	AATAGCCAAGGTGGAGCGGCT	Human RNase p
RNaseP P	TGCGCGGACTTGTGGA	Human RNase p
FAL F	ATTGCTTTTGAGAGGTTTTGTTACTT	*P. falciparum*
FAL R	GCTGAGTATTCAAACACAATGAACTCAA	*P. falciparum*
FAL P	CATAACAGACGGGTAGTCAT	*P. falciparum*
MAL F	GCATGGAATTTTGTTACTTTGA	*P. malariae*
MAL R	ATGCCTGTAGTATTCAACACAGAAAC	*P. malariae*
MAL P	TGTTCAAAGCAAACAGTTAAAACA	*P. malariae*
OVA F	TTTTGAAGAATACATTAGGATACAATTAATG	*P. ovale curtisi*
OVA R	CATGCTTCCTCTAAGAAGCTTTACAAT	*P. ovale*
OVA-V F	TTTTGAAGAATATATTAGGATACATTATAG	*P. ovale Wallikeri*

OVA-V R	CATCGTTCCTCTAAGAAGCTTTACAAT	*P. ovale Wallikeri*
OVA P	CCTTTTCCCTATTCTACTTAATTCGCAATTCATG	*P. ovale curtisi*
OVA-V P	CCTTTTCCCTACTTAATTCGCTATTCATTG	*P. ovale Wallikeri*

Results

A total of 741 samples comprising 437 (59·0%) were from the symptomatic group and 304 (41·0%) from the asymptomatic group were analyzed. The description of the study population is summarized in **Table 2**. Metadata were only available for a subset of individuals. Besides shared hospital catchment, the proportion of individuals per age band were mostly comparable among the symptomatic and asymptomatic groups.

Table 2: Metadata among symptomatic and asymptomatic malaria infections

	**Symptomatic (n=394)**	**Asymptomatic (n=132)**
**Age in years (n), mean, median, SD, IQR**	<5 (282), 3·09, 3, 1·46, (2-4)	<5 (87), 3·31, 3, 1·32, (2-4)
	5-17 (103), 8·49, 8, 2·83, (6-10)	5-17 (134), 9·53, 9, 2·62, (7-11)
	>18 (47), 28·15, 27, 9·72, (20-33)	>18 (82), 35·71, 34, 10·8, (28-42)
**Female %**	50·3	58·4
**Male %**	49·7	41·3

Plasmodium speciation by qPCR assay showed that, of the 741 infections 668 (90·15%), contained *P. falciparum*, 154 (20·78%) *P. ovalewallikeri*, 70 (9·45%), *P. malariae* and 64 (8·7%) *P. ovalecurtisi*. A total of 544 (73·41%) samples had single species while 197 (26·59%) had multiple species infections. *P. falciparum* was the most frequent single-species infection occurring in 480 (64.78%) infections, followed *by P. ovalewallikeri* in 33 (4·45%),

P. malariae 16 (2·15%) and *P. ovalecurtisi* 13 (2·02%). Notably, all the *P. malariae* and *P. o curtisi* single-species infections were all from asymptomatic individuals. (**Fig 2**).

Symptomatic infections

Plasmodium falciparum was the most prevalent species occurring in 421 (96·3%) of the 437 symptomatic infections followed by *P. ovalewallikeri* 123 (28·3%), and *P. ovalecurtisi* 40 (9·2%) while *P. malariae* was least at 30 (6·8%). *P. falciparum* 262 (60%) and *P. ovalewallikeri* 15(3·4%) were the only single species detected among the symptomatic infections. A total of 160 (36·61%) of the symptomatic individuals harbored multiple *Plasmodium* species comprising 2 species 142 (32·49 %), three species 17 (3·9%) **Fig 2**. Notably, of the 97 dual infections comprising of *P. falciparum* and *P. ovalewallikeri*, 92 (21·1%) of them were found in symptomatic individuals. Further, neither *P. ovalecurtisi* nor *P. malariae* occurred as a single species infection among symptomatic individuals.

Fig 2. *Plasmodium* species prevalence among symptomatic infections by molecular diagnosis: Symptomatic means infections that generate malarial parasitemia alongside symptoms.

Asymptomatic infections

Plasmodium falciparum was detected in 247 (81·25%) of the 304 asymptomatic infections as the most prevalent species followed by *P. malariae* 40 (13·2%), *P. ovalewallikeri* 31 (10·19%), and *P. ovalecurtisi* 26 (8·55%) respectively.267 (87·83%) of the samples were single species infections with *P. falciparum* 218 (71·7%), *P. ovalewallikeri*

18 (5·9%), *P. malariae* 16 (5·3%), and *P. ovalecurtisi* 15 (4·9%). The 46 (15·1%) multiple species infections comprised either two 34 (11·18%) or three 3 (0·98%) species infections (**Fig 3**).

Fig 3. *Plasmodium* species prevalence among asymptomatic infections by molecular diagnosis: Asymptomatic malaria parasite carriers are the infections that generate malarial parasitemia in the absence of fever or other classical symptoms and signs, in otherwise healthy individuals.

The *P. falciparum* single-species infections were more prevalent in asymptomatic than symptomatic individuals **(Figs 2 & 3**). The multiple species were more likely to be symptomatic infections than asymptomatic. Assessment of role of infection composition in disease presentation showed that *P. falciparum* with *P. ovalewallikeri* ; *P. falciparum* with *P. ovalecurtisi* and *P. falciparum P. ovalewallikeri* with *P. ovalecurtisi* mixed-species infections had higher risk of progressing to symptomatic infection than remaining asymptomatic (**Fig 4**). On the other hand, infections comprising single species infections *P. malariae* and *P. ovalecurtisi* or *P. malariae* with *P. ovalewallikeri* mixed species were more likely to be asymptomatic (**Fig 4**).

Figure 4: Risk of species composition in *Plasmodium* infection presentation

Discussion

This study concurrently compared the prevalence of *Plasmodium* species among symptomatic individuals seeking treatment at the Kombewa Sub-County hospital versus asymptomatic individuals in households within the same geographic location that doubles up as the catchment of the hospital using qPCR. About 13% and 26% prevalence of multiple species was detected among asymptomatic and symptomatic infections, respectively. Studies in west and central Africa focusing on school-going children have also shown higher burden of non-falciparum species in asymptomatic malaria parasite carriers (35, 36). This finding is contrary to a recent meta-analysis showing no difference in the prevalence of either *P. malariae* or *P. ovale spp* between symptomatic and asymptomatic individuals (71). Additionally, large scale studies of non-falciparum parasites report conflicting results for those from west Africa (72) versus those from central or East Africa (73). These varying observations underscoring the peculiarity of *Plasmodium* infection presentation across different disease endemicities needing characterizing of diverse disease ecotypes for effective targeting.

Occurrence of *Plasmodium* species parasitemia in absence of classical symptoms of malaria or determinants of onset of symptoms is an unresolved debate. A prospective study by Chen and coworkers showed that some individuals harboring *Plasmodium* parasites can either remain healthy for an unlimited amount of time and referred to as asymptomatic (30) while others progress to disease with malaria symptoms within 7-85 days and seek for the treatment depending on various factors including the species composition of the infection (15-17, 32).Studies on inter-parasite interactions within human hosts suggest that parasites

often interact with unrelated competitors within the host (74) and use this information to prioritize the resource allocation framework opting to increase replication to occupy most of the red blood cells at the expense of the development of sexual stages as one of the exclusion strategies. The changes in resource utilization resulting from the presence of the additional species have been associated with modulation of the host response (43, 75-77) toward either remaining in an asymptomatic state of the infection (78) or transitioning to symptomatic for virulence enhancing species mixtures (79). Detection of higher frequency of multiple species infections among the symptomatic than asymptomatic with a reciprocal higher frequency of single species infections in asymptomatic than symptomatic by this study appear to suggest that infection with more than one species is likely to progress to a more symptomatic state than single-species infections as reported elsewhere (54, 55). It was however surprising that asymptomatic individuals harbored more diverse species profiles than symptomatic based on PCR-based diagnosis.

The composition of mixed species infections varied significantly between the symptomatic and asymptomatic infection. There was a ten-fold higher prevalence of co-infections containing *P. falciparum* and *P. ovalewallikeri* among the symptomatic than asymptomatic. A similar pattern of frequency was observed among infections containing *P. falciparum, P. ovalewallikeri,* plus *P. ovalecurtisi,* contrary to *P. falciparum/P. ovalecurtisi, or P. falciparum/P. malariae* or either of these species in single-species infections suggesting between species interaction. Parasite biology studies show that *P. falciparum* attacks all ages of red blood cells hence regarded as a generalist while *P. ovale* and *P. malariae* attack younger and old red blood cells respectively and are referred to as specialists (78-80). Studies show that in mixed-species infections, in the presence of an RBC generalist enhances the

density of RBC specialists.(77) A study monitoring blood levels of tumor necrosis factor-alpha (TNF-alpha), lactate dehydrogenase (LDH), Thrombin-antithrombin III (TAT) and elastase as drivers of malaria symptoms through modulation of host immune response among uncomplicated malaria. The study further noted that these parameters were higher per parasitized *P. ovale* erythrocyte levels than *P. falciparum*, with the former attaining full-strength host response at lower parasitemia than the latter (79). Observation of significantly higher frequency *P. falciparum* and *P. ovalewallikeri* among naturally acquired symptomatic than asymptomatic of infection by this study appears to suggest that parasitemia increases in *P. ovalewallikeri*, a specialist, given the presence of *P. falciparum* a generalist, could be contributing to elevated immune response leading to the occurrence of the symptoms among these infections, emphasizing the differences in the biology of the two ovale species. Notably, *P. ovalewallikeri* single-species infections were detected among symptomatic as well as asymptomatic infections while *P. ovalecurtisi* alone was not detected among the symptomatic, suggesting that *P. ovalewallikeri* alone causes symptoms more frequently than *P. ovalecurtisi*.

Single-species infections of *P. falciparum, P. malariae, or P. ovalecurtisi* were mostly detected among asymptomatic individuals than symptomatic. Notably, *P. ovalecurtisi* single-species infections were detected in 4·8% of the asymptomatic infections while none was detected in symptomatic infections. Additionally, infections comprising *P. malariae*/*P. ovalecurtisi* or *P. malariae*/*P. ovalewallikeri* were detected in asymptomatic infections at less than 2% frequency while none were detected among symptomatic infections. *P. malariae* co-infections with *P. ovalecurtisi* or *P. ovalewallikeri* have been associated with low pyrogenic thresholds, occurring in less than 2% of the symptomatic cases (78). This infection

characteristic is a challenge for malaria elimination since the chronically infected individual would remain obscure to healthcare facilities while still capable of transmitting the parasite to mosquitoes and therefore sustaining transmission to humans. Such infections have been proposed as the last battlefield for malaria elimination (81) since they will also remain undetected by the histidine-rich protein based malaria rapid diagnostic test that are expedient for population-based strategies of bringing down diseases.

The study depended on the vital signs of the participants at the time of enrolment such that those with a high temperature of 38°C and above, muscle pains and generally feeling un-well often characterized by recurrent fever and chills were symptomatic while those without these signs were grouped as symptomatic. It was difficult to ascertain when both asymptomatic and symptomatic acquired infections before the enrolment date for the study to understand whether the asymptomatic were destined to be symptomatic since the infections had not attained the pyrogenic threshold or not owing to the design of the study. It was again not possible to ascertain which of the infecting species in the infection came first and which one came later to tell whether there was protectiveness proffered by some species or immune silencing by other species to delay symptoms. Finally, host immunity was not characterized were not the endpoint of this study.

Conclusion

These findings in a malaria-endemic zone suggest that intervention strategies need to consider the species composition of asymptomatic malaria infections to progress the region to the elimination phase. The ten-fold higher frequency of *P. ovalewallikeri* species among the symptomatic than asymptomatic infections and higher *P. falciparum* single-species

infections in the asymptomatic than symptomatic appears to associate this infection composition with higher likelihood of symptoms. Interventions need to focus on the mixed infections asymptomatic malaria parasite carriers since these two serve as silent reservoirs for transmission and are a lynchpin to malaria elimination in a given region.

Acknowledgments

Material has been reviewed by the Walter Reed Army Institute of Research. There is no objection to its presentation and/or publication. The opinions or assertions contained herein are the private views of the author, and are not to be construed as official, or as reflecting true views of the Department of the Army or the Department of Defense. The investigators have adhered to the policies for protection of human subjects as prescribed in AR 70–25. We thank the individuals who participated in this study for providing this invaluable samples and time to generate these findings. We also thank the county Government of Kisumu and The Ministry of Health Kenya for giving us access to these samples. We thank the Commander, Walter Reed Army Institute of Research_Africa, COL. Lacy Shannon, Director Walter Reed Army Institute of Research -Africa Kisumu field Stations, COL. Gerald Keller, Deputy Director General, Center for Clinical Research, Kenya Medical Research Institute, Kenya for supporting this study and giving their permission to publish these data. We also thank all clinical staff at all the participating hospitals for their assistance.

References

1. Ansari HR, Templeton TJ, Subudhi AK, Ramaprasad A, Tang J, Lu F, et al. Genome-scale comparison of expanded gene families in Plasmodium ovale wallikeri and Plasmodium ovale curtisi with Plasmodium malariae and with other Plasmodium species. International journal for parasitology. 2016;46(11):685-96.
2. Antinori S, Galimberti L, Milazzo L, Corbellino M. Plasmodium knowlesi: the emerging zoonotic malaria parasite. Acta tropica. 2013;125(2):191-201.
3. Snounou G, Sharp PM, Culleton R. The two parasite species formerly known as Plasmodium ovale. Trends in parasitology. 2024;40(1):21-7.
4. Bhatt S, Weiss D, Cameron E, Bisanzio D, Mappin B, Dalrymple U, et al. The effect of malaria control on Plasmodium falciparum in Africa between 2000 and 2015. Nature. 2015;526(7572):207-11.
5. Sinka ME, Bangs MJ, Manguin S, Rubio-Palis Y, Chareonviriyaphap T, Coetzee M, et al. A global map of dominant malaria vectors. Parasites & vectors. 2012;5(1):1-11.
6. Weiss DJ, Lucas TC, Nguyen M, Nandi AK, Bisanzio D, Battle KE, et al. Mapping the global prevalence, incidence, and mortality of Plasmodium falciparum, 2000–17: a spatial and temporal modelling study. The Lancet. 2019;394(10195):322-31.
7. Battle KE, Lucas TC, Nguyen M, Howes RE, Nandi AK, Twohig KA, et al. Mapping the global endemicity and clinical burden of Plasmodium vivax, 2000–17: a spatial and temporal modelling study. The Lancet. 2019;394(10195):332-43.
8. Price RN, Commons RJ, Battle KE, Thriemer K, Mendis K. Plasmodium vivax in the Era of the Shrinking P. falciparum Map. Trends in parasitology. 2020;36(6):560-70.
9. Mahittikorn A, Masangkay FR, Kotepui KU, Milanez GDJ, Kotepui M. Comparison of Plasmodium ovale curtisi and Plasmodium ovale wallikeri infections by a meta-analysis approach. Scientific reports. 2021;11(1):1-15.
10. Sutherland CJ. Persistent parasitism: the adaptive biology of malariae and ovale malaria. Trends in Parasitology. 2016;32(10):808-19.
11. Zimmerman PA, Mehlotra RK, Kasehagen LJ, Kazura JW. Why do we need to know more about mixed Plasmodium species infections in humans? Trends in parasitology. 2004;20(9):440-7.
12. Portugal S, Drakesmith H, Mota MM. Superinfection in malaria: Plasmodium shows its iron will. EMBO reports. 2011;12(12):1233-42.
13. Imwong M, Nakeesathit S, Day NP, White NJ. A review of mixed malaria species infections in anopheline mosquitoes. Malaria journal. 2011;10(1):1-12.
14. Beier MS, Schwartz IK, Beier JC, Perkins PV, Onyango F, Koros JK, et al. Identification of malaria species by ELISA in sporozoite and oocyst infected Anopheles from western Kenya. The American journal of tropical medicine and hygiene. 1988;39(4):323-7.
15. Bruce MC, Donnelly CA, Alpers MP, Galinski MR, Barnwell JW, Walliker D, et al. Cross-species interactions between malaria parasites in humans. Science. 2000;287(5454):845-8.
16. Bruce MC, Day KP. Cross-species regulation of Plasmodium parasitemia in semi-immune children from Papua New Guinea. Trends in parasitology. 2003;19(6):271-7.
17. Richie T. Interactions between malaria parasites infecting the same vertebrate host. Parasitology. 1988;96(3):607-39.
18. Collins WE, Jeffery GM. Plasmodium malariae: parasite and disease. Clinical microbiology reviews. 2007;20(4):579-92.

19. Cordina CJ, Culleton R, Jones BL, Smith CC, MacConnachie AA, Coyne MJ, et al. Plasmodium knowlesi: clinical presentation and laboratory diagnosis of the first human case in a Scottish traveler. Journal of travel medicine. 2014;21(5):357-60.
20. Groger M, Fischer HS, Veletzky L, Lalremruata A, Ramharter M. A systematic review of the clinical presentation, treatment and relapse characteristics of human Plasmodium ovale malaria. Malaria journal. 2017;16(1):1-16.
21. Verra F, Angheben A, Martello E, Giorli G, Perandin F, Bisoffi Z. A systematic review of transfusion-transmitted malaria in non-endemic areas. Malaria Journal. 2018;17:1-14.
22. Orish V, Afutu L, Ayodele O, Likaj L, Marinkovic A, Sanyaolu A, editors. A 4-day incubation period of Plasmodium falciparum infection in a nonimmune patient in Ghana: a case report. Open Forum Infectious Diseases; 2019: Oxford University Press US.
23. Okafor CN, Finnigan NA. Plasmodium ovale malaria. 2018.
24. Bartoloni A, Zammarchi L. Clinical aspects of uncomplicated and severe malaria. Mediterranean journal of hematology and infectious diseases. 2012;4(1).
25. Bartoloni A, Zammarchi L. Clinical aspects of uncomplicated and severe malaria. Mediterranean journal of hematology and infectious diseases. 2012;4(1):e2012026.
26. Krajden S, Panisko DM, Tobe B, Yang J, Keystone JS. Prolonged infection with Plasmodium falciparum in a semi-immune patient. Trans R Soc Trop Med Hyg. 1991;85(6):731-2.
27. Greenwood T, Vikerfors T, Sjöberg M, Skeppner G, Färnert A. Febrile Plasmodium falciparum malaria 4 years after exposure in a man with sickle cell disease. Clinical Infectious Diseases. 2008;47(4):e39-e41.
28. Szmitko PE, Kohn ML, Simor AE. Plasmodium falciparum malaria occurring 8 years after leaving an endemic area. Diagnostic microbiology and infectious disease. 2009;63(1):105-7.
29. Hamainza B, Moonga H, Sikaala CH, Kamuliwo M, Bennett A, Eisele TP, et al. Monitoring, characterization and control of chronic, symptomatic malaria infections in rural Zambia through monthly household visits by paid community health workers. Malaria journal. 2014;13(1):1-19.
30. Chen I, Clarke SE, Gosling R, Hamainza B, Killeen G, Magill A, et al. "Asymptomatic" malaria: a chronic and debilitating infection that should be treated. PLoS medicine. 2016;13(1):e1001942.
31. Moonen B, Cohen JM, Snow RW, Slutsker L, Drakeley C, Smith DL, et al. Operational strategies to achieve and maintain malaria elimination. The Lancet. 2010;376(9752):1592-603.
32. Lindblade KA, Steinhardt L, Samuels A, Kachur SP, Slutsker L. The silent threat: asymptomatic parasitemia and malaria transmission. Expert review of anti-infective therapy. 2013;11(6):623-39.
33. Bottius E, Guanzirolli A, Trape J-F, Rogier C, Konate L, Druilhe P. Malaria: even more chronic in nature than previously thought; evidence for subpatent parasitaemia detectable by the polymerase chain reaction. Transactions of the Royal Society of Tropical Medicine and Hygiene. 1996;90(1):15-9.
34. Rouamba T, Samadoulougou S, Ouédraogo M, Hien H, Tinto H, Kirakoya-Samadoulougou F. Asymptomatic malaria and anaemia among pregnant women during high and low malaria transmission seasons in Burkina Faso: household-based cross-sectional surveys in Burkina Faso, 2013 and 2017. Malaria journal. 2021;20(1):1-13.
35. Abdulraheem MA, Ernest M, Ugwuanyi I, Abkallo HM, Nishikawa S, Adeleke M, et al. High prevalence of Plasmodium malariae and Plasmodium ovale in co-infections with Plasmodium falciparum in asymptomatic malaria parasite carriers in southwestern Nigeria. International journal for parasitology. 2022;52(1):23-33.

36. Nundu SS, Culleton R, Simpson SV, Arima H, Muyembe J-J, Mita T, et al. Malaria parasite species composition of Plasmodium infections among asymptomatic and symptomatic school-age children in rural and urban areas of Kinshasa, Democratic Republic of Congo. Malaria journal. 2021;20:1-13.
37. Kho S, Qotrunnada L, Leonardo L, Andries B, Wardani PA, Fricot A, et al. Evaluation of splenic accumulation and colocalization of immature reticulocytes and Plasmodium vivax in asymptomatic malaria: A prospective human splenectomy study. PLoS medicine. 2021;18(5):e1003632.
38. Pava Z, Handayuni I, Trianty L, Utami RA, Tirta YK, Puspitasari AM, et al. Passively versus actively detected malaria: similar genetic diversity but different complexity of infection. The American journal of tropical medicine and hygiene. 2017;97(6):1788-96.
39. Buchwald AG, Sorkin JD, Sixpence A, Chimenya M, Damson M, Wilson ML, et al. Association between age and Plasmodium falciparum infection dynamics. American journal of epidemiology. 2019;188(1):169-76.
40. Worku L, Damtie D, Endris M, Getie S, Aemero M. Asymptomatic malaria and associated risk factors among school children in Sanja town, Northwest Ethiopia. International scholarly research notices. 2014;2014.
41. Kwiatkowski D, Nowak M. Periodic and chaotic host-parasite interactions in human malaria. Proceedings of the National Academy of Sciences. 1991;88(12):5111-3.
42. Mason DP, McKENZIE FE. Blood-stage dynamics and clinical implications of mixed Plasmodium vivax–Plasmodium falciparum infections. The American journal of tropical medicine and hygiene. 1999;61(3):367.
43. Bruce MC, Day KP. Cross-species regulation of malaria parasitaemia in the human host. Current opinion in microbiology. 2002;5(4):431-7.
44. Zarocostas J. Malaria treatment should begin with parasitological diagnosis where possible, says WHO. BMJ: British Medical Journal (Online). 2010;340.
45. Newman RD. World Malaria Report 2011. 2012.
46. Musuva A, Ejersa W, Kiptui R, Memusi D, Abwao E. The malaria testing and treatment landscape in Kenya: results from a nationally representative survey among the public and private sector in 2016. Malaria journal. 2017;16(1):1-13.
47. Kamal-Yanni MM, Potet J, Saunders PM. Scaling-up malaria treatment: a review of the performance of different providers. Malaria journal. 2012;11(1):1-10.
48. Alonso P, Noor AM. The global fight against malaria is at crossroads. The Lancet. 2017;390(10112):2532-4.
49. Larsen DA, Ngwenya-Kangombe T, Cheelo S, Hamainza B, Miller J, Winters A, et al. Location, location, location: environmental factors better predict malaria-positive individuals during reactive case detection than index case demographics in Southern Province, Zambia. Malaria journal. 2017;16(1):1-9.
50. Mueller I, Slutsker L, Tanner M. Estimating the burden of malaria: the need for improved surveillance. PLoS medicine. 2011;8(12):e1001144.
51. Kamau E, Alemayehu S, Feghali KC, Saunders D, Ockenhouse CF. Multiplex qPCR for detection and absolute quantification of malaria. PloS one. 2013;8(8):e71539.
52. Veron V, Simon S, Carme B. Multiplex real-time PCR detection of P. falciparum, P. vivax and P. malariae in human blood samples. Experimental parasitology. 2009;121(4):346-51.
53. Murray CK, Gasser Jr RA, Magill AJ, Miller RS. Update on rapid diagnostic testing for malaria. Clinical microbiology reviews. 2008;21(1):97-110.

54. Okyere B, Owusu-Ofori A, Ansong D, Buxton R, Benson S, Osei-Akoto A, et al. Point prevalence of asymptomatic Plasmodium infection and the comparison of microscopy, rapid diagnostic test and nested PCR for the diagnosis of asymptomatic malaria among children under 5 years in Ghana. PloS one. 2020;15(7):e0232874.
55. van Eijk AM, Sutton PL, Ramanathapuram L, Sullivan SA, Kanagaraj D, Priya GSL, et al. The burden of submicroscopic and asymptomatic malaria in India revealed from epidemiology studies at three varied transmission sites in India. Scientific reports. 2019;9(1):1-11.
56. Perandin F, Manca N, Calderaro A, Piccolo G, Galati L, Ricci L, et al. Development of a real-time PCR assay for detection of Plasmodium falciparum, Plasmodium vivax, and Plasmodium ovale for routine clinical diagnosis. Journal of clinical microbiology. 2004;42(3):1214-9.
57. Kamau E, Tolbert LS, Kortepeter L, Pratt M, Nyakoe N, Muringo L, et al. Development of a highly sensitive genus-specific quantitative reverse transcriptase real-time PCR assay for detection and quantitation of Plasmodium by amplifying RNA and DNA of the 18S rRNA genes. Journal of clinical microbiology. 2011;49(8):2946-53.
58. Ouédraogo AL, Eckhoff PA, Luty AJ, Roeffen W, Sauerwein RW, Bousema T, et al. Modeling the impact of Plasmodium falciparum sexual stage immunity on the composition and dynamics of the human infectious reservoir for malaria in natural settings. PLoS pathogens. 2018;14(5):e1007034.
59. Gholizadeh S, Karimi NN, Zakeri S, Djadid ND. The role of molecular techniques on malaria control and elimination programs in iran: A review article. Iranian journal of parasitology. 2018;13(2):161.
60. Tedla M. A focus on improving molecular diagnostic approaches to malaria control and elimination in low transmission settings. Parasite epidemiology and control. 2019;6:e00107.
61. Njuguna P, Maitland K, Nyaguara A, Mwanga D, Mogeni P, Mturi N, et al. Observational study: 27 years of severe malaria surveillance in Kilifi, Kenya. BMC medicine. 2019;17(1):1-9.
62. Cheaveau J, Mogollon DC, Mohon MAN, Golassa L, Yewhalaw D, Pillai DR. Asymptomatic malaria in the clinical and public health context. Expert review of anti-infective therapy. 2019;17(12):997-1010.
63. Mayxay M, Pukrittayakamee S, Newton PN, White NJ. Mixed-species malaria infections in humans. Trends in parasitology. 2004;20(5):233-40.
64. Kotepui M, Kotepui KU, Milanez GDJ, Masangkay FR. Plasmodium spp. mixed infection leading to severe malaria: A systematic review and meta-analysis. Scientific Reports. 2020;10(1):1-12.
65. Akala HM, Watson OJ, Mitei KK, Juma DW, Verity R, Ingasia LA, et al. Plasmodium interspecies interactions during a period of increasing prevalence of Plasmodium ovale in symptomatic individuals seeking treatment: an observational study. The Lancet Microbe. 2021;2(4):e141-e50.
66. Achieng AO, Muiruri P, Ingasia LA, Opot BH, Juma DW, Yeda R, et al. Temporal trends in prevalence of Plasmodium falciparum molecular markers selected for by artemether–lumefantrine treatment in pre-ACT and post-ACT parasites in western Kenya. International Journal for Parasitology: Drugs and Drug Resistance. 2015;5(3):92-9.
67. Akala HM, Eyase FL, Cheruiyot AC, Omondi AA, Ogutu BR, Waters NC, et al. Antimalarial drug sensitivity profile of western Kenya Plasmodium falciparum field isolates determined by a SYBR Green I in vitro assay and molecular analysis. The American journal of tropical medicine and hygiene. 2011;85(1):34.

68. Rougemont M, Van Saanen M, Sahli R, Hinrikson HP, Bille J, Jaton K. Detection of four Plasmodium species in blood from humans by 18S rRNA gene subunit-based and species-specific real-time PCR assays. Journal of clinical microbiology. 2004;42(12):5636-43.
69. Kamau E, Alemayehu S, Feghali KC, Juma DW, Blackstone GM, Marion WR, et al. Sample-ready multiplex qPCR assay for detection of malaria. Malaria journal. 2014;13(1):1-8.
70. Calderaro A, Piccolo G, Gorrini C, Montecchini S, Rossi S, Medici MC, et al. A new real-time PCR for the detection of Plasmodium ovale wallikeri. PloS one. 2012;7(10):e48033.
71. Hawadak J, Nana RRD, Singh V. Global trend of Plasmodium malariae and Plasmodium ovale spp. malaria infections in the last two decades (2000–2020): a systematic review and meta-analysis. Parasites & vectors. 2021;14(1):1-14.
72. Chen J, Ma X, Tang J, Xu S, Gu Y, Tang F, et al. Disparate selection of mutations in the dihydrofolate reductase gene (dhfr) of Plasmodium ovale curtisi and P. o. wallikeri in Africa. PLOS Neglected Tropical Diseases. 2022;16(12):e0010977.
73. Joste V, Coppée R, Bailly J, Rakotoarivony Y, Tchokoteu FGT, Achache S, et al. Plasmodium ovale spp dhfr mutations associated with reduced susceptibility to pyrimethamine in sub-Saharan Africa: a retrospective genetic epidemiology and functional study. The Lancet Microbe. 2024;5(7):669-78.
74. Reece SE, Thompson J. Transformation of the rodent malaria parasite Plasmodium chabaudi and generation of a stable fluorescent line PcGFP CON. Malaria journal. 2008;7(1):1-6.
75. Mideo N. Parasite adaptations to within-host competition. Trends Parasitol. 2009;25(6):261-8.
76. Costa G, Gildenhard M, Eldering M, Lindquist RL, Hauser AE, Sauerwein R, et al. Non-competitive resource exploitation within mosquito shapes within-host malaria infectivity and virulence. Nat Commun. 2018;9(1):3474.
77. Ramiro RS, Pollitt LC, Mideo N, Reece SE. Facilitation through altered resource availability in a mixed-species rodent malaria infection. Ecology letters. 2016;19(9):1041-50.
78. Faye FB, Spiegel A, Tall A, Sokhna C, Fontenille D, Rogier C, et al. Diagnostic criteria and risk factors for Plasmodium ovale malaria. The Journal of infectious diseases. 2002;186(5):690-5.
79. Hemmer CJ, Holst FGE, Kern P, Chiwakata CB, Dietrich M, Reisinger EC. Stronger host response per parasitized erythrocyte in Plasmodium vivax or ovale than in Plasmodium falciparum malaria. Tropical Medicine & International Health. 2006;11(6):817-23.
80. Okafor CN, Finnigan NA. Plasmodium ovale malaria. StatPearls [Internet]: StatPearls Publishing; 2020.
81. Nyarko PB, Claessens A. Understanding host–pathogen–vector interactions with chronic asymptomatic malaria infections. Trends in Parasitology. 2020.

Supporting Information

S1. Table 1: Primers and probes used for screening and identification of *Plasmodium* species

S2. Table 2: Metadata among symptomatic and asymptomatic malaria infections

S3. Fig 1: Map showing Kombewa region where Kombewa Sub County hospital is located.

S4. Fig 2: *Plasmodium* species prevalence among symptomatic infections by molecular diagnosis: Symptomatic means infections that generate malarial parasitemia alongside symptoms.

S5. Fig 3: *Plasmodium* species prevalence among asymptomatic infections by molecular diagnosis: Asymptomatic malaria parasite carriers are the infections that generate malarial parasitemia in the absence of fever or other classical symptoms and signs, in otherwise healthy individuals.

S6. Fig 4: Risk of species composition in *Plasmodium* infection presentation

Figure

Click here to access/download;Figure;Figure 1_Map of Kenya_Kombewa.docx

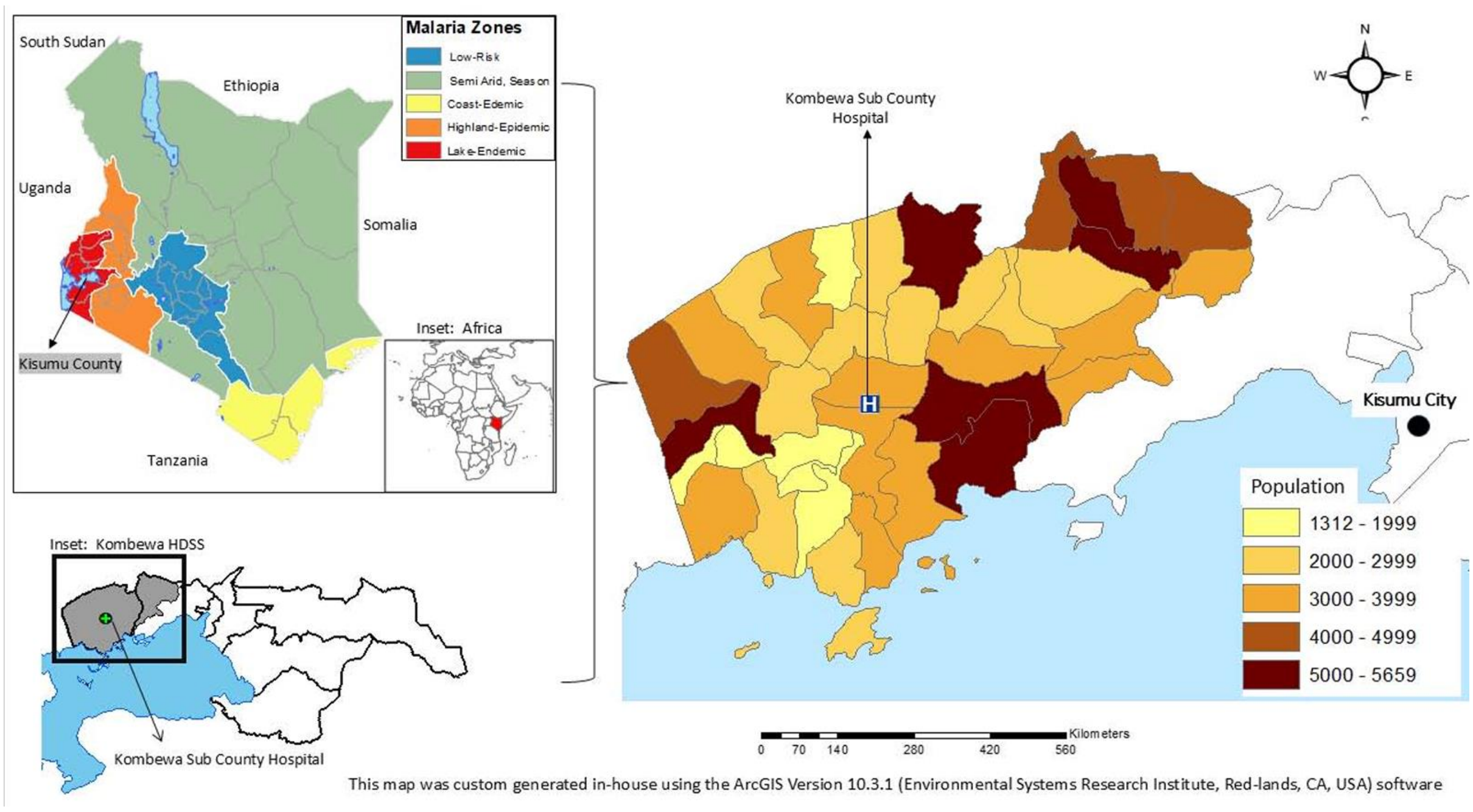

Fig 1: Map showing Kombewa region where Kombewa Sub County hospital is located.

Click here to access/download;Figure;Figure 2_Symptomatic infections.docx

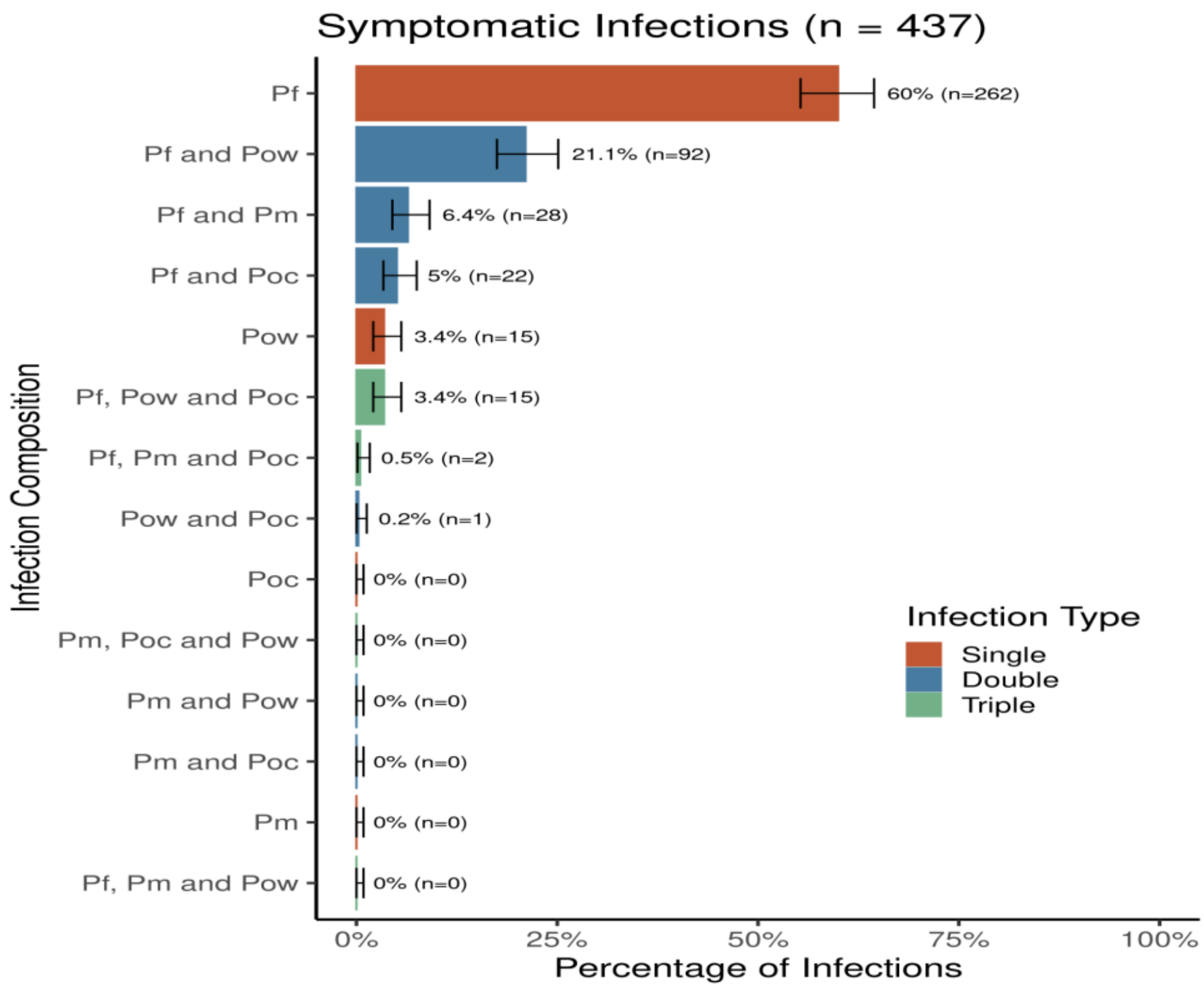

Fig 2: *Plasmodium* mixed and single species frequency among symptomatic infections by molecular diagnosis: Symptomatic infections refer to infections that generate malarial parasitemia alongside symptoms.

Figure

Click here to access/download;Figure;Figure 3_Asymptomatic infections.docx

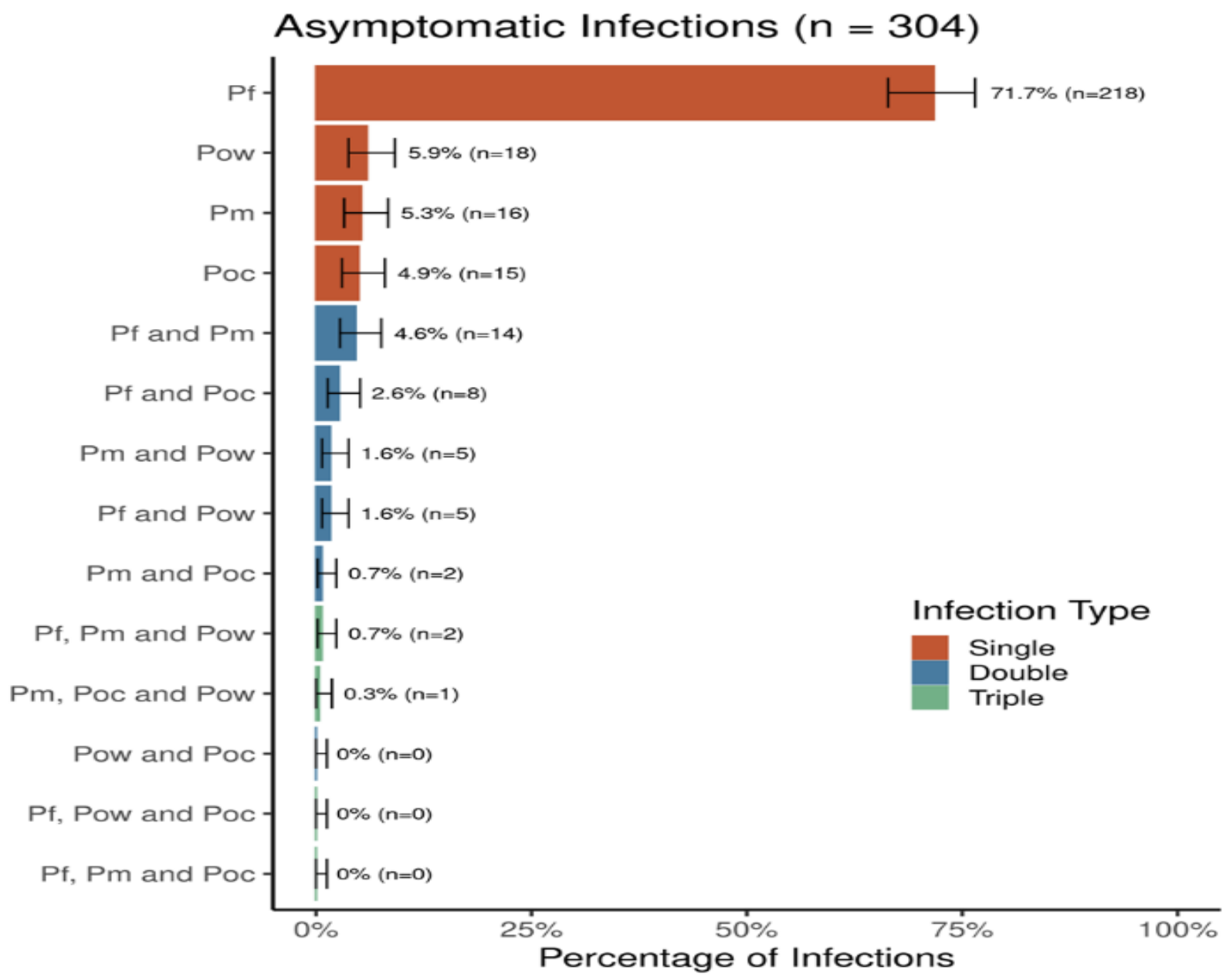

Fig 3: *Plasmodium* mixed and single species frequency among asymptomatic infections by molecular diagnosis: Asymptomatic malaria parasite carriers are the infections that generate malarial parasitemia in the absence of fever or other classical symptoms and signs, in otherwise healthy individuals.

Figure
Click here to access/download;Figure;Figure 4_Risk of species composition.docx

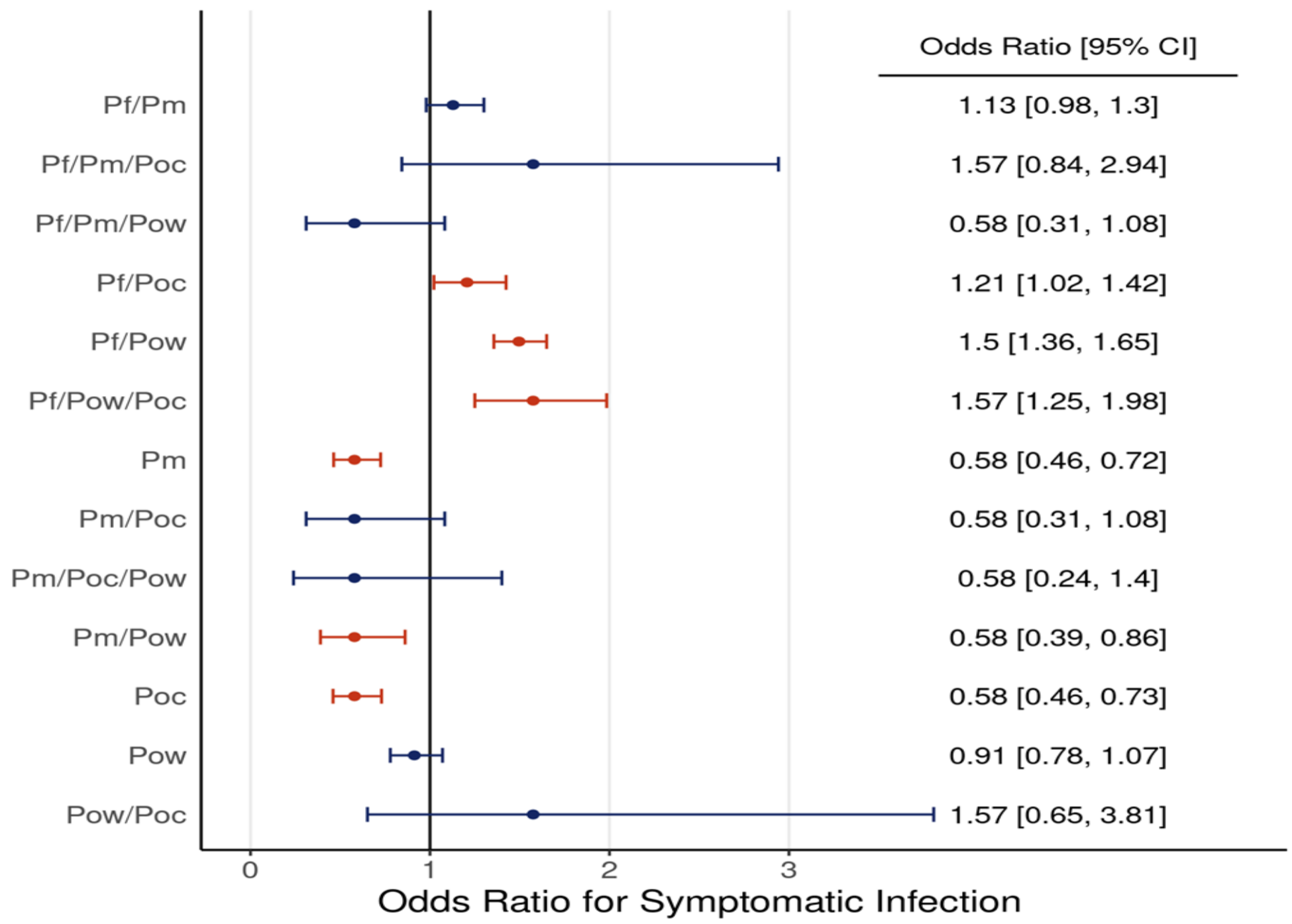

Fig 4: Risk of species composition in *Plasmodium* infection presentation.

Click here to access/download
Supporting Information
WRAIR# 1384 CRR Approval_Study 1.pdf

Click here to access/download
Supporting Information
WRAIR# 2064 CRR Approval_Study 2.pdf
