# Supplementary material for: High prevalence of Plasmodium ovalewallikeri in co-infections with Plasmodium falciparum in symptomatic than asymptomatic Plasmodium parasite infections in Kisumu, Kenya
Source: PLoS One. 2026 Oct 7;21(10):e0334188. doi: 10.1371/journal.pone.0334188 (PMC13645224; doi:10.1371/journal.pone.0334188)
Supplement: Supplementary file 2 [file pone.0334188.s005.pdf]

PLOS One

High prevalence of Plasmodium ovalewallikeri in co-infections with Plasmodium falciparum in symptomatic than asymptomatic Plasmodium parasite infections in Kisumu, Kenya

--Manuscript Draft--

Manuscript Number: PONE-D-25-45620R1

Article Type: Research Article

Full Title: High prevalence of Plasmodium ovalewallikeri in co-infections with Plasmodium falciparum in symptomatic than asymptomatic Plasmodium parasite infections in Kisumu, Kenya

Short Title: Varying burden of Plamodium ovale and Plasmodium malariae based on infection presentation

Corresponding Author: Hoseah M. Akala, PhD
Kenya Medical Research Institute
Kisumu, Kenya, KENYA

Keywords: Plasmodium species; symptomatic; Asymptomatic

Abstract:

Plasmodium falciparum (Pf), Plasmodium ovalewallikeri (Pow), Plasmodium ovalecurtisi (Poc), and Plasmodium malariae (Pm) coexists in Kenya sympatrically. Some infections progress to classical symptoms of malaria prompting hospital visits, while others remain asymptomatic. There is limited information on whether the species composition status of infections in endemic areas influences progression to either asymptomatic or symptomatic infection. This cross-sectional study determined the composition of Plasmodium species among symptomatic versus asymptomatic participants in Kombewa, Kenya.

Between 2015 and 2016, samples from 741 individuals, comprising of 437 from symptomatic at Kombewa Subcounty hospital and 304 from asymptomatic participants from within Kombewa Subcounty hospital's catchment area were analyzed for Plasmodium species composition using real-time qPCR assay targeting the small subunit ribosomal RNA (ssrRNA) gene. R Studio was used to describe Plasmodium species prevalence, and the odds of occurring within given infection presentation.

The 741 samples predominantly contained Pf 655/741 (88.3%) followed by Pow 153/741 (20.6%), Pm 70/741 (9.4%) and Poc 64/741 (8.6%); 12/741 (1.62%) did not amplify any species. A total of 535/741 (72.2%) contained single species, and 194/741 (26.18%) multiple species. Species distribution differed significantly, with Pf and Pow more prevalent in symptomatic cases, while Pm and Poc were more common in asymptomatic individuals (p=0.000). Pm and Poc single-species infections occurred among asymptomatic infections only. There were significantly higher odds of multiple species occurrence for infections containing Pf/Pow 92 (95.8%) among symptomatic than asymptomatic infections (OR 1.5, 95% CI [1.36-1.65], p = 0.000). Overall, mixed-species infections involving Pf and Pow were significantly more common in symptomatic individuals, posing a risk of false diagnosis by Pf-Histidine-Rich Protein 2 test. This underscores the need for deployment of interventions that target all Plasmodium species. These findings on burden across symptomatic vs asymptomatic infections suggest need for determination of the role of species composition in disease progression.

Order of Authors:

Hoseah M. Akala, PhD

Jackline A. Juma, BSc

Oliver J. Watson, PhD

Dennis Juma, MSc

Benjamin H. Opot, BSc

Gladys Chemwor, MSc

Mary Mutahi, BSc

Raphael Okoth, BSc

Edwin W. Mwakio, BSc

Powered by Editorial Manager® and ProduXion Manager® from Aries Systems Corporation

	Redemptah Yeda, MSc
	Charles O. Okello, BSc
	Agnes Cheruiyot, MSc
	Duke Omariba, BSc
	Iiliana Dayo, MSc
	Hellen A. Ogollah, BSc
	Gladys Biwott, MSc
	Charles Adega, BSc
	Fredrick Oluoch, MBChB
	Vincent Omondi, MBChB
	George Otieno, BSc
	Edwin Kamau, PhD
	Ben Andagalu, MSc
	Peter Sifuna, MSc
	Amanda Roth, PhD
	Sammy Khagayi, PhD
	Erick C. Grages, MBChB
	Bernherds Ogutu, PhD
**Response to Reviewers:**	Manuscript Review: High prevalence of Plasmodium ovalewallikeri in co-infections with Plasmodium falciparum in symptomatic than asymptomatic malaria parasite carriers in Kisumu, Kenya<br>We wish to convey our appreciation for the critical review and insightful comments from the reviewer. We have addressed all the queries that have made the manuscript better with more clarity and focus. Please see below the responses to the comments:<br><br>•The study is fairly well executed and presents results of original research EXCEPT these results have not been explicitly reflected and hence requires some major revision to improve the statistical meaning and clarity.<br>Response: Descriptive statistics were employed to illustrate the burden of Plasmodium species among the symptomatic and asymptomatic groups with positive infections. For inferential statistics, proportions were compared using the Chi-square test as appropriate for the categorical data including the stratified age bands, gender and Plasmodium species proportions. Continuous variables, including age in years, were first assessed for normality using the Shapiro–Wilk test and then expressed as median together with their corresponding interquartile ranges (IQR) when non-normal distributions were observed. Wilcoxon rank-sum test was used for non-parametric continuous variables specifically for age in years. The geometric mean was used as a measure of central tendency for parasite density. Logistic regression analysis was also conducted to assess the varying odds of Plasmodium species composition. All analyses were performed using R Studio version 4.5.1. Statistical analyses were performed at the 5% significance level and the corresponding 95% confidence interval (CI). The critical significance level was set at $p \leq 0.05$.<br>A detailed description on data management and statistical analysis is indicated on the manuscript line 208-222 and the results section has been revised to include the inferential and descriptive statistical analyses used.<br><br><br>•The results depict malaria co-infections as detected by molecular analysis which is robust but the presentation of the results does not explicitly reflect this which is misleading.<br>Response: The molecular analysis results for Plasmodium species that were obtained following use of qPCR are presented as percentage frequencies in table 3 and in figures 2-3.

In addition, the results section highlights the finding of the molecular analysis using qPCR which is mentioned on line 247-283. …" On this basis, Plasmodium species detection by qPCR assay showed that, 655/741 (88.3%) of the infections contained P. falciparum, 153/741 (20.7%) P. ovalewallikeri, 70/741 (9.4%) P. malariae, and 64/741 (8.7%) P. ovalecurtisi without consideration of the number of species per infection (Table 3). However, 12/741 (1.6%) amplifying for presence of Plasmodium genus did not amplify for any species…" The proportions of the Plasmodium species composition following qPCR detection assays have been presented as percentage frequencies in the results and discussion sections.

•No information has been provided on the statistical analysis used to analyze the data. I have highlighted the discrepancies in the reviewed copy of the manuscript as well. For instance, this presentation "Broadly, 741 mostly contained Pf 668 (90·15%) followed by Pow 154 (20·78%), Pm 7041 (9·45%) and Poc 64 (8·7%)''. There is need to explicitly indicate that this was a co-infection analysis, without which the information is misleading as the proportions look like mere percentages of 741.
Response: As stated above descriptive and inferential statistics were employed to illustrate the burden of Plasmodium species among the participants with positive infections as indicated in the manuscript, lines 215-222. Furthermore, we have indicated that Plasmodium co-infection analyses were conducted to detect the presence of single or multiple species and the raw data presented as percentage frequencies, lines 241-263.

•The wording reflecting the percentages and totals are not explicit. For instance, "The frequency of Plasmodium species among the symptomatic and asymptomatic infections were Pf 421 (96·3%) and 247 (81·25%), Pow 123 (28·3%) and 31 (10·19%), Poc 40 (9·2%) and 26 (8·55%) while Pm was 30(6·8%) and 40 (13·2%), respectively." These frequencies are depicted without clearly reflecting the denominators used, which is misleading.
Response: We have noted this and placed the denominators/ fractions that were used to obtain each frequency in the abstract lines 45-48 and results section lines 247-283. The proportions are now OK and there is no mismatch in the numbers.

•In line 199 "Plasmodium speciation by qPCR assay showed that, of the 741 infections 668 (90·15%), contained P. falciparum, 154 (20·78%) P. ovalewallikeri, 70 (9·45%), P. malariae and 64 (8·7%) P. ovalecurtisi" is very confusing to readers. Without explicitly stating that co-infections were included, the percentages exceeding 100 are very misleading to readers.
Response: We have revised the statement in the main text, results section and clarified that "Plasmodium co-infections analyses were conducted...", "Therefore, computing the raw frequencies of any detection of Plasmodium species yielded varying percentages that cumulatively exceeded 100%, consistent with the expected classification of infections containing multiple species under two or more species profiles…" Please see manuscript lines 241-246.

•In line 201-204, the counts of single species reflected are less than the sum of 544 provided.
Response: We have rectified this and all the numbers captured in this section for the single species are reflected accurately and reads as follows: "Subsequent analysis with a focus on the presence or absence of more than one species per infection revealed that a total of 535/741 (72.2%) samples had single species while 194/741 (26.1%) had multiple species infections. P. falciparum was the most frequent single-species infection occurring in 470/535 (87.9%) infections, followed by P. ovalewallikeri in 34/535 (6.4%), P. malariae in 18/535 (3.4%), and P. ovalecurtisi in 13/535 (2.4%) …" Please see lines 252-258 in the manuscript.

•In general, the statistics and other analyses have not been performed to a high technical standard and are not described in sufficient detail, which can be very confusing to readers, limiting the potential of replicating similar studies in the future.
Response: We have revised the manuscript and described in detail the statistical tests used for data analysis in the data management and statistical analysis section in lines 208-222.
•The discussion and conclusions provided are very repetitive/redundant and do not clearly distinguish the alignment to the data presented in the document and the

Powered by Editorial Manager® and ProduXion Manager® from Aries Systems Corporation

	alignment towards policy recommendations.<br>Response: We have revised the discussion and conclusion section to omit repetitive and redundant statements. The data has been well discussed and aligned towards policy recommendations, lines 412-419.<br><br>•The document requires stringent language revision to correct some grammar errors and syntax. Some sentences are way too long. For instance, in line 268-272 “Studies on inter-parasite interactions within human hosts suggest that parasites often interact with unrelated competitors within the host (74) and use this information to prioritize the resource allocation framework opting to increase replication to occupy most of the red blood cells at the expense of the development of sexual stages as one of the exclusion strategies”. The term “use this information” in relation to parasites is misleading and should be replaced with a scientifically acceptable term.<br>Response: We have revised the manuscript and corrected all the grammatical errors and syntax. We have replaced the word “information” with “mechanism” in line 328. However, some of the long sentences could not be shortened further as it would lose meaning.<br><br>•In line 106, there is a full stop before the citations.<br>Response: We have corrected this and the full stop before the citation has been removed.<br><br>•Line 330 “Finally, host immunity was not characterized were not the endpoint of this study” is grammatically very poor. Other grammatical and syntax errors have been pointed/highlighted in the copy of the reviewed manuscript.<br>Response: We have rectified this statement to read as follows: “Finally, host immunity was not characterized in this study because it was not one of the endpoints for the study…” Please see line 409-410.<br><br>•The research was conducted as per the international ethical standards. And the data availability guidelines have been adhered to.<br>Response: We have revised the manuscript and included a statement on data availability after the main text, line 436-438.<br><br>Closing statement.<br>Many thanks for the comments and queries, we hope the manuscript is well structured.
**Additional Information:**	
**Question**	**Response**
**Financial Disclosure**<br><br>Enter a financial disclosure statement that describes the sources of funding for the work included in this submission. Review the submission guidelines for detailed requirements. View published research articles from *PLOS ONE* for specific examples.<br><br>This statement is required for submission and **will appear in the published article** if the submission is accepted. Please make sure it is accurate.	Yes

**Funded studies**<br>Enter a statement with the following details:<br>• Initials of the authors who received each award<br>• Grant numbers awarded to each author<br>• The full name of each funder<br>• URL of each funder website<br>• Did the sponsors or funders play any role in the study design, data collection and analysis, decision to publish, or preparation of the manuscript?<br><br>Did you receive funding for this work?	
Please add funding details.<br>as follow-up to "**Financial Disclosure**<br><br>Enter a financial disclosure statement that describes the sources of funding for the work included in this submission. Review the submission guidelines for detailed requirements. View published research articles from *PLOS ONE* for specific examples.<br><br>This statement is required for submission and **will appear in the published article** if the submission is accepted. Please make sure it is accurate.<br><br>**Funded studies**<br>Enter a statement with the following details:<br>• Initials of the authors who received each award<br>• Grant numbers awarded to each author<br>• The full name of each funder<br>• URL of each funder website<br>• Did the sponsors or funders play any role in the study design, data collection and analysis, decision to publish, or preparation of the manuscript?<br><br>Did you receive funding for this work?"	Funding for this study was provided by the Armed Forces Health Surveillance Branch and its Global Emerging Infections Surveillance Section (Grant P0209_15_KY). The funder of the study played no role in study design, data collection, data analysis, data interpretation, or writing of the report.
Please select the country of your main research funder (please select carefully as in some cases this is used in fee calculation).	UNITED STATES - US

as follow-up to "**Financial Disclosure**<br><br>Enter a financial disclosure statement that describes the sources of funding for the work included in this submission. Review the submission guidelines for detailed requirements. View published research articles from *PLOS ONE* for specific examples.<br><br>This statement is required for submission and **will appear in the published article** if the submission is accepted. Please make sure it is accurate.<br><br>**Funded studies**<br>Enter a statement with the following details:<br>• Initials of the authors who received each award<br>• Grant numbers awarded to each author<br>• The full name of each funder<br>• URL of each funder website<br>• Did the sponsors or funders play any role in the study design, data collection and analysis, decision to publish, or preparation of the manuscript?<br><br>Did you receive funding for this work?"	
**Competing Interests**<br><br>Use the instructions below to enter a competing interest statement for this submission. On behalf of all authors, disclose any competing interests that could be perceived to bias this work—acknowledging all financial support and any other relevant financial or non-financial competing interests.<br><br>This statement is required for submission and **will appear in the published article** if the submission is accepted. Please make sure it is accurate and that any funding sources listed in your Funding Information later in the submission form are also declared in your Financial Disclosure statement.	The authors have declared that no competing interests exist.

View published research articles from *PLOS ONE* for specific examples.<br><br>**NO authors have competing interests**<br><br>Enter: *The authors have declared that no competing interests exist.*<br><br>**Authors with competing interests**<br><br>Enter competing interest details beginning with this statement:<br><br>*I have read the journal's policy and the authors of this manuscript have the following competing interests: [insert competing interests here]*<br><br>* typeset	
**Data Availability**<br><br>Authors are required to make all data underlying the findings described fully available, without restriction, and from the time of publication. PLOS allows rare exceptions to address legal and ethical concerns. See the PLOS Data Policy and FAQ for detailed information.<br><br>A Data Availability Statement describing where the data can be found is required at submission. Your answers to this question constitute the Data Availability Statement and **will be published in the article**, if accepted.<br><br>**Important:** Stating 'data available on request from the author' is not sufficient. If your data are only available upon request, select 'No' for the first question and explain your exceptional situation in the text box.<br><br>Do the authors confirm that all data underlying the findings described in their manuscript are fully available without restriction?	Yes - all data are fully available without restriction
Additional data availability information:	Tick here if your circumstances are not covered by the questions above and you need

	the journal’s help to make your data available.
**Ethics Statement**<br><br>Enter an ethics statement for this submission. This statement is required if the study involved:<br><br>• Human participants<br>• Human specimens or tissue<br>• Vertebrate animals or cephalopods<br>• Vertebrate embryos or tissues<br>• Field research<br><br>Write "N/A" if the submission does not require an ethics statement.<br><br>General guidance is provided below. Consult the submission guidelines for detailed instructions. **Make sure that all information entered here is included in the Methods section of the manuscript.**	These studies were approved by Kenya Medical Research Institute (KEMRI), Scientific and Ethics Review Unit (SERU), and Walter Reed Army Institute of Research (WRAIR) institutional review boards (protocol numbers: KEMRI 1330/WRAIR 1384 and KEMRI 2721/ WRAIR 2064. A written informed consent /assent was obtained from each of the study participants. The confidentiality of information obtained from study participants was assured by using unique identifiers.

**Format for specific study types**<br><br>**Human Subject Research (involving human participants and/or tissue)**<br>• Give the name of the institutional review board or ethics committee that approved the study<br>• Include the approval number and/or a statement indicating approval of this research<br>• Indicate the form of consent obtained (written/oral) or the reason that consent was not obtained (e.g. the data were analyzed anonymously)<br><br>**Animal Research (involving vertebrate animals, embryos or tissues)**<br>• Provide the name of the Institutional Animal Care and Use Committee (IACUC) or other relevant ethics board that reviewed the study protocol, and indicate whether they approved this research or granted a formal waiver of ethical approval<br>• Include an approval number if one was obtained<br>• If the study involved *non-human primates*, add *additional details* about animal welfare and steps taken to ameliorate suffering<br>• If anesthesia, euthanasia, or any kind of animal sacrifice is part of the study, include briefly which substances and/or methods were applied<br><br>**Field Research**<br><br>Include the following details if this study involves the collection of plant, animal, or other materials from a natural setting:<br>• Field permit number<br>• Name of the institution or relevant body that granted permission	
Data Availability<br><br>Before publication, Authors are required to make fully available and without restriction all data underlying their findings. Sharing the data underlying your findings increases the visibility, credibility, and impact of your work, while supporting transparency and future scientific discovery. Please see our PLOS Data Policy page for detailed information on this policy.</p><br>A Data Availability	All relevant data are within the paper and its Supporting Information files.

Statement, detailing where the data can be accessed, is required at first submission. Insert your Data Availability Statement in the box below.</p><br><br>Please see the data reporting of our submission guidelines for instructions on what you need to include in your Data Availability Statement. Examples of acceptable Data Availability Statements are included here for reference:</p><br> <br> - The minimal data set [and accompanying code, where generated] is available at [repository name] via [DOI hyperlink] <br> - No data was generated by this study. The following existing data sources were used: [dataset name] from [dataset location] available via [web address or DOI hyperlink]. <br> - For Study Protocols: No datasets were generated or analysed during the current study. All relevant data from this study will be made available upon study completion. <br> <br><br><i>PLOS allows rare exemptions to address legal and ethical concerns. If you have legal or ethical restrictions, please use the box below to detail these in full sentences for the Journal team to consider.</i></p><br><br>This statement will be typeset if the manuscript is accepted for publication.</p>	

Manuscript

Click here to access/download;Manuscript;Jackline_Manuscript_Clean_11Ma

High prevalence of *Plasmodium ovalewallikeri* in co-infections with *Plasmodium falciparum* in symptomatic than asymptomatic *Plasmodium* parasite infections in Kisumu, Kenya

Jackline Juma[1], Oliver J. Watson[4], Dennis Juma[1], Benjamin Opot[1], Gladys Chemwor[1], Mary Mutahi[3], Raphael Okoth[1], Edwin W. Mwakio[1], Redemptah Yeda[1], Charles Okello[1], Agnes Cheruiyot[1], Duke Omariba[1], Liliana Dayo[9], Hellen A. Ogola[9], Gladys Biwott[1], Charles Adega[1], Fredrick Oluoch[9], Vincent Omondi[9], George Otieno[9], Edwin Kamau[6], Ben Andagalu[1], Peter Sifuna[1], Amanda Roth[7], Sammy Khagayi [8], Eric C. Garges[5], Bernhards Ogutu[2], Hoseah M. Akala[1]*

[1]Department of Emerging and Infectious Diseases, Walter Reed Army Institute of Research-Africa/Centre for Clinical Research, Kenya Medical Research Institute, P. O. Box 54 – 40100, Kisumu, Kenya

[2] Centre for Clinical Research, Kenya Medical Research Institute

[3]The Kenya Medical Research Institute (KEMRI) / Wellcome Trust Research Programme, Centre for Geographic Medicine Research-Coast, Kilifi, Kenya.

[4]Medical Research Council, Centre for Global Infectious Disease Analysis, Department of Infectious Disease Epidemiology, Imperial College London

[5]Walter Reed Army Institute of Research - Africa

[6]Department of Pathology and Laboratory Medicine, Tripler Amy Medical Center, Honolulu, HI 96589, United States of America.

[7]Medical Communications for Combat Casualty Care, 1540 Porter Street, Fort Detrick MD21702.

[8] Centre for Global Health Research, Kenya Medical Research Institute, Kisumu, Kenya

[9]Department of Health County Government of Kisumu, Ministry of Health, Kenya.

*Correspondence to Dr. Hoseah M. Akala, Department of Emerging and Infectious Diseases Walter Reed Army Institute of Research-Africa/Centre for Clinical Research, Kenya Medical Research Institute, P. O. Box 54 – 40100, Kisumu, Kenya. Tel. +254722329845. E-mail: Hoseaakala@yahoo.com ; Hosea.akala@usamru-k.org

Abstract

Plasmodium falciparum (*Pf*), *Plasmodium ovalewallikeri* (*Pow*), *Plasmodium ovalecurtisi* (*Poc*), and *Plasmodium malariae* (*Pm*) coexists in Kenya sympatrically. Some infections progress to classical symptoms of malaria prompting hospital visits, while others remain asymptomatic. There is limited information on whether the species composition status of infections in endemic areas influences progression to either asymptomatic or symptomatic infection. This cross-sectional study determined the composition of *Plasmodium* species among symptomatic versus asymptomatic participants in Kombewa, Kenya.

Between 2015 and 2016, samples from 741 individuals, comprising of 437 from symptomatic at Kombewa Subcounty hospital and 304 from asymptomatic participants from within Kombewa Subcounty hospital's catchment area were analyzed for *Plasmodium* species composition using real-time qPCR assay targeting the small subunit ribosomal RNA (ssrRNA) gene. R Studio was used to describe *Plasmodium* species prevalence, and the odds of occurring within a given infection presentation.

Co-infection analysis revealed 741 samples predominantly contained *Pf* 655/741 (88.3%) followed by *Pow* 153/741 (20.6%), *Pm* 70/741 (9.4%) and *Poc* 64/741 (8.6%); 12/741 (1.62%) did not amplify any species. A total of 535/741 (72.2%) contained single species, and 194/741 (26.18%) multiple species. Species distribution differed significantly, with *Pf* and *Pow* more prevalent in symptomatic cases, while *Pm* and *Poc* were more common in asymptomatic individuals (p=0.000). *Pm* and *Poc* single-species infections occurred among asymptomatic infections only. There were significantly higher odds of multiple species occurrence for infections containing *Pf*/*Pow* 92 (95.8%) among symptomatic than asymptomatic infections (OR 1.5, 95% CI [1.36-1.65], p = 0.000). Overall, mixed-species infections involving *Pf* and *Pow* were significantly more common in symptomatic individuals, posing a risk of false diagnosis by *Pf*-Histidine-Rich Protein 2 test. This underscores the need for deployment of interventions that target all *Plasmodium* species. These findings of burden across symptomatic vs asymptomatic infections suggest need for determination of the role of species composition in disease progression.

Key words

Plasmodium species, symptomatic, asymptomatic

Introduction

Human malaria infection is attributed to six *Plasmodium* species namely, *P. falciparum, P. ovalecurtisi, P. ovalewallikeri, P. malariae, P.vivax* and *P. knowlesi* (1-3). *P. ovalewallikeri* and *P. ovalecurtisi* are now considered two morphologically identical but distinct species and the names *P. ovalewallikeri* and *P. ovalecurtisi* are preferred over the

classical trinominal names. The burden of these species in natural infections varies globally (4) along with their key vectors (5).

P. falciparum is responsible for the most severe form of disease and is the most prevalent in Sub-Saharan Africa (6) where it causes the highest number of fatalities globally (6). *Plasmodium vivax* is the second most dominant species that causes recurring malaria, with the highest burden in Asia and South America, and in Africa it is mainly in Ethiopia and Sudan (7). The less prevalent species, *P. ovalewallikeri* and *P. ovalecurtisi*, occur primarily in Africa (8, 9). *P. malariae* is predominantly found in South America, Asia, and Africa, and occurs mainly as mixed infections with *P. falciparum* at a lower frequency (10). Consequently, the burden and severity of disease caused by non-falciparum species, which are generally presumed to be milder than those associated with *P. falciparum,* remain poorly characterized in endemic human populations (11-13).

The World Health Organization recommends that all suspected malaria cases receive an accurate parasitological test to confirm infection before antimalarial treatment is administered (14-20). However, the parasitological diagnosis methods (21, 22) are less accurate in the diagnosis of low parasitemia (23) and distinguishing infecting species (24) in asymptomatic individuals (25) and therefore likely to underestimate the burden and complexity of infection in the population (26, 27). These methods, therefore, have limited utility in pre-elimination settings. The innovation of molecular diagnostic methods capable of detecting subclinical and submicroscopic *Plasmodium* infections (28, 29), which serve as reservoirs for ongoing transmission (30), is emerging as an important surveillance tool for malaria elimination strategies (31-34).

In Kenya, *P. falciparum* infection occurs predominantly as mono-infection or sympatrically with *P. malariae* and *P. ovalewallikeri*, and *P. ovalecurtisi*. This co-endemicity of species leads to the occurrence of blood-stage mixed *Plasmodium* species infections (35), which are mostly acquired from separate inoculations when a recently acquired infection of one species might supervene a chronic infection with another (36) or simultaneous inoculation of the sporozoites of two or more species from a doubly infected *Anopheles* mosquito (36-38). Studies have highlighted variations in the clinical presentation of disease per species relative to the unique parasite biology of each malaria species. Evaluating the species composition of infections among symptomatic individuals seeking care at health facilities and asymptomatic individuals within households is essential for understanding and addressing the rising prevalence of non-*Plasmodium falciparum* species in the context of improved access to accurate diagnosis and timely treatment, which are key for transitioning regions with declining malaria to pre-elimination.

Clinical symptoms of malaria typically develop between 7 and 85 days after initial infection, depending on the infecting *Plasmodium* species (39-43). Parasitemia may also persist in susceptible individuals for up to eight years before the onset of symptoms (42-45). Initial symptoms of malaria may include a high temperature of 38°C and above, muscle pains, and generally feeling unwell, often characterized by recurrent fever and chills (42), which often trigger a treatment-seeking response (20) once parasite replication becomes synchronous. In some cases, the infection may generate malarial parasitemia in the absence of fever or other classical symptoms and signs, in otherwise healthy individuals, therefore regarded as asymptomatic *Plasmodium* parasite carriers (21, 46, 47). Asymptomatic *Plasmodium* infections account for up to 60% of infections (48-51) in some regions. It has

been suggested that infections remain asymptomatic due to sustained submicroscopic peripheral parasitemia (52, 53), older age, and therefore partial immunity of the infected individual (54, 55) and varying malaria pyrogenic threshold (56, 57). In a 2011 study, the complex interactions between infecting *Plasmodium* species (56, 58) were shown to affect the disease outcome such as the occurrence of symptoms (59) and transmissibility, bringing in to focus the role of these interactions in either limiting or promoting disease progression.

In a region with natural infections comprising more than one *Plasmodium* species (60, 61), there is uncertainty about whether infection with multiple species reduces or promotes the incidence of symptoms in the infected individual as a means of maintaining transmissibility. Our previous study showed the prevalence of *Plasmodium* species co-infections changes over time, among symptomatic individuals seeking for treatment at selected hospitals across Kenya (62), but did not describe the burden among asymptomatic infections. Given the varying disease progression among *Plasmodium* species such as longer period of case management for relapsing species, estimating of the burden of species in the population is useful in informing choice of intervention needed to lower malaria in line with the WHO goal that targets 90% reduction of malaria by 2030. This study aimed to estimate the composition of *Plasmodium* parasite species among symptomatic versus asymptomatic infections in Kisumu County in western Kenya.

Materials and methods

Ethical considerations

Ethical approval was obtained from Kenya Medical Research Institute (KEMRI), Scientific and Ethics Review Unit (SERU), and Walter Reed Army Institute of Research

(WRAIR) institutional review boards (protocol numbers: KEMRI 1330/WRAIR 1384 and KEMRI 2721/ WRAIR 2064. A written informed consent /assent was obtained from each of the study participants. The confidentiality of information obtained from study participants was assured by using unique identifiers.

Study site and sample collection

Kombewa Health and Demographic Surveillance System (HDSS) grew out of the Kombewa Clinical Research Centre in 2007 and has since established itself as a platform for conducting nested studies and local disease surveillance, according to a study by Sifuna and co-workers. This study was conducted within Kombewa HDSS, which forms the main catchment of the malaria cases for the Kombewa Sub-County hospital, an area that is categorized as a rural Lake Endemic Zone with perennial and intense malaria transmission as per the National Malaria Control Programme 2016 (NMCP).

This sub-study analysed *Plasmodium*-positive samples obtained from two studies that were conducted concurrently between 2015 and 2016. The first study was entitled "Epidemiology of malaria and drug sensitivity patterns in Kenya", which commenced from 1st September 2015 to 1st June 2016. The second study was entitled, "Baseline Epidemiology of Malaria Gametocyte Carriage and Transmission Dynamics in a Holoendemic Transmission Setting in Kisumu County, Western Kenya", which commenced on 18th March 2015 to 1st February 2016.

The first study enrolled symptomatic participants aged six months and above presenting at Kombewa Sub-County Hospital with clinical features suggestive of malaria, including fever (≥37.5 °C) or a history of fever within the preceding 48 hours accompanied by symptoms such as chills, headache, malaise, or sweating. Participants who were clinically

stable, consistent with uncomplicated malaria, and who tested positive for malaria by rapid diagnostic test (RDT) and/or microscopy were eligible for inclusion. Under this study, these individuals were referred to as symptomatic malaria participants. These individuals were enrolled into the study upon providing written informed consent/assent. Each participant provided 2-3 ml of whole blood for malaria parasite species genotyping alongside other endpoints of the main study reported elsewhere (63, 64). All *Plasmodium Plasmodium* positive cases were treated with artemether-lumefantrine (AL) (Coartem®), as per the Kenya Ministry of Health's recommended case management guidelines for uncomplicated malaria. The healthcare workers administered and observed the taking of the first dose of AL 20mg/120mg tablets.

The second study enrolled asymptomatic participants and sought to estimate the level of gametocyte carriage in the study population by PCR. These comprised individuals of all ages, without clinical features suggestive of malaria, such as fever (≥37.5 °C) or history of fever within the previous 48 hours, and absence of symptoms such as chills, headache, malaise, or sweating. Under this study, these individuals were referred to as asymptomatic malaria participants. Members of selected households within Kisumu West sub-County, which is the main catchment for the Kombewa sub-County hospital (**Fig.1 Map of Kombewa**), willing to be tested for the presence of malaria parasites were enrolled into the study upon providing written informed consent/assent. During this household visit, vital signs were measured to rule out symptoms of malaria. Based on these readouts, each of the otherwise healthy participants provided 4 ml of whole blood for the diagnosis of malaria. Each sample was tested within 24 hours, and all participants found to have *Plasmodium* genus

infection were treated with the attending healthcare worker administering the first dose of AL 20mg/12mg tablets.

Map data from OpenStreetMap (https://www.openstreetmap.org/copyright). The base maps used in all three images in Figure 1 were obtained from Open AFRICA

*****This map was custom generated in-house using the ArcGIS Version 10.3.1 (Environmental Systems Research Institute, Red-lands, CA, USA) software.

Fig 1: Map showing Kombewa region where Kombewa sub-County hospital is located.

*Note

Molecular assay

Nucleic acids extraction

The parasite nucleic acids were extracted from blood samples obtained from individuals enrolled in the two studies using a commercial extraction kit (QIAamp DNA Blood Mini Kit, Qiagen) as recommended by the manufacturer's instructions.

Plasmodium genus diagnosis by molecular methods

The extracted nucleic acids were analysed for malaria diagnosis using *Plasmodium* genus-specific qPCR assay as previously described (65, 66). PCR amplified the *Plasmodium* genus 18S ribosomal ribonucleic acid (rRNA) gene using primers listed in **Table 1**. The RN*ase* P gene, which is a human housekeeping gene, was used as an extraction and qPCR assay control.

Detection of species composition of infections

All samples that were diagnosed as positive for the *Plasmodium* genus were further characterized for species composition as described by (65, 66) using a separate set of primers indicated in **Table 1.** The assays for characterization of *Plasmodium falciparum* and *P. malariae* had similar PCR reaction components and conditions as the genus-specific qPCR assay except for the primers used. Genus-specific primers were replaced respectively by the species-specific primers (28, 67) **(Table 1**). Each experiment included at least one reaction mixture without DNA as a negative control.

Table 1: Primers and probes used for screening and identification of *Plasmodium* species

Primers Probes	Sequence 5'-3'	Target genus/species
PLU F	GCTCTTTCTTGATTTCTTGGATG	*Plasmodium* spp.
PLU R	AGCAGGTTAAGATCTCGTTCG	*Plasmodium* spp.
PLU P	ATGGCCGTTTTTAGTTCGTG	*Plasmodium* spp.
RNaSP F	TGTTTGCAGATTTGGACCTGC	Human RNase p
RNaSeP R	AATAGCCAAGGTGGAGCGGCT	Human RNase p
RNaseP P	TGCGCGGACTTGTGGA	Human RNase p
FAL F	ATTGCTTTTGAGAGGTTTTGTTACTT	*P. falciparum*
FAL R	GCTGAGTATTCAAACACAATGAACTCAA	*P. falciparum*
FAL P	CATAACAGACGGGTAGTCAT	*P. falciparum*
MAL F	GCATGGAATTTTGTTACTTTGA	*P. malariae*
MAL R	ATGCCTGTAGTATTCAACACAGAAAC	*P. malariae*
MAL P	TGTTCAAAGCAAACAGTTAAAACA	*P. malariae*
OVA F	TTTTGAAGAATACATTAGGATACAATTAATG	*P. ovale curtisi*
OVA R	CATGCTTCCTCTAAGAAGCTTTACAAT	*P. ovale*
OVA-V F	TTTTGAAGAATATATTAGGATACATTATAG	*P. ovale Wallikeri*
OVA-V R	CATCGTTCCTCTAAGAAGCTTTACAAT	*P. ovale Wallikeri*
OVA P	CCTTTTCCCTATTCTACTTAATTCGCAATTCATG	*P. ovale curtisi*
OVA-V P	CCTTTTCCCTACTTAATTCGCTATTCATTG	*P. ovale Wallikeri*

Data management and statistical analysis

Descriptive analyses were used to describe categorical data comprising age categories, gender, and qPCR species results summarized as frequencies and proportions. Continuous variables, including age in years, were first assessed for normality using the

Shapiro–Wilk test and then expressed as median together with their corresponding interquartile ranges (IQR) when non-normal distributions were observed. The geometric mean was used as a measure of central tendency for parasite density.

Descriptive statistics were employed to illustrate the burden of *Plasmodium* species among the participants with positive infections. For inferential statistics, proportions were compared using the Chi-square test as appropriate for the categorical data. Wilcoxon rank-sum test was used for non-parametric continuous variables. Logistic regression analysis was also conducted to assess the varying odds of *Plasmodium* species composition. All analyses were performed using R Studio version 4.5.1. Statistical analyses were performed at the 5% significance level and the corresponding 95% confidence interval (CI). The critical significance level was set at $p \leq 0.05$.

Results

A total of 741 samples comprising 437 (58.9%) of symptomatic participants and 304 (41.0%) of the asymptomatic participants were analyzed. The description of the study population is summarized in **Table 2**. Metadata was available for 733/741 participants though species analyses data was available for all the 741 samples. The median ages for all age categories and number of participants per age category varied significantly across the two infections presentations χ^2 test p=0.000 (**Table 2**). The categories of less than 5 years were highest in symptomatic infections, 258 (79.4%) while over 18 years were highest in asymptomatic infections 81 (66.9%) (**Table 2**). Wilcoxon rank-sum test revealed a significantly higher age among the asymptomatic, median 9 years; IQR, 5-20.5 than symptomatic 4 years; IQR, 3-6, p=0.000, r=0.38. Gender was comparable between the symptomatic and asymptomatic infections. The geometric mean of parasite density per microliter of blood was significantly higher among the symptomatic (47504.83/ul, 95%CI 39131.25-57670.26) than asymptomatic infections (784.11/ul, 95%CI 784.11-1667.1) (**Table 2**).

Table 2: Metadata among symptomatic and asymptomatic *Plasmodium* infections

Variable	Symptomatic	Asymptomatic	p value
**Age years:**			
**median (IQR)**	4 (3-6)	9 (5-21)	0.000
**Age category:**			
**<5 years (n)%**	258 (79.38)	67 (20.62)	0.000
**5-18 years (n)%**	131 (45.64)	156 (54.36)	
**>18 years (n)%**	40 (33.06)	81 (66.94)	
**Gender:**			
**Male (n)%**	195 (60.56)	127 (39.44)	0.323

**Female (n)%**	234 (56.93)	177 (43.07)	
**Parasite density:**			
**Geometric mean**	47504.83	1143.33	0.000
**(95% CI)**	(39131.25-57670.26)	(784.11-1667.107)	

Table 2: Metadata for symptomatic and asymptomatic *Plasmodium* infections.

Plasmodium co-infections analyses were conducted, and the species composition analyses detected the presence of single *Plasmodium* species in most infections and presence of two or three or four *Plasmodium* species in a subset of infections. Therefore, computing the raw frequencies of any detection of *Plasmodium* species yielded varying percentages that cumulatively exceeded 100%, consistent with the expected classification of infections containing multiple species under two or more species profiles. On this basis, *Plasmodium* species detection by qPCR assay showed that, 655/741 (88.3%) of the infections contained *P. falciparum,* 153/741 (20.7%) *P. ovalewallikeri,* 70/741 (9.4%) *P. malariae,* and 64/741 (8.7%) *P. ovalecurtisi* without consideration of the number of species per infection (**Table 3**). However, 12/741 (1.6%) ~~amplifying~~ for presence of *Plasmodium* genus did not amplify for any species.

Subsequent analysis with a focus on the presence or absence of more than one species per infection revealed that a total of 535/741 (72.2%) samples had single species while 194/741 (26.1%) had multiple species infections. *P. falciparum* was the most frequent single-species infection occurring in 470/535 (87.9%) infections, followed *by P. ovalewallikeri* in 34/535 (6.4%), *P. malariae* in 18/535 (3.4%), and *P. ovalecurtisi* in 13/535 (2.4%). Notably, all the *P. malariae* and *P. ovalecurtisi* single-species infections were detected among asymptomatic infections only. Multiple species infections included *Pf*/*Pow* 96/194 (49.5%), being dominant followed by *Pf*/*Pm* 41/194 (21.1%), *Pf*/*Poc* 30/194 (15.5), *Pf*/*Pow*/*Poc*

15/194 (7.7%), *Pm/Pow* 5/194 (2.6%), *Pf/Pm/Poc* 2/194 (1.0%), *Pm/Poc* 2/194 (1.0%), *Pf/Pm/Pow* 1/194 (0.5%), *Pm/Poc/Pow* 1/194 (0.5%), *Pow/Poc* 1/194 (0.5%).

Table 3: Overall *Plasmodium* species frequency

**Species**	**n**	**%**
P. falciparum	655	88.4
P. ovalewallikeri	153	20.7
P.malariae	70	9.5
P.ovalecurtisi	64	8.7

Table 3: Overall *Plasmodium* species frequency detected in the samples

Symptomatic infections

Assessment of frequency of single *Plasmodium* species showed that *P. falciparum* 262/437 (60%) and *P. ovale wallikeri* 15/437 (3.4%) were the only single species detected among the symptomatic infections (**Fig. 2**). The symptomatic individuals harbored multiple *Plasmodium* species, comprising 2 species; *PfPow* 92/437 (21.1%), *PfPm* 28/437 (6.4%), *PfPoc* 22/437 (5%), *PowPoc* 1/437 (0.2%) three species; *PfPowPoc* 15/437 (3.4%), *PfPmPoc* 2/437 (0.5%) (**Fig. 2**). Further, neither *P. ovalecurtisi* nor *P. malariae* occurred as a single species infection among symptomatic participants during this study period (**Fig. 2**).

Fig 2: *Plasmodium* single species and mixed species frequency among symptomatic infections by molecular diagnosis. Symptomatic infections refer to infections that generate malarial parasitemia alongside symptoms.

Asymptomatic infections

Single *Plasmodium* species infections included *P. falciparum* detected in 218/304 (71.7%) of the asymptomatic infections as the most prevalent species, followed by *P. ovalewallikeri* 18/304 (5.9%), *P. malariae* 16/304 (5.3%), and *P. ovalecurtisi* 15/304 (4.9%),

respectively (**Fig 3**). Multiple species infections comprised double; *PfPm* 14/304 (4.6%), *PfPoc* 8/304 (2.6%), *PmPow* 5/304 (1.6%), *PfPow* 5/304 (1.6%), *PmPoc* 2/304 (0.7%), or triple *PfPmPow* 2/304 (0.7%) and *PmPocPow* 1/304 (0.3%) infections (**Fig 3**).

Fig 3: *Plasmodium* mixed and single species frequency among asymptomatic infections by molecular diagnosis. Asymptomatic *Plasmodium* parasite carriers are the infections that generate malarial parasitemia in the absence of fever or other classical symptoms and signs, in otherwise healthy individuals.

The *P. falciparum* single-species infections were more prevalent in asymptomatic than symptomatic individuals **(Figs. 2 & 3**). Multiple species were more likely to be symptomatic than asymptomatic infections. Assessment of the role of infection composition in disease presentation showed that *P. falciparum* with *P. ovalewallikeri*; *P. falciparum* with *P. ovalecurtisi*, and *P. falciparum, P. ovalewallikeri* with *P. ovalecurtisi* mixed-species infections had higher odds of progressing to symptomatic infection than remaining asymptomatic (**Fig. 4**). The mixed species had increased odds of symptomatic infection (**Fig. 4**).

Fig. 4: Odds of *Plasmodium* species composition in infection presentation. The blue represents no significant association, while the red color represents a significant association.

Discussion

This study concurrently compared the prevalence of *Plasmodium* species among symptomatic individuals seeking treatment at the Kombewa Sub-County hospital versus asymptomatic individuals in households within the same geographic location that doubles up as the catchment of the hospital using qPCR. About 13% and 26% prevalence of multiple species was detected among asymptomatic and symptomatic infections, respectively. Studies in West and Central Africa focusing on school-going children have also shown a higher burden of non-*falciparum* species in asymptomatic *Plasmodium* parasite carriers (50, 51). This finding is contrary to a recent meta-analysis showing no difference in the prevalence of either *P. malariae* or *P. ovale spp* between symptomatic and asymptomatic individuals (68). Additionally, large-scale studies of non-*falciparum* parasites report conflicting results for those from West Africa (69) versus those from central or East Africa (70). These varying observations underscore the peculiarity of *Plasmodium* infection presentation across different disease endemicities and are evocative of the need for characterizing species composition in diverse disease geo-ecotypes for effective targeting.

The occurrence of *Plasmodium* parasitemia in the absence of classical malaria symptoms, and the mechanisms that influence the transition from an asymptomatic infection to symptomatic infection, remain subjects of ongoing debate. A prospective study by Chen and coworkers showed that some individuals harboring *Plasmodium* parasites can either remain healthy for an unlimited amount of time and referred to as asymptomatic (21) while others progress to disease with malaria symptoms within 7-85 days and seek for the treatment depending on various factors, including the species composition of the infection (47, 58, 71,

72). This study detected a higher frequency of mixed species infection among symptomatic than asymptomatic individuals, an observation that appears to align with the theory that harboring multiple species potentiates the transition of infections to the symptomatic phase.

Studies on inter-parasite interactions within human hosts suggest that parasites often interact with unrelated competitors within the host (73) and use this mechanism to prioritize the resource allocation framework, opting to increase replication to occupy most of the red blood cells at the expense of the development of sexual stages as one of the exclusion strategies. The changes in resource utilization resulting from the presence of the additional species have been associated with modulation of the host response (59, 74-76) toward either the persistence of the asymptomatic phase of the infection (77) or progression to symptomatic disease in the presence of virulence-enhancing species mixtures (78). The detection of a higher frequency of multiple species infections among the symptomatic than asymptomatic, with a reciprocal higher frequency of single species infections in asymptomatic than symptomatic, by this study appears to suggest that infection with more than one species is likely to progress to a more symptomatic state than single-species infections as reported elsewhere (26, 27). It was, however, surprising that asymptomatic individuals harbored more diverse species profiles than symptomatic individuals based on PCR-based diagnosis.

The composition of mixed species infections varied significantly between the symptomatic and asymptomatic infections. For example, there was a ten-fold higher prevalence of co-infections containing *P. falciparum* and *P. ovalewallikeri* among the symptomatic individuals than among the asymptomatic individuals. A similar pattern of frequency was observed among infections containing *P. falciparum, P. ovalewallikeri,* plus *P. ovalecurtisi,* contrary to *P. falciparum/P. ovalecurtisi, or P. falciparum/P. malariae* or

either of these species in single-species infections, an observation that may suggest between-species interactions.

Evidence from malaria ecology indicates that the acquisition of an additional *Plasmodium* species within a host can modify the within-host ecological environment by introducing competition for limited resources such as red blood cells. For example, in mixed-species infections involving human malaria parasites, including *P. falciparum* and *P. vivax*, parasites may compete for host erythrocytes and other resources, which can alter parasite replication dynamics and suppress the density of competing species. These competitive interactions may consequently influence parasite fitness, disease outcomes, and transmission potential (79). Parasite biology studies show that *P. falciparum* attacks all ages of red blood cells, hence it has been hypothesized to be a "generalist" while *P. ovale* and *P. malariae* attack younger and older red blood cells, respectively, and are hypothesized to be specialists (77, 78, 80).

Studies show that in mixed-species infections, the presence of an RBC *Plasmodium* species regarded as "generalists" enhances the density of RBC specialists (76). A study monitoring blood levels of tumor necrosis factor-alpha (TNF-alpha), lactate dehydrogenase (LDH), Thrombin-antithrombin III (TAT) and elastase as drivers of malaria symptoms through modulation of host immune response among uncomplicated malaria was conducted. The study further noted that these parameters were higher per parasitized *P. ovale* erythrocyte levels than *P. falciparum*, with the former attaining full-strength host response at lower parasitemia than the latter (78). Observation of significantly higher frequency *P. falciparum* and *P. ovalewallikeri* among naturally acquired symptomatic than asymptomatic infection by this study suggests that parasitemia increases in *P. ovalewallikeri*, a specialist, given the

presence of *P. falciparum* a generalist, could be contributing to elevated immune response leading to the occurrence of symptoms among these infections. This phenomenon, which is absent in *P. ovalecurtisi* infections, highlights the biological differences between the two *P. ovale* species. Notably, *P. ovalewallikeri* single-species infections were detected among symptomatic as well as asymptomatic infections while *P. ovalecurtisi* alone was not detected among the symptomatic, suggesting that *P. ovalewallikeri* alone causes symptoms more frequently than *P. ovalecurtisi*.

Single-species infections of *P. falciparum, P. malariae, or P. ovalecurtisi* were mostly detected among asymptomatic individuals than symptomatic. Notably, *P. ovalecurtisi* single-species infections were detected in 4.8% of the asymptomatic infections while none was detected in symptomatic infections. Additionally, infections comprising *P. malariae*/*P. ovalecurtisi* or *P. malariae*/*P. ovalewallikeri* were detected in asymptomatic infections at less than 2% frequency while none were detected among symptomatic infections. *P. malariae* co-infections with *P. ovalecurtisi* or *P. ovalewallikeri* have been associated with low pyrogenic thresholds, occurring in less than 2% of the symptomatic cases (77). This infection characteristic is a challenge for malaria elimination since the chronically infected individual would remain obscure to healthcare facilities while still capable of transmitting the parasite to mosquitoes and therefore sustaining transmission to humans. Such infections have been proposed as the last battlefield for malaria elimination (81) since they will also remain undetected by the histidine-rich protein based malaria rapid diagnostic test that are expedient for population-based strategies of bringing down diseases.

This study has some limitations. The study depended on the vital signs of the participants at the time of enrolment such fever ≥37.5 °C or a history of fever within the

preceding 48 hours accompanied by symptoms such as chills, headache, malaise, or sweating, to be categorized as symptomatic participants, while those without these signs were categorized as asymptomatic. Because of the study design, it was difficult to determine when, specifically, the time-point in the previous period preceding enrolment day, at which either the asymptomatic or symptomatic individuals acquired the infection. Consequently, it was not possible to establish whether individuals with asymptomatic infections would subsequently develop symptoms as parasite densities increased to the pyrogenic threshold, or whether the infections in these individuals were chronic and would remain asymptomatic. It was again not possible to ascertain which of the infecting species in individuals harbouring multiple species infections came first and which one came later. This, therefore, made it harder to tell whether there was protectiveness proffered by some species or immune silencing by other species to delay symptoms, or associate entry of a given species with the onset of symptoms. Further, the study was not able to filter out and show within-host response to varying species composition of infections or between-species interactions within each of the mixed infection profiles to filter out specific parasite species-specific biology that infer implication to the progression of disease or epidemiology of each of the species versus presently deployed malaria interventions. Finally, host immunity was not characterized in this study because it was not one of the endpoints for the study.

Conclusion

These findings in a malaria-endemic zone suggest that intervention strategies need to consider the species composition of asymptomatic *Plasmodium* parasite infections to progress the region to the elimination phase. The ten-fold higher frequency of *P.*

ovalewallikeri species among the symptomatic than asymptomatic infections and higher *P. falciparum* single-species infections in the asymptomatic than symptomatic appears to associate this infection composition with higher likelihood of symptoms. Interventions need to focus on the mixed infections asymptomatic *Plasmodium* parasite carriers since these two serves as silent reservoirs for transmission and are a lynchpin to malaria elimination in a given region.

Acknowledgments

Material has been reviewed by the Walter Reed Army Institute of Research. There is no objection to its presentation and/or publication. The opinions or assertions contained herein are the private views of the author, and are not to be construed as official, or as reflecting true views of the Department of the Army or the Department of Defense. The investigators have adhered to the policies for protection of human subjects as prescribed in AR 70–25. We thank the individuals who participated in this study for providing these invaluable samples and time to generate these findings. We also thank the County Government of Kisumu and the Ministry of Health, Kenya, for giving us access to these samples. We thank the Commander, Walter Reed Army Institute of Research - Africa, COL. Lacy Shannon, Director, Walter Reed Army Institute of Research - Africa, Kisumu Field Stations, COL. Gerald Keller, Deputy Director General, Center for Clinical Research, Kenya Medical Research Institute, Kenya, for supporting this study and giving their permission to publish these data. We also thank all clinical staff at all the participating hospitals for their assistance.

Data Availability

All relevant data are within the paper and its Supporting Information files. Any other additional information can be availed upon requesting the corresponding author.

References

1. Ansari HR, Templeton TJ, Subudhi AK, Ramaprasad A, Tang J, Lu F, et al. Genome-scale comparison of expanded gene families in Plasmodium ovale wallikeri and Plasmodium ovale curtisi with Plasmodium malariae and with other Plasmodium species. International journal for parasitology. 2016;46(11):685-96.
2. Antinori S, Galimberti L, Milazzo L, Corbellino M. Plasmodium knowlesi: the emerging zoonotic malaria parasite. Acta tropica. 2013;125(2):191-201.
3. Snounou G, Sharp PM, Culleton R. The two parasite species formerly known as Plasmodium ovale. Trends in parasitology. 2024;40(1):21-7.
4. Bhatt S, Weiss D, Cameron E, Bisanzio D, Mappin B, Dalrymple U, et al. The effect of malaria control on Plasmodium falciparum in Africa between 2000 and 2015. Nature. 2015;526(7572):207-11.
5. Sinka ME, Bangs MJ, Manguin S, Rubio-Palis Y, Chareonviriyaphap T, Coetzee M, et al. A global map of dominant malaria vectors. Parasites & vectors. 2012;5(1):1-11.
6. Weiss DJ, Lucas TC, Nguyen M, Nandi AK, Bisanzio D, Battle KE, et al. Mapping the global prevalence, incidence, and mortality of Plasmodium falciparum, 2000–17: a spatial and temporal modelling study. The Lancet. 2019;394(10195):322-31.
7. Battle KE, Lucas TC, Nguyen M, Howes RE, Nandi AK, Twohig KA, et al. Mapping the global endemicity and clinical burden of Plasmodium vivax, 2000–17: a spatial and temporal modelling study. The Lancet. 2019;394(10195):332-43.
8. Price RN, Commons RJ, Battle KE, Thriemer K, Mendis K. Plasmodium vivax in the Era of the Shrinking P. falciparum Map. Trends in parasitology. 2020;36(6):560-70.
9. Mahittikorn A, Masangkay FR, Kotepui KU, Milanez GDJ, Kotepui M. Comparison of Plasmodium ovale curtisi and Plasmodium ovale wallikeri infections by a meta-analysis approach. Scientific reports. 2021;11(1):1-15.
10. Sutherland CJ. Persistent parasitism: the adaptive biology of malariae and ovale malaria. Trends in Parasitology. 2016;32(10):808-19.
11. Diagne A, Sambe BS, Gaba FM, Sarr I, Diatta AS, Sadio O, et al. Variable effects of non-falciparum species infections on malaria disease severity in high transmission regions in Senegal. Tropical Medicine and Health. 2024;52(1):93.
12. Sendor R, Banek K, Kashamuka MM, Mvuama N, Bala JA, Nkalani M, et al. Epidemiology of Plasmodium malariae and Plasmodium ovale spp. in Kinshasa Province, Democratic Republic of Congo. Nature communications. 2023;14(1):6618.
13. Omondi P, Musyoka B, Okai T, Kongere J, Kagaya W, Chan CW, et al. Non-random distribution of Plasmodium Species infections and associated clinical features in children in the lake Victoria region, Kenya, 2012-2018. Trop Med Health. 2024;52(1):52.
14. Zarocostas J. Malaria treatment should begin with parasitological diagnosis where possible, says WHO. BMJ: British Medical Journal (Online). 2010;340.
15. Newman RD. World Malaria Report 2011. 2012.
16. Musuva A, Ejersa W, Kiptui R, Memusi D, Abwao E. The malaria testing and treatment landscape in Kenya: results from a nationally representative survey among the public and private sector in 2016. Malaria journal. 2017;16(1):1-13.
17. Kamal-Yanni MM, Potet J, Saunders PM. Scaling-up malaria treatment: a review of the performance of different providers. Malaria journal. 2012;11(1):1-10.

18. Alonso P, Noor AM. The global fight against malaria is at crossroads. The Lancet. 2017;390(10112):2532-4.
19. Larsen DA, Ngwenya-Kangombe T, Cheelo S, Hamainza B, Miller J, Winters A, et al. Location, location, location: environmental factors better predict malaria-positive individuals during reactive case detection than index case demographics in Southern Province, Zambia. Malaria journal. 2017;16(1):1-9.
20. Hamainza B, Moonga H, Sikaala CH, Kamuliwo M, Bennett A, Eisele TP, et al. Monitoring, characterization and control of chronic, symptomatic malaria infections in rural Zambia through monthly household visits by paid community health workers. Malaria journal. 2014;13(1):1-19.
21. Chen I, Clarke SE, Gosling R, Hamainza B, Killeen G, Magill A, et al. "Asymptomatic" malaria: a chronic and debilitating infection that should be treated. PLoS medicine. 2016;13(1):e1001942.
22. Mueller I, Slutsker L, Tanner M. Estimating the burden of malaria: the need for improved surveillance. PLoS medicine. 2011;8(12):e1001144.
23. Kamau E, Alemayehu S, Feghali KC, Saunders D, Ockenhouse CF. Multiplex qPCR for detection and absolute quantification of malaria. PloS one. 2013;8(8):e71539.
24. Veron V, Simon S, Carme B. Multiplex real-time PCR detection of P. falciparum, P. vivax and P. malariae in human blood samples. Experimental parasitology. 2009;121(4):346-51.
25. Murray CK, Gasser Jr RA, Magill AJ, Miller RS. Update on rapid diagnostic testing for malaria. Clinical microbiology reviews. 2008;21(1):97-110.
26. Okyere B, Owusu-Ofori A, Ansong D, Buxton R, Benson S, Osei-Akoto A, et al. Point prevalence of asymptomatic Plasmodium infection and the comparison of microscopy, rapid diagnostic test and nested PCR for the diagnosis of asymptomatic malaria among children under 5 years in Ghana. PloS one. 2020;15(7):e0232874.
27. van Eijk AM, Sutton PL, Ramanathapuram L, Sullivan SA, Kanagaraj D, Priya GSL, et al. The burden of submicroscopic and asymptomatic malaria in India revealed from epidemiology studies at three varied transmission sites in India. Scientific reports. 2019;9(1):1-11.
28. Perandin F, Manca N, Calderaro A, Piccolo G, Galati L, Ricci L, et al. Development of a real-time PCR assay for detection of Plasmodium falciparum, Plasmodium vivax, and Plasmodium ovale for routine clinical diagnosis. Journal of clinical microbiology. 2004;42(3):1214-9.
29. Kamau E, Tolbert LS, Kortepeter L, Pratt M, Nyakoe N, Muringo L, et al. Development of a highly sensitive genus-specific quantitative reverse transcriptase real-time PCR assay for detection and quantitation of Plasmodium by amplifying RNA and DNA of the 18S rRNA genes. Journal of clinical microbiology. 2011;49(8):2946-53.
30. Ouédraogo AL, Eckhoff PA, Luty AJ, Roeffen W, Sauerwein RW, Bousema T, et al. Modeling the impact of Plasmodium falciparum sexual stage immunity on the composition and dynamics of the human infectious reservoir for malaria in natural settings. PLoS pathogens. 2018;14(5):e1007034.
31. Gholizadeh S, Karimi NN, Zakeri S, Djadid ND. The role of molecular techniques on malaria control and elimination programs in iran: A review article. Iranian journal of parasitology. 2018;13(2):161.
32. Tedla M. A focus on improving molecular diagnostic approaches to malaria control and elimination in low transmission settings. Parasite epidemiology and control. 2019;6:e00107.
33. Njuguna P, Maitland K, Nyaguara A, Mwanga D, Mogeni P, Mturi N, et al. Observational study: 27 years of severe malaria surveillance in Kilifi, Kenya. BMC medicine. 2019;17(1):1-9.

34. Cheaveau J, Mogollon DC, Mohon MAN, Golassa L, Yewhalaw D, Pillai DR. Asymptomatic malaria in the clinical and public health context. Expert review of anti-infective therapy. 2019;17(12):997-1010.
35. Zimmerman PA, Mehlotra RK, Kasehagen LJ, Kazura JW. Why do we need to know more about mixed Plasmodium species infections in humans? Trends in parasitology. 2004;20(9):440-7.
36. Portugal S, Drakesmith H, Mota MM. Superinfection in malaria: Plasmodium shows its iron will. EMBO reports. 2011;12(12):1233-42.
37. Imwong M, Nakeesathit S, Day NP, White NJ. A review of mixed malaria species infections in anopheline mosquitoes. Malaria journal. 2011;10(1):1-12.
38. Beier MS, Schwartz IK, Beier JC, Perkins PV, Onyango F, Koros JK, et al. Identification of malaria species by ELISA in sporozoite and oocyst infected Anopheles from western Kenya. The American journal of tropical medicine and hygiene. 1988;39(4):323-7.
39. Verra F, Angheben A, Martello E, Giorli G, Perandin F, Bisoffi Z. A systematic review of transfusion-transmitted malaria in non-endemic areas. Malaria Journal. 2018;17:1-14.
40. Orish V, Afutu L, Ayodele O, Likaj L, Marinkovic A, Sanyaolu A, editors. A 4-day incubation period of Plasmodium falciparum infection in a nonimmune patient in Ghana: a case report. Open Forum Infectious Diseases; 2019: Oxford University Press US.
41. Okafor CN, Finnigan NA. Plasmodium ovale malaria. 2018.
42. Bartoloni A, Zammarchi L. Clinical aspects of uncomplicated and severe malaria. Mediterranean journal of hematology and infectious diseases. 2012;4(1).
43. Krajden S, Panisko DM, Tobe B, Yang J, Keystone JS. Prolonged infection with Plasmodium falciparum in a semi-immune patient. Trans R Soc Trop Med Hyg. 1991;85(6):731-2.
44. Greenwood T, Vikerfors T, Sjöberg M, Skeppner G, Färnert A. Febrile Plasmodium falciparum malaria 4 years after exposure in a man with sickle cell disease. Clinical Infectious Diseases. 2008;47(4):e39-e41.
45. Szmitko PE, Kohn ML, Simor AE. Plasmodium falciparum malaria occurring 8 years after leaving an endemic area. Diagnostic microbiology and infectious disease. 2009;63(1):105-7.
46. Moonen B, Cohen JM, Snow RW, Slutsker L, Drakeley C, Smith DL, et al. Operational strategies to achieve and maintain malaria elimination. The Lancet. 2010;376(9752):1592-603.
47. Lindblade KA, Steinhardt L, Samuels A, Kachur SP, Slutsker L. The silent threat: asymptomatic parasitemia and malaria transmission. Expert review of anti-infective therapy. 2013;11(6):623-39.
48. Bottius E, Guanzirolli A, Trape J-F, Rogier C, Konate L, Druilhe P. Malaria: even more chronic in nature than previously thought; evidence for subpatent parasitaemia detectable by the polymerase chain reaction. Transactions of the Royal Society of Tropical Medicine and Hygiene. 1996;90(1):15-9.
49. Rouamba T, Samadoulougou S, Ouédraogo M, Hien H, Tinto H, Kirakoya-Samadoulougou F. Asymptomatic malaria and anaemia among pregnant women during high and low malaria transmission seasons in Burkina Faso: household-based cross-sectional surveys in Burkina Faso, 2013 and 2017. Malaria journal. 2021;20(1):1-13.
50. Abdulraheem MA, Ernest M, Ugwuanyi I, Abkallo HM, Nishikawa S, Adeleke M, et al. High prevalence of Plasmodium malariae and Plasmodium ovale in co-infections with Plasmodium falciparum in asymptomatic malaria parasite carriers in southwestern Nigeria. International journal for parasitology. 2022;52(1):23-33.
51. Nundu SS, Culleton R, Simpson SV, Arima H, Muyembe J-J, Mita T, et al. Malaria parasite species composition of Plasmodium infections among asymptomatic and symptomatic school-age

children in rural and urban areas of Kinshasa, Democratic Republic of Congo. Malaria journal. 2021;20:1-13.

52. Kho S, Qotrunnada L, Leonardo L, Andries B, Wardani PA, Fricot A, et al. Evaluation of splenic accumulation and colocalization of immature reticulocytes and Plasmodium vivax in asymptomatic malaria: A prospective human splenectomy study. PLoS medicine. 2021;18(5):e1003632.

53. Pava Z, Handayuni I, Trianty L, Utami RA, Tirta YK, Puspitasari AM, et al. Passively versus actively detected malaria: similar genetic diversity but different complexity of infection. The American journal of tropical medicine and hygiene. 2017;97(6):1788-96.

54. Buchwald AG, Sorkin JD, Sixpence A, Chimenya M, Damson M, Wilson ML, et al. Association between age and Plasmodium falciparum infection dynamics. American journal of epidemiology. 2019;188(1):169-76.

55. Worku L, Damtie D, Endris M, Getie S, Aemero M. Asymptomatic malaria and associated risk factors among school children in Sanja town, Northwest Ethiopia. International scholarly research notices. 2014;2014.

56. Kwiatkowski D, Nowak M. Periodic and chaotic host-parasite interactions in human malaria. Proceedings of the National Academy of Sciences. 1991;88(12):5111-3.

57. Mason DP, McKENZIE FE. Blood-stage dynamics and clinical implications of mixed Plasmodium vivax–Plasmodium falciparum infections. The American journal of tropical medicine and hygiene. 1999;61(3):367.

58. Richie T. Interactions between malaria parasites infecting the same vertebrate host. Parasitology. 1988;96(3):607-39.

59. Bruce MC, Day KP. Cross-species regulation of malaria parasitaemia in the human host. Current opinion in microbiology. 2002;5(4):431-7.

60. Mayxay M, Pukrittayakamee S, Newton PN, White NJ. Mixed-species malaria infections in humans. Trends in parasitology. 2004;20(5):233-40.

61. Kotepui M, Kotepui KU, Milanez GDJ, Masangkay FR. Plasmodium spp. mixed infection leading to severe malaria: A systematic review and meta-analysis. Scientific Reports. 2020;10(1):1-12.

62. Akala HM, Watson OJ, Mitei KK, Juma DW, Verity R, Ingasia LA, et al. Plasmodium interspecies interactions during a period of increasing prevalence of Plasmodium ovale in symptomatic individuals seeking treatment: an observational study. The Lancet Microbe. 2021;2(4):e141-e50.

63. Achieng AO, Muiruri P, Ingasia LA, Opot BH, Juma DW, Yeda R, et al. Temporal trends in prevalence of Plasmodium falciparum molecular markers selected for by artemether–lumefantrine treatment in pre-ACT and post-ACT parasites in western Kenya. International Journal for Parasitology: Drugs and Drug Resistance. 2015;5(3):92-9.

64. Akala HM, Eyase FL, Cheruiyot AC, Omondi AA, Ogutu BR, Waters NC, et al. Antimalarial drug sensitivity profile of western Kenya Plasmodium falciparum field isolates determined by a SYBR Green I in vitro assay and molecular analysis. The American journal of tropical medicine and hygiene. 2011;85(1):34.

65. Rougemont M, Van Saanen M, Sahli R, Hinrikson HP, Bille J, Jaton K. Detection of four Plasmodium species in blood from humans by 18S rRNA gene subunit-based and species-specific real-time PCR assays. Journal of clinical microbiology. 2004;42(12):5636-43.

66. Kamau E, Alemayehu S, Feghali KC, Juma DW, Blackstone GM, Marion WR, et al. Sample-ready multiplex qPCR assay for detection of malaria. Malaria journal. 2014;13(1):1-8.

67. Calderaro A, Piccolo G, Gorrini C, Montecchini S, Rossi S, Medici MC, et al. A new real-time PCR for the detection of Plasmodium ovale wallikeri. PloS one. 2012;7(10):e48033.
68. Hawadak J, Nana RRD, Singh V. Global trend of Plasmodium malariae and Plasmodium ovale spp. malaria infections in the last two decades (2000–2020): a systematic review and meta-analysis. Parasites & vectors. 2021;14(1):1-14.
69. Chen J, Ma X, Tang J, Xu S, Gu Y, Tang F, et al. Disparate selection of mutations in the dihydrofolate reductase gene (dhfr) of Plasmodium ovale curtisi and P. o. wallikeri in Africa. PLOS Neglected Tropical Diseases. 2022;16(12):e0010977.
70. Joste V, Coppée R, Bailly J, Rakotoarivony Y, Tchokoteu FGT, Achache S, et al. Plasmodium ovale spp dhfr mutations associated with reduced susceptibility to pyrimethamine in sub-Saharan Africa: a retrospective genetic epidemiology and functional study. The Lancet Microbe. 2024;5(7):669-78.
71. Bruce MC, Donnelly CA, Alpers MP, Galinski MR, Barnwell JW, Walliker D, et al. Cross-species interactions between malaria parasites in humans. Science. 2000;287(5454):845-8.
72. Bruce MC, Day KP. Cross-species regulation of Plasmodium parasitemia in semi-immune children from Papua New Guinea. Trends in parasitology. 2003;19(6):271-7.
73. Reece SE, Thompson J. Transformation of the rodent malaria parasite Plasmodium chabaudi and generation of a stable fluorescent line PcGFP CON. Malaria journal. 2008;7(1):1-6.
74. Mideo N. Parasite adaptations to within-host competition. Trends Parasitol. 2009;25(6):261-8.
75. Costa G, Gildenhard M, Eldering M, Lindquist RL, Hauser AE, Sauerwein R, et al. Non-competitive resource exploitation within mosquito shapes within-host malaria infectivity and virulence. Nat Commun. 2018;9(1):3474.
76. Ramiro RS, Pollitt LC, Mideo N, Reece SE. Facilitation through altered resource availability in a mixed-species rodent malaria infection. Ecology letters. 2016;19(9):1041-50.
77. Faye FB, Spiegel A, Tall A, Sokhna C, Fontenille D, Rogier C, et al. Diagnostic criteria and risk factors for Plasmodium ovale malaria. The Journal of infectious diseases. 2002;186(5):690-5.
78. Hemmer CJ, Holst FGE, Kern P, Chiwakata CB, Dietrich M, Reisinger EC. Stronger host response per parasitized erythrocyte in Plasmodium vivax or ovale than in Plasmodium falciparum malaria. Tropical Medicine & International Health. 2006;11(6):817-23.
79. McQueen PG, McKenzie FE. Competition for red blood cells can enhance Plasmodium vivax parasitemia in mixed-species malaria infections. The American journal of tropical medicine and hygiene. 2006;75(1):112.
80. Okafor CN, Finnigan NA. Plasmodium ovale malaria. StatPearls [Internet]: StatPearls Publishing; 2020.
81. Nyarko PB, Claessens A. Understanding host–pathogen–vector interactions with chronic asymptomatic malaria infections. Trends in Parasitology. 2020.

Supporting information

S1. Table 1: Primers and probes used for screening and identification of *Plasmodium* species

S2. Table 2: Metadata among symptomatic and asymptomatic *Plasmodium* infections

S3. Table 3: Overall *Plasmodium* species frequency.

S4. Fig 1: Map showing Kombewa region where Kombewa Sub County hospital is located.

S5. Fig 2: *Plasmodium* species frequency among symptomatic infections by molecular diagnosis: Symptomatic means infections that generate malarial parasitemia alongside symptoms.

S6. Fig 3: *Plasmodium* species frequency among asymptomatic infections by molecular diagnosis: Asymptomatic *Plasmodium* parasite carriers are the infections that generate malarial parasitemia in the absence of fever or other classical symptoms and signs, in otherwise healthy individuals.

S7. Fig 4: Odds of species composition in *Plasmodium* infection presentation

Click here to access/download;Figure;Figure 1_Map of Kenya_Kombewa_11May2026.docx

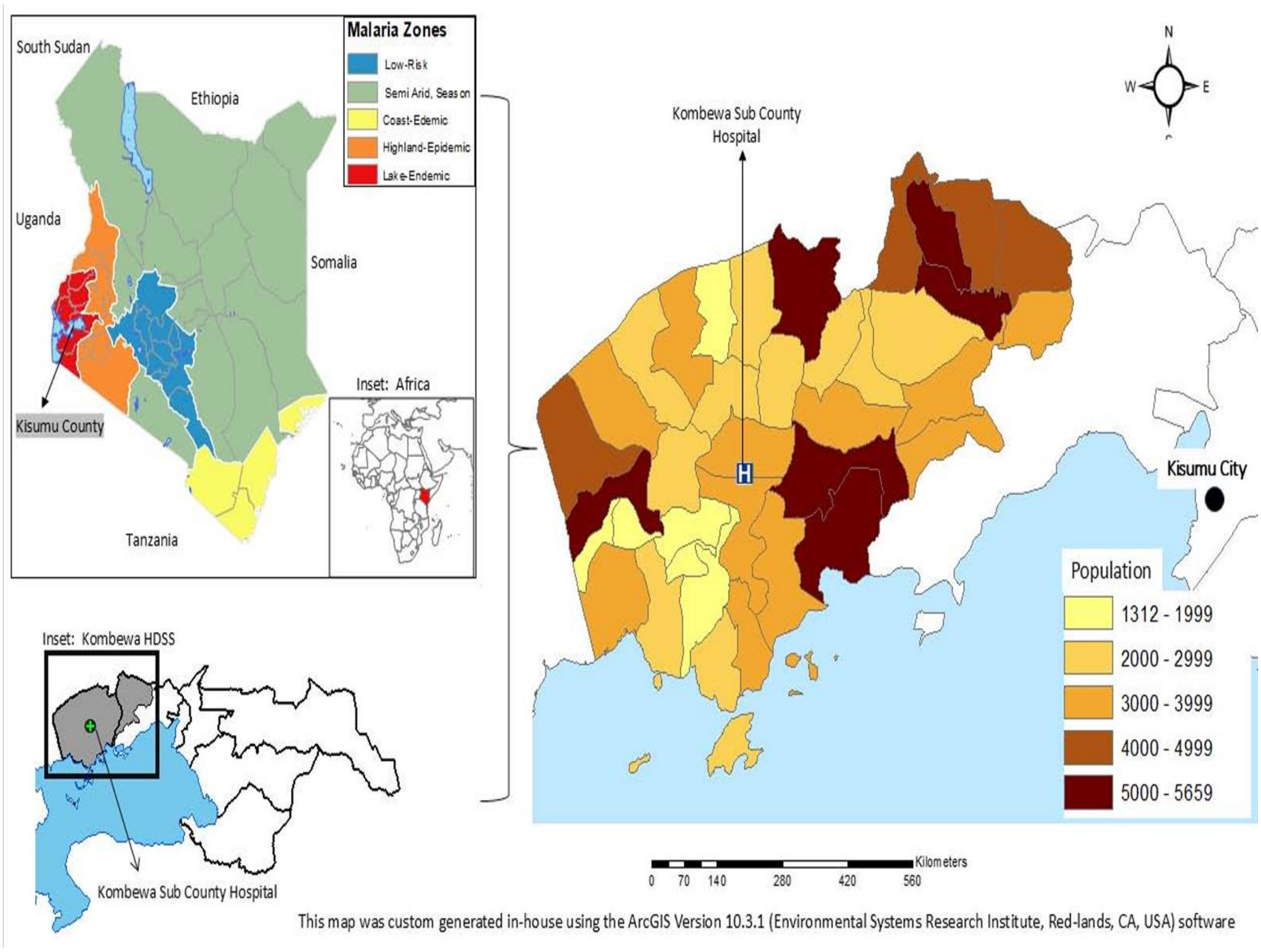

Map data from OpenStreetMap (https://www.openstreetmap.org/copyright). The base maps used in all three images in **Figure 1** were obtained from Open AFRICA.

*This map was custom generated in-house using the ArcGIS Version 10.3.1 (Environmental Systems Research Institute, Red-lands, CA, USA) software.

Fig 1: Map showing Kombewa region where Kombewa Sub County hospital is located.

*Note

Figure

Click here to access/download;Figure;Figure 2_Symptomatic infections_11May2026.docx

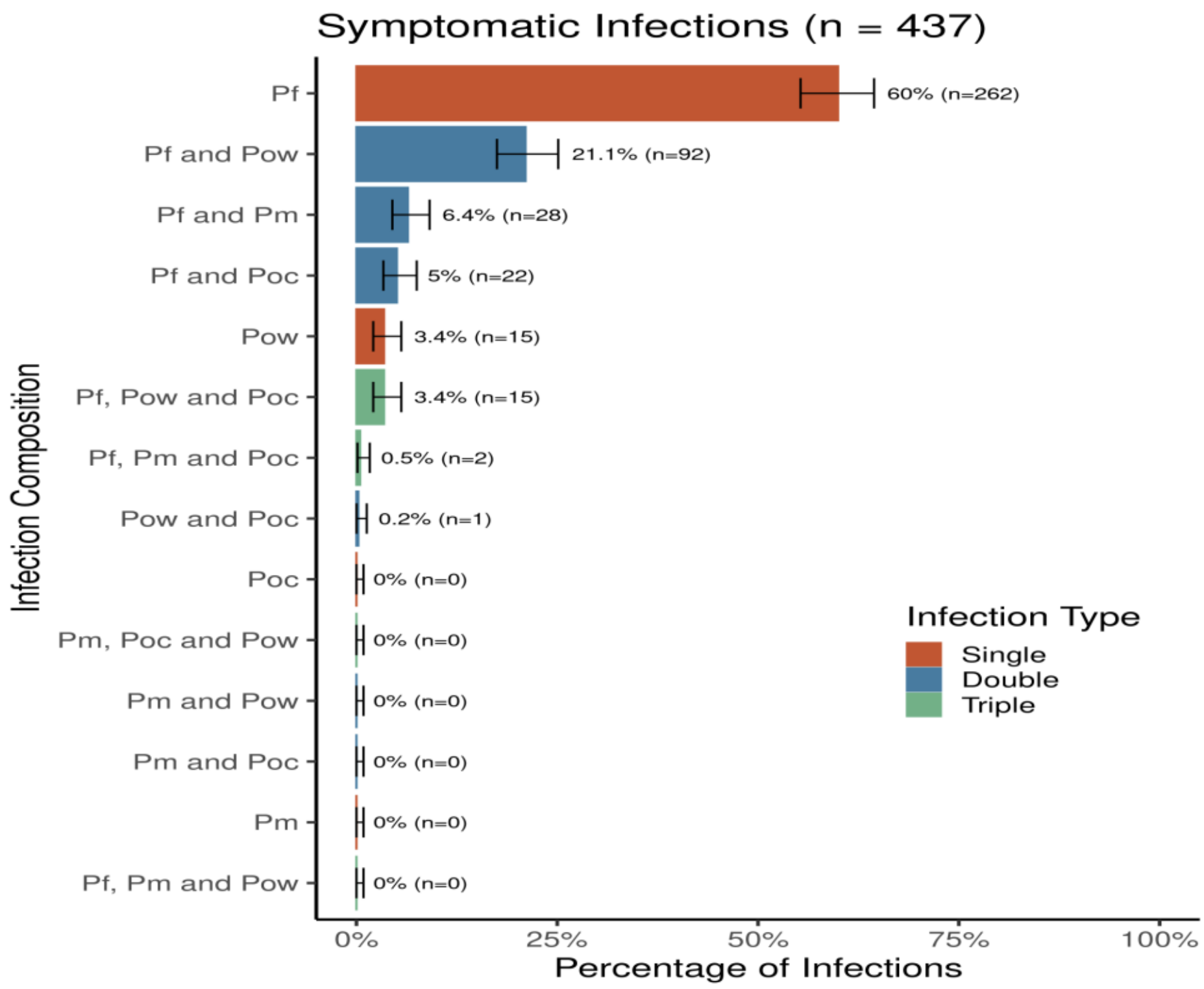

Figure

Click here to access/download;Figure;Figure 3_Asymptomatic infections_11May2026.docx

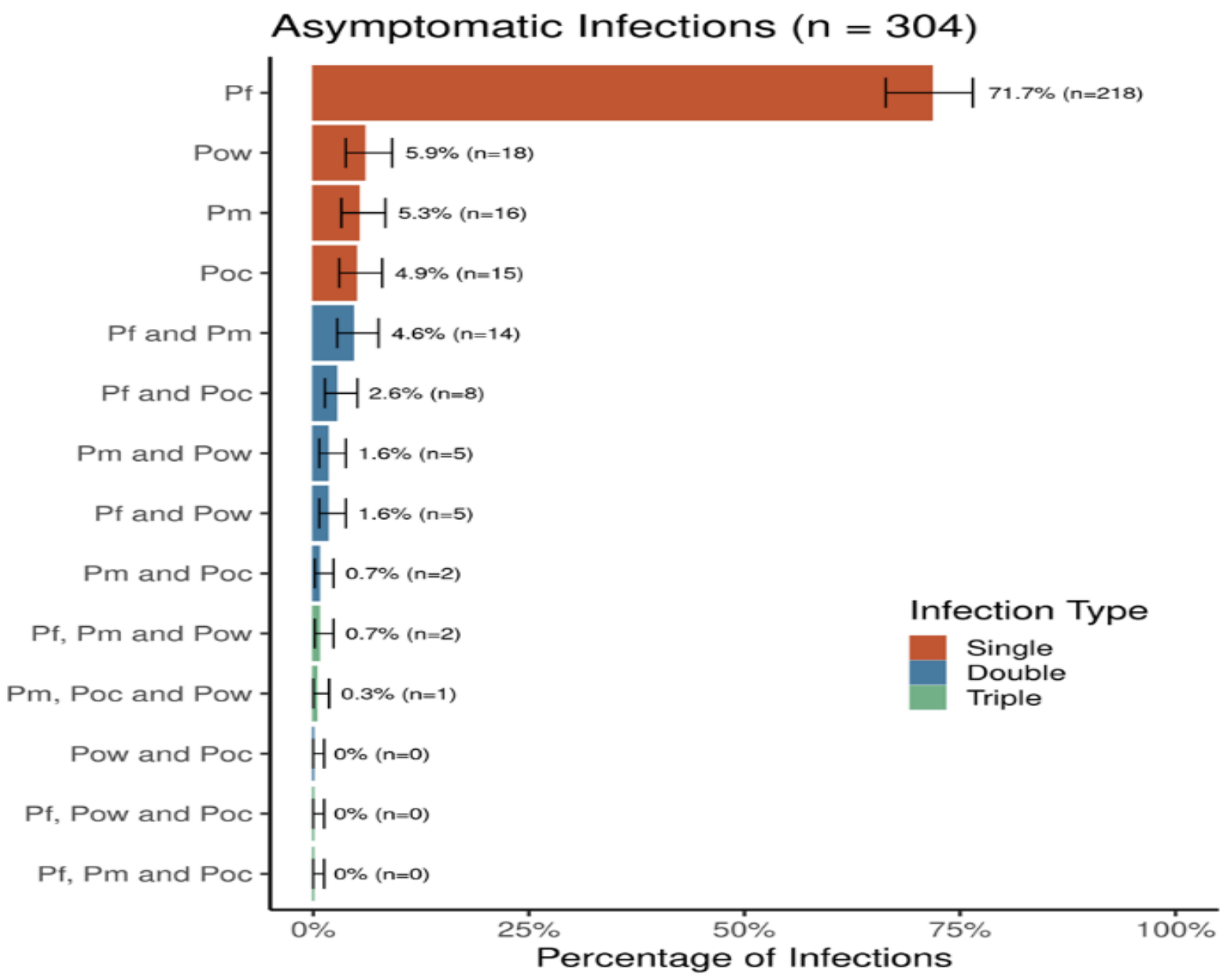

 Click here to access/download;Figure;Figure 4_Odds of species composition_11May2026.docx

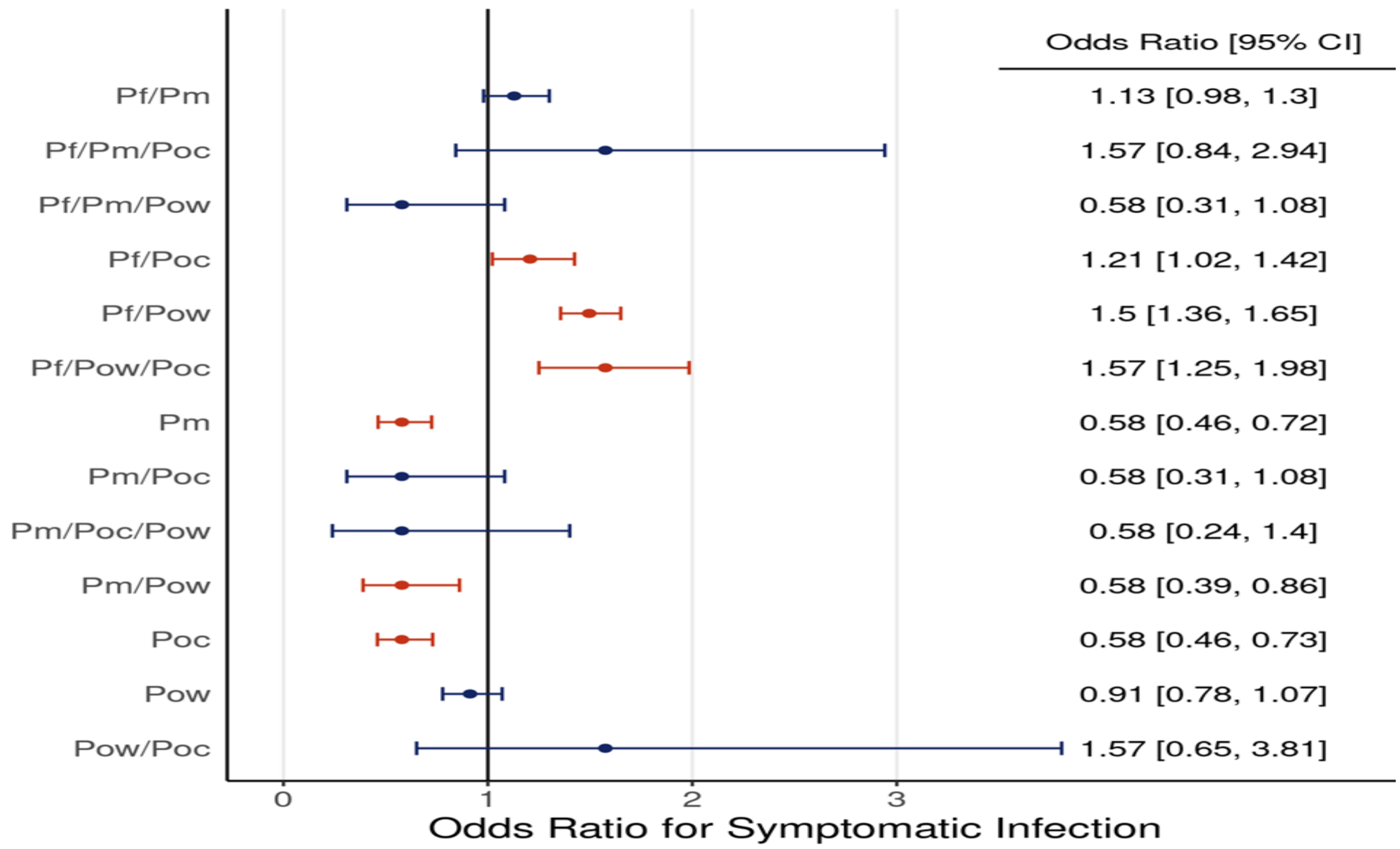

Fig. 4: Odds of *Plasmodium* species composition in infection presentation. The blue represents **no significant association** while the red color represents **significant association.**

Click here to access/download
Supporting Information
WRAIR# 1384 CRR Approval_Study 1.pdf

Click here to access/download
Supporting Information
WRAIR# 2064 CRR Approval_Study 2.pdf

Click here to access/download
Supporting Information
Content Copyright Permission Form_14May2026.pdf

High prevalence of *Plasmodium ovalewallikeri* in co-infections with *Plasmodium falciparum* in symptomatic than asymptomatic *Plasmodium*~~malaria~~ parasite infections~~carriers~~ in Kisumu, Kenya

Formatted: Font: Italic

Jackline Juma[1], Oliver J. Watson[4], Dennis Juma[1], Benjamin Opot[1], Gladys Chemwor[1], Mary Mutahi[3], Raphael Okoth[1], Edwin W. Mwakio[1], Redemptah Yeda[1], Charles Okello[1], Agnes Cheruiyot[1], Duke Omariba[1], Liliana Dayo[9], Hellen A. Ogola[9], Gladys Biwott[1], Charles Adega[1], Fredrick Oluoch[9], Vincent Omondi[9], George Otieno[9], Edwin Kamau[6], Ben Andagalu[1], Peter Sifuna[1], Amanda Roth[7], Sammy Khagayi [8], Eric C. Garges[5], Bernhards Ogutu[2], Hoseah M. Akala[1]*

[1]Department of Emerging and Infectious Diseases, Walter Reed Army Institute of Research-Africa/Centre for Clinical Research, Kenya Medical Research Institute, P. O. Box 54 – 40100, Kisumu, Kenya

[2] Centre for Clinical Research, Kenya Medical Research Institute

[3]The Kenya Medical Research Institute (KEMRI) / Wellcome Trust Research Programme, Centre for Geographic Medicine Research-Coast, Kilifi, Kenya.

[4]Medical Research Council, Centre for Global Infectious Disease Analysis, Department of Infectious Disease Epidemiology, Imperial College London

[5]Walter Reed Army Institute of Research - Africa

[6]Department of Pathology and Laboratory Medicine, Tripler Amy Medical Center, Honolulu, HI 96589, United States of America.

[7]Medical Communications for Combat Casualty Care, 1540 Porter Street, Fort Detrick MD21702.

[8] Centre for Global Health Research, Kenya Medical Research Institute, Kisumu, Kenya

[9]Department of Health County Government of Kisumu, Ministry of Health, Kenya.

*Correspondence to Dr. Hoseah M. Akala, Department of Emerging and Infectious Diseases Walter Reed Army Institute of Research-Africa/Centre for Clinical Research, Kenya Medical Research Institute, P. O. Box 54 – 40100, Kisumu, Kenya. Tel. +254722329845. E-mail: Hoseaakala@yahoo.com ; Hosea.akala@usamru-k.org

Abstract

Plasmodium falciparum (*Pf*), *Plasmodium ovalewallikeri* (*Pow*), *Plasmodium ovalecurtisi* (*Poc*), and *Plasmodium malariae* (*Pm*) coexists in Kenya sympatrically. Some infections progress to classical symptoms of malaria prompting hospital visits, while others remain asymptomatic. There is limited information on whether the species composition status of infections in endemic areas influences progression to either asymptomatic or symptomatic infection. This cross-sectional study determined the composition of *Plasmodium* species among symptomatic versus asymptomatic participants in Kombewa, Kenya.

Between 2015 and 2016, samples from 741 individuals, comprising of 437 from symptomatic at Kombewa Subcounty hospital and 304 from asymptomatic participants from within Kombewa Subcounty hospital's catchment area ~~. Samples~~ were analyzed for *Plasmodium* species composition using real-time qPCR assay targeting the small subunit

ribosomal RNA (ssrRNA) gene. R Studio was used to describe *Plasmodium* species prevalence, and the odds of occurring within given infection presentation.

~~The~~Co-infection analysis revealed 741 samples predominantly contained *Pf* 655/741 (88.3%) followed by *Pow* 153/741 (20.6%), *Pm* 70/741 (9.4%) and *Poc* 64/741 (8.6%); ~~.~~ 12/741 (1.62%) did not ~~amplify for~~amplify any species. A total of~~The 726 contained~~ 535/741 (72.2%) contained single species, and 194/741 (26.18%) multiple species. Species distribution differed significantly, with *Pf* and *Pow* more prevalent in symptomatic cases, while~~and~~ *Pm* and *Poc* were more common in asymptomatic individuals (p=0.000). *Pm* and *Poc* single-species infections occurred among asymptomatic infections only. ~~-~~There were significantly higher odds of multiple species occurrence for infections containing *Pf*/*Pow* 92 (95.8%) among symptomatic than asymptomatic infections (OR 1.5, 95% CI [1.36-1.65], p = 0.000)~~- infections~~. Overall, mixed-species infections involving *Pf* and *Pow* were significantly more common in symptomatic individuals, posing a risk of false diagnosis by *Pf*-Histidine-Rich Protein 2 test. This~~ observation~~ underscores the need for deployment of interventions that target all *Plasmodium* species. These findings on burden across symptomatic vs asymptomatic infections suggests need for determination of the role of species composition in disease progression.~~*Plasmodium falciparum* (*Pf*), *Plasmodium ovalewallikeri* (*Pow*), *Plasmodium ovalecurtisi* (*Poc*), and *Plasmodium malariae* (*Pm*) coexists in Kenya sympatrically. Some infections progress to classical symptoms of malaria prompting hospital visits while others remain asymptomatic. However, there is limited information on whether the species composition status of infections in endemic areas influences progression to either asymptomatic or symptomatic infection. This cross-sectional study determined the composition of *Plasmodium* species among symptomatic versus~~

Formatted: Not Highlight

Formatted: Font: Italic

Formatted: Font: Italic

Formatted: Font: Italic

asymptomatic participants in Kombewa, Kenya. Between the year 2015 and 2016, whole blood samples were obtained from 741 individuals, comprising of 437 from symptomatic at Kombewa Subcounty hospital and 304 from asymptomatic participants whose households were within the in Kombewa Subcounty hospital's catchment area. Each sSamples wereas analyzed for *Plasmodium* species composition using real-time qPCR assay with species-specific primers and probes targeting the small subunit ribosomal RNA (ssrRNA) gene for species identificationto identify the species present in each infection. R Studio was used to describe the prevalence and distribution of *Plasmodium* species and the odds of occurring within given infection presentation. BroadlyThis co-infection study assessed *Plasmodium* species composition and descriptive analyses revealed a total of , the 741 samples mostly contained *Pf* 655/741 (88.3%) followed by *Pow* 153/741 (20.6%), *Pm* 70/741 (9.4%) and *Poc* 64/741 (8.6%) while 12/741 (1.62%) did not amplify for any species, despite amplifying for presence of *Plasmodium* genus. Of these, 535/741 (72.2%) infections contained single species while 194/741 (26.18%) contained multiple species. Comparison of Comparison of *Plasmodium* species frequency among the symptomatic and asymptomatic infections showed significant variation for *Pf* 421/437 (96.3%) and 234/304 (76.9%), *Pow* 123/437 (28.1%) and 30/304 (9.87%), *Pm* 30/437 (6.8%) and 40/304 (13.1%), p=0.000, while *Poc* 40/437 (9.2%) and 24/304 (7.8%), p=0.536 respectively, showed no variation. Furthermore, *Pm/* 16 (5.3%) and *Poc* 15 (4.9%) single species infections occurred among asymptomatic infections only, yet none were among symptomatic individuals, suggesting that these single-species infections are often benign. There were significantly higher odds of multiple species occurrence for infections containing *Pf/Pow* 92 (95.8%) occurring) among symptomatic than asymptomatic (OR 1.5, 95% CI [1.36-1.65], p = 0.000) infectionsdividuals. The high

Formatted: Highlight

Formatted: Highlight

Formatted: Highlight

Formatted: Font: Italic, Highlight

Formatted: Highlight

Formatted: Highlight

Formatted: Highlight

frequency of non-*falciparum* infections poses a risk of missed diagnosis, as histidine rich protein 2 (HRP2) based malaria rapid diagnostic test tailored fors, which are the most widely used, primarily detect *P. falciparum* and may yield false-negative results for other *Plasmodium* species, potentially facilitating continued selective transmission. This observation underscores the need for deployment of interventions that target a broad range of *Plasmodium* species, particularly addressing asymptomatic infections, to support progress toward malaria pre-elimination in the endemic region. Significantly higher prevalence of multiple species infections in symptomatic cases necessitates evaluation of the role of infecting species in disease progression and modulation of treatment-seeking behavior in endemic regions.

Key words

Plasmodium species, symptomatic, asymptomatic

Introduction

Human malaria infection is attributed to six *Plasmodium* species namely, *P.*namely, *P.* *falciparum, P. ovalecurtisi, P. ovalewallikeri, P. malariae, P. viv*and *P.vivax* and *P. knowlesi* (1-3). *P. ovalewallikeri* and *P. ovalecurtisi* are now considered two morphologically identical but distinct species and the names *P. ovalewallikeri* and *P. ovalecurtisi* are preferred over the classical trinominal names. The burden of these species in natural infections varies globally (4) along with their key vectors (5).

P. falciparum is responsible for the most severe form of disease and is the most prevalent in Sub-Saharan Africa (6) where it causes the highest number of fatalities globally (6) *Plasmodium vivax* is the second most dominant species that causes recurring malaria,

with the highest burden in Asia and South America, and in Africa it is mainly in Ethiopia and Sudan (7). The less prevalent species, *P. ovalewallikeri* and *P. ovalecurtisi*, occur primarily in Africa (8, 9). *P. malariae* is predominantly found in South America, Asia, and Africa, and occurs mainly as mixed infections with *P. falciparum* at a lower frequency (10). Consequently, the burden and severity of disease caused by non-falciparum species, which are generally presumed to be milder than those associated with *P. falciparum*, remain poorly characterized in endemic human populations (11-13).

The World Health Organization recommends that all suspected malaria cases receive an accurate parasitological test to confirm infection before antimalarial treatment is administered (14-20). However, the parasitological diagnosis methods (21, 22) are less accurate in the diagnosis of low parasitemia (23) and distinguishing infecting species (24) in asymptomatic individuals (25) and therefore likely to underestimate the burden and complexity of infection in the population (26, 27). These methods, therefore, have limited utility in pre-elimination settings. The innovation of molecular diagnostic methods capable of detecting subclinical and submicroscopic *Plasmodium* infections (28, 29), which serve as reservoirs for ongoing transmission (30), is emerging as an important surveillance tool for malaria elimination strategies (31-34).

In Kenya, *P. falciparum* infection occurs predominantly as mono-infection or sympatrically with *P. malariae* and *P. ovalewallikeri*, and *P. ovalecurtisi*. This co-endemicity of species leads to the occurrence of blood-stage mixed *Plasmodium* species infections (35), which are mostly acquired from separate inoculations when a recently acquired infection of one species might supervene a chronic infection with another (36) or simultaneous inoculation of the sporozoites of two or more species from a doubly infected *Anopheles*

mosquito (36-38). Studies have highlighted variations in the clinical presentation of disease per species relative to the unique parasite biology of each malaria species. Evaluating the species composition of infections among symptomatic individuals seeking care at health facilities and asymptomatic individuals within households is essential for understanding and addressing the rising prevalence of non-*Plasmodium falciparum* species in the context of improved access to accurate diagnosis and timely treatment, which are key for transitioning regions with declining malaria to pre-elimination.

Clinical symptoms of malaria typically develop between 7 and 85 days after initial infection, depending on the infecting *Plasmodium* species (39-43). Parasitemia may also persist in susceptible individuals for up to eight years before the onset of symptoms (42-45). Initial symptoms of malaria may include a high temperature of 38°C and above, muscle pains, and generally feeling unwell, often characterized by recurrent fever and chills (42), which often trigger a treatment-seeking response (20) once parasite replication becomes synchronous. In some cases, the infection may generate malarial parasitemia in the absence of fever or other classical symptoms and signs, in otherwise healthy individuals, therefore regarded as asymptomatic *Plasmodium*~~malaria~~ parasite carriers (21, 46, 47). Asymptomatic *Plasmodium* infections~~malaria~~ accounts for up to 60% of infections (48-51) in some regions. It has been suggested that infections remain asymptomatic due to sustained submicroscopic peripheral parasitemia (52, 53), older age, and therefore partial immunity of the infected individual (54, 55) and varying malaria pyrogenic threshold (56, 57). In a 2011 study, the complex interactions between infecting *Plasmodium* species (56, 58) were shown to affect the disease outcome such as the occurrence of symptoms (59) and transmissibility, bringing in to focus the role of these interactions in either limiting or promoting disease progression.

Formatted: Font: Italic

Formatted: Font: Italic

In a region with natural infections comprising more than one *Plasmodium* species (60, 61), there is uncertainty about whether infection with multiple species reduces or promotes the incidence of symptoms in the infected individual as a means of maintaining transmissibility. Our previous study showed the prevalence of *Plasmodium* species co-infections changes over time, among symptomatic individuals seeking for treatment at selected hospitals across Kenya (62), but did not describe the burden among asymptomatic infections. Given the varying disease progression among *Plasmodium* species such as longer period of case management for relapsing species, estimating of the burden of species in the population is useful in informing choice of intervention needed to lower malaria in line with the WHO goal that targets 90% reduction of malaria by 2030. This study aimed to estimate the composition of *Plasmodium* parasite species among symptomatic versus asymptomatic infections~~ndividuals~~ in Kisumu County in western Kenya.

Materials and methods

Ethical considerations

Ethical approval was obtained from Kenya Medical Research Institute (KEMRI), Scientific and Ethics Review Unit (SERU), and Walter Reed Army Institute of Research (WRAIR) institutional review boards (protocol numbers: KEMRI 1330/WRAIR 1384 and KEMRI 2721/ WRAIR 2064. A written informed consent /assent was obtained from each of the study participants. The confidentiality of information obtained from study participants was assured by using unique identifiers.

Study site and sample collection

Kombewa Health and Demographic Surveillance System (HDSS) grew out of the Kombewa Clinical Research Centre in 2007 and has since established itself as a platform for conducting nested studies and local disease surveillance, according to a study by Sifuna and co-workers. This study was conducted within Kombewa HDSS, which forms the main catchment of the malaria cases for the Kombewa Sub-County hospital, an area that is categorized as a rural Lake Endemic Zone with perennial and intense malaria transmission as per the National Malaria Control Programme 2016 (NMCP).

This sub-study analysed *Plasmodium*-positive samples obtained from two studies that were conducted concurrently between 2015 and 2016. The first study was entitled "Epidemiology of malaria and drug sensitivity patterns in Kenya", which commenced from 1st September 2015 to 1st June 2016. The second study was entitled, "Baseline Epidemiology of Malaria Gametocyte Carriage and Transmission Dynamics in a Holoendemic Transmission Setting in Kisumu County, Western Kenya", which commenced on 18th March 2015 to 1st February 2016.

The first study enrolled symptomatic participants aged six months and above presenting at Kombewa Sub-County Hospital with clinical features suggestive of malaria, including fever (≥37.5 °C) or a history of fever within the preceding 48 hours accompanied by symptoms such as chills, headache, malaise, or sweating. Participants who were clinically stable, consistent with uncomplicated malaria, and who tested positive for malaria by rapid diagnostic test (RDT) and/or microscopy were eligible for inclusion. Under this study, these individuals were referred to as symptomatic malaria participants. These individuals were enrolled into the study upon providing written informed consent/assent. Each participant

provided 2-3 ml of whole blood for malaria parasite species genotyping alongside other endpoints of the main study reported elsewhere (63, 64). All *Plasmodium Plasmodium* positive cases were treated with artemether-lumefantrine (AL) (Coartem®), as per the Kenya Ministry of Health's recommended case management guidelines for uncomplicated malaria. The healthcare workers administered and observed the taking of the first dose of AL 20mg/120mg tablets.

The second study enrolled asymptomatic participants and sought to estimate the level of gametocyte carriage in the study population by PCR. These comprised individuals of all ages, without clinical features suggestive of malaria, such as fever (≥37.5 °C) or history of fever within the previous 48 hours, and absence of symptoms such as chills, headache, malaise, or sweating. Under this study, these individuals were referred to as asymptomatic malaria participants. Members of selected households within Kisumu West sub-County, which is the main catchment for the Kombewa sub-County hospital (**Fig.1 Map of Kombewa**), willing to be tested for the presence of malaria parasites were enrolled into the study upon providing written informed consent/assent. During this household visit, vital signs were measured to rule out symptoms of malaria. Based on these readouts, each of the otherwise healthy participants provided 4 ml of whole blood for the diagnosis of malaria. Each sample was tested within 24 hours, and all participants found to have *Plasmodium* genus infection were treated with the attending healthcare worker administering the first dose of AL 20mg/12mg tablets.

*****This map was custom generated in-house using the ArcGIS Version 10.3.1 (Environmental Systems Research Institute, Red-lands, CA, USA) software.

Fig 1: Map showing Kombewa region where Kombewa sub-County hospital is located.

*Note

Molecular assay

Nucleic acids extraction

The parasite nucleic acids were extracted from blood samples obtained from individuals enrolled in the two studies using a commercial extraction kit (QIAamp DNA Blood Mini Kit, Qiagen) as recommended by the manufacturer's instructions.

Plasmodium genus diagnosis by molecular methods

The extracted nucleic acids were analysed for malaria diagnosis using *Plasmodium* genus-specific qPCR assay as previously described (65, 66). PCR amplified the *Plasmodium* genus 18S ribosomal ribonucleic acid (rRNA) gene using primers listed in **Table 1**. The RN*ase* P gene, which is a human housekeeping gene, was used as an extraction and qPCR assay control.

Detection of species composition of infections

All samples that were diagnosed as positive for the *Plasmodium* genus were further characterized for species composition as described by (65, 66) using a separate set of primers indicated in **Table 1.** The assays for characterization of *Plasmodium falciparum* and *P. malariae* had similar PCR reaction components and conditions as the genus-specific qPCR assay except for the primers used. Genus-specific primers were replaced respectively by the species-specific primers (28, 67) **(Table 1**). Each experiment included at least one reaction mixture without DNA as a negative control.

Table 1: Primers and probes used for screening and identification of *Plasmodium* species

Primers Probes	Sequence 5'-3'	Target genus/species
PLU F	GCTCTTTCTTGATTTCTTGGATG	*Plasmodium* spp.
PLU R	AGCAGGTTAAGATCTCGTTCG	*Plasmodium* spp.
PLU P	ATGGCCGTTTTTAGTTCGTG	*Plasmodium* spp.
RNaSP F	TGTTTGCAGATTTGGACCTGC	Human RNase p
RNaSeP R	AATAGCCAAGGTGGAGCGGCT	Human RNase p
RNaseP P	TGCGCGGACTTGTGGA	Human RNase p
FAL F	ATTGCTTTTGAGAGGTTTTGTTACTT	*P. falciparum*
FAL R	GCTGAGTATTCAAACACAATGAACTCAA	*P. falciparum*
FAL P	CATAACAGACGGGTAGTCAT	*P. falciparum*
MAL F	GCATGGAATTTTGTTACTTTGA	*P. malariae*
MAL R	ATGCCTGTAGTATTCAACACAGAAAC	*P. malariae*
MAL P	TGTTCAAAGCAAACAGTTAAAACA	*P. malariae*
OVA F	TTTTGAAGAATACATTAGGATACAATTAATG	*P. ovale curtisi*
OVA R	CATGCTTCCTCTAAGAAGCTTTACAAT	*P. ovale*
OVA-V F	TTTTGAAGAATATATTAGGATACATTATAG	*P. ovale Wallikeri*
OVA-V R	CATCGTTCCTCTAAGAAGCTTTACAAT	*P. ovale Wallikeri*
OVA P	CCTTTTCCCTATTCTACTTAATTCGCAATTCATG	*P. ovale curtisi*
OVA-V P	CCTTTTCCCTACTTAATTCGCTATTCATTG	*P. ovale Wallikeri*

Data management and statistical analysis

Descriptive analyses were used to describe categorical data comprising age categories, gender, and qPCR species results summarized as frequencies and proportions ~~Data was captured in excel where data cleaning and transformation was conducted. Categorical data comprising age categories, gender, and qPCR species results were summarized as frequencies and proportions~~. Continuous variables, including age in years, were first assessed for normality using the Shapiro–Wilk test and then expressed as median together with their corresponding interquartile ranges (IQR) when non-normal distributions were observed. The geometric mean was used as a measure of central tendency for parasite density.

Descriptive statistics were employed to illustrate the burden of *Plasmodium* species among the participants with positive infections. For inferential statistics, proportions were

Formatted: Indent: First line: 0.5"

Formatted: Justified, Indent: First line: 0.5"

compared using the Chi-square test~~χ2 test or Fisher's exact test,~~ as appropriate for the categorical data. Wilcoxon rank-sum test was used for non-parametric continuous variables. Logistic regression analysis was also conducted to assess the varying odds of *Plasmodium* species composition. All analyses were performed using R Studio version 4.5.1. Sta~~All~~tistical analyses ~~were performed~~were performed at the 5% significance level and the corresponding 95% confidence interval (CI).~~ using R Studio version 4.5.~~ The critical significance level was set at $p \leq 0.05$.~~1.~~

Results

A total of 741 samples comprising 437 (58.9%) of~~from~~ symptomatic participants and 304 (41.0%) of~~from~~ the asymptomatic participants were analyzed. The description of the study population is summarized in **Table 2**. Metadata were available for 733/741 participants~~individuals~~ though species analyses data ~~were~~was available for all the 741 samples. The median ages for all age categories and number of participants~~individuals~~ per age category varied significantly across the two infections presentations χ^2 test p=0.000 (**Table 2**). The categories of less than 5 years were highest in symptomatic infections~~participants~~, 258 (79.4%) while over 18 years were highest in asymptomatic infections~~participants~~ 81 (66.9%) (**Table 2**). Wilcoxon rank-sum test revealed a significantly higher age among the asymptomatic, median 9 years; IQR, 5-20.5 than symptomatic 4 years; IQR, 3-6, p=0.000, r=0.38. Gender was comparable between the symptomatic and asymptomatic infections. The geometric mean of parasite density per microliter of blood was significantly higher among the symptomatic (47504.83/ul, 95%CI 39131.25-57670.26) than asymptomatic infections (784.11/ul, 95%CI 784.11-1667.1) ~~infections~~ (**Table 2)**.

Formatted: Indent: First line: 0.5"

Table 2: Metadata among symptomatic and asymptomatic *Plasmodium* ~~malaria~~ infections

Formatted: Font: Italic

Variable	Symptomatic	Asymptomatic	p value
**Age years:**			
**median (IQR)**	4 (3-6)	9 (5-21)	0.000
**Age category:**			
**<5 years (n)%**	258 (79.38)	67 (20.62)	0.000
**5-18 years (n)%**	131 (45.64)	156 (54.36)	
**>18 years (n)%**	40 (33.06)	81 (66.94)	

**Gender:**			
**Male (n)%**	195 (60.56)	127 (39.44)	0.323
**Female (n)%**	234 (56.93)	177 (43.07)	
**Parasite density:**			
**Geometric mean**	47504.83	1143.33	0.000
**(95% CI)**	(39131.25-57670.26)	(784.11-1667.107)	

Table 2: Metadata for symptomatic and asymptomatic *Plasmodium* infections.

Plasmodium co-infections analyses were conducted and the s~~S~~pecies composition analyses detected the presence of single *Plasmodium* species in most infections and presence of two or three or four *Plasmodium* species in a subset of infections. Therefore, ~~the~~ computing the raw frequencies of any detection of *Plasmodium* species yielded varying percentages that cumulatively exceeded 100%, consistent with the expected classification of infections containing multiple species under two or more species profiles. On this basis, *Plasmodium* species detection by qPCR assay showed that,~~ of the 741 infections~~ 655/741 (88.3%) of the infections~~,~~ contained *P. falciparum*, 153/741 (20.7%) *P. ovalewallikeri*, 70/741 (9.4%)~~,~~ *P. malariae*, and 64/741 (8.7%) *P. ovalecurtisi* without consideration of the number of species per ~~infection~~infection (**Table 3**).~~.~~ However, 12/741 (1.6%) amplifying for presence of *Plasmodium* genus did not amplify for any species.

~~(**Table 3**).~~ Subsequent analysis with a focus on the presence or absence of more than one species per infection revealed that a total of 535/741 (72.2%) samples had single species while 194/741 (26.1%) had multiple species infections. *P. falciparum* was the most frequent single-species infection occurring in 470/535~~741~~ (87.9~~63.4~~%) infections, followed *by P. ovalewallikeri* in 34/535~~741~~ (6.4~~4.5~~%), *P. malariae* in 18/535~~741~~ (3.4~~2.4~~%), and *P. ovalecurtisi* in 13/535~~741~~ (2.4~~1.7~~%). Notably, all the *P. malariae* and *P. ovalecurtisi* single-species infections were~~ only~~ detected among asymptomatic infections only. Multiple species

Formatted: Font: Not Bold
Formatted: Font: Not Bold
Formatted: Font: Italic
Formatted: Font: Not Bold
Formatted: Font: Italic, Highlight
Formatted: Indent: First line: 0.5"
Formatted: Highlight
Formatted: Highlight
Formatted: Highlight
Formatted: Highlight
Formatted: Highlight

infections included *Pf/Pow* 96/194 (49.5%), being dominant followed by *Pf/Pm* 41/194 (21.1%), *Pf/Poc* 30/194 (15.5), *Pf/Pow/Poc* 15/194 (7.7%), *Pm/Pow* 5/194 (2.6%), *Pf/Pm/Poc* 2/194 (1.0%), *Pm/Poc* 2/194 (1.0%), *Pf/Pm/Pow* 1/194 (0.5%), *Pm/Poc/Pow* 1/194 (0.5%), *Pow/Poc* 1/194 (0.5%)

Table 3: Overall *Plasmodium* species frequency

Table 3: Overall *Plasmodium* species frequency detected in the samples

Formatted: Font: Italic, Highlight
Formatted: Highlight
Formatted: Font: Italic, Highlight
Formatted: Highlight
Formatted: Font: Italic, Highlight
Formatted: Highlight
Formatted: Font: Italic, Highlight
Formatted: Highlight
Formatted: Font: Italic, Highlight
Formatted: Highlight
Formatted: Highlight
Formatted: Font: Italic, Highlight
Formatted: Highlight
Formatted: Font: Italic, Highlight
Formatted: Highlight
Formatted: Font: Italic, Highlight
Formatted: Highlight
Formatted: Font: Italic, Highlight
Formatted: Highlight
Formatted: Font: Italic, Highlight
Formatted: Highlight
Formatted: Font: Italic, Highlight
Formatted: Highlight
Formatted: Font: Italic, Highlight
Formatted: Highlight
Formatted: Font: Italic, Highlight
Formatted: Highlight
Formatted: Indent: First line: 0.5"

~~**Table 3: Overall *Plasmodium* species frequency**~~

Formatted: Space After: 0 pt, Line spacing: single

Formatted Table

Formatted: Font: 12 pt

Symptomatic infections

Assessment of frequency of single *Plasmodium* species showed that *P. falciparum* 262/437 (60%) and *P.* ~~*ovalewallikeri*~~*ovale wallikeri* 15/437 (3.4%) were the only single species detected among the symptomatic infections (**Fig. 2**). The symptomatic individuals harbored multiple *Plasmodium* species, comprising 2 species; *PfPow* 92/437 (21.1%), *PfPm* 28/437 (6.4%), *PfPoc* 22/437 (5%), *PowPoc* 1/437 (0.2%) three species; *PfPowPoc* 15/437 (3.4%), *PfPmPoc* 2/437 (0.5%) (**Fig. 2**). Further, neither *P. ovalecurtisi* nor *P. malariae* occurred as a single species infection among symptomatic participants~~individuals~~ during this study period (**Fig. 2**).

Fig 2:~~.~~ *Plasmodium* single species and mixed species frequency among symptomatic infections ~~participants~~ by molecular diagnosis.~~:~~ Symptomatic infections refer to infections that generate malarial parasitemia alongside symptoms.

Asymptomatic infections

Single *Plasmodium* species infections included *P. falciparum* detected in 218/304 (71.7%) of the asymptomatic infections as the most prevalent species, followed by *P. ovalewallikeri* 18/304 (5.9%), *P. malariae* 16/304 (5.3%), and *P. ovalecurtisi* 15/304 (4.9%), respectively (**Fig 3**). Multiple species infections comprised double; *PfPm* 14/304 (4.6%), *PfPoc* 8/304 (2.6%), *PmPow* 5/304 (1.6%), *PfPow* 5/304 (1.6%), *PmPoc* 2/304 (0.7%), or triple *PfPmPow* 2/304 (0.7%) and *PmPocPow* 1/304 (0.3%) infections (**Fig 3**).

Fig 3:~~.~~ *Plasmodium* mixed and single species frequency among asymptomatic infections~~participants~~ by molecular diagnosis.~~:~~ Asymptomatic *Plasmodium*~~malaria~~ parasite carriers are the infections that generate malarial parasitemia in the absence of fever or other classical symptoms and signs, in otherwise healthy individuals.

The *P. falciparum* single-species infections were more prevalent in asymptomatic than symptomatic individuals **(Figs. 2 & 3**). Multiple species were more likely to be symptomatic ~~infections~~ than asymptomatic infections. Assessment of the role of infection composition in disease presentation showed that *P. falciparum* with *P. ovalewallikeri*; *P. falciparum* with *P. ovalecurtisi*, and *P. falciparum*, *P. ovalewallikeri* with *P. ovalecurtisi* mixed-species infections had higher odds of progressing to symptomatic infection than remaining asymptomatic (**Fig. 4**). The mixed species had increased odds of symptomatic infection (**Fig. 4**).

Formatted: Font: 12 pt

Formatted: Font: Italic

Formatted: Font: 12 pt

Fig. 4: Odds of *Plasmodium* species composition in infection presentation. The blue represents no significant association, while the red color represents a significant association.

Formatted: Justified

Discussion

This study concurrently compared the prevalence of *Plasmodium* species among symptomatic individuals seeking treatment at the Kombewa Sub-County hospital versus asymptomatic individuals in households within the same geographic location that doubles up as the catchment of the hospital using qPCR. About 13% and 26% prevalence of multiple species was detected among asymptomatic and symptomatic infections, respectively. Studies in West and Central Africa focusing on school-going children have also shown a higher burden of non-*falciparum* species in asymptomatic *Plasmodium*~~malaria~~ parasite carriers (50, 51). This finding is contrary to a recent meta-analysis showing no difference in the prevalence of either *P. malariae* or *P. ovale spp* between symptomatic and asymptomatic individuals (68). Additionally, large-scale studies of non-*falciparum* parasites report conflicting results for those from West Africa (69) versus those from central or East Africa (70). These varying observations underscore the peculiarity of *Plasmodium* infection presentation across different disease endemicities and are evocative of the need for characterizing species composition in diverse disease geo-ecotypes for effective targeting.

The occurrence of *Plasmodium* parasitemia in the absence of classical malaria symptoms, and the mechanisms that influence the transition from an asymptomatic infection to symptomatic infection, remain subjects of ongoing debate. A prospective study by Chen and coworkers showed that some individuals harboring *Plasmodium* parasites can either remain healthy for an unlimited amount of time and referred to as asymptomatic (21) while others progress to disease with malaria symptoms within 7-85 days and seek for the treatment depending on various factors, including the species composition of the infection (47, 58, 71,

Formatted: Font: Italic

72). This study detected a higher frequency of mixed species infection among symptomatic than asymptomatic individuals, an observation that appears to align with the theory that harboring multiple species potentiates the transition of infections to the symptomatic phase.

Studies on inter-parasite interactions within human hosts suggest that parasites often interact with unrelated competitors within the host (73) and use this mechanism~~information~~ to prioritize the resource allocation framework, opting to increase replication to occupy most of the red blood cells at the expense of the development of sexual stages as one of the exclusion strategies. The changes in resource utilization resulting from the presence of the additional species have been associated with modulation of the host response (59, 74-76) toward either the persistence of the asymptomatic phase of the infection (77) or progression to symptomatic disease in the presence of virulence-enhancing species mixtures (78). The detection of a higher frequency of multiple species infections among the symptomatic than asymptomatic, with a reciprocal higher frequency of single species infections in asymptomatic than symptomatic, by this study appears to suggest that infection with more than one species is likely to progress to a more symptomatic state than single-species infections as reported elsewhere (26, 27). It was, however, surprising that asymptomatic individuals harbored more diverse species profiles than symptomatic individuals based on PCR-based diagnosis.

The composition of mixed species infections varied significantly between the symptomatic and asymptomatic infections. For example, there was a ten-fold higher prevalence of co-infections containing *P. falciparum* and *P. ovalewallikeri* among the symptomatic individuals than among the asymptomatic individuals. A similar pattern of frequency was observed among infections containing *P. falciparum, P. ovalewallikeri,* plus

P. ovalecurtisi, contrary to *P. falciparum/P. ovalecurtisi, or P. falciparum/P. malariae* or either of these species in single-species infections, an observation that may suggest between-species interactions.

Evidence from malaria ecology indicates that the acquisition of an additional *Plasmodium* species within a host can modify the within-host ecological environment by introducing competition for limited resources such as red blood cells. For example, in mixed-species infections involving human malaria parasites, including *P. falciparum* and *P. vivax*, parasites may compete for host erythrocytes and other resources, which can alter parasite replication dynamics and suppress the density of competing species. These competitive interactions may consequently influence parasite fitness, disease outcomes, and transmission potential (79). Parasite biology studies show that *P. falciparum* attacks all ages of red blood cells, hence it has been hypothesized to be a "generalist" while *P. ovale* and *P. malariae* attack younger and older red blood cells, respectively, and are hypothesized to be specialists (77, 78, 80).

Studies show that in mixed-species infections, ~~in~~ the presence of an RBC *Plasmodium* species regarded as "generalists" enhances the density of RBC specialists (76). A study monitoring blood levels of tumor necrosis factor-alpha (TNF-alpha), lactate dehydrogenase (LDH), Thrombin-antithrombin III (TAT) and elastase as drivers of malaria symptoms through modulation of host immune response among uncomplicated malaria was conducted. The study further noted that these parameters were higher per parasitized *P. ovale* erythrocyte levels than *P. falciparum*, with the former attaining full-strength host response at lower parasitemia than the latter (78). Observation of significantly higher frequency *P. falciparum* and *P. ovalewallikeri* among naturally acquired symptomatic than asymptomatic ~~of~~ infection

Formatted: Highlight

Formatted: Highlight

Formatted: Highlight

by this study suggests that parasitemia increases in *P. ovalewallikeri,* a specialist, given the presence of *P. falciparum* a generalist, could be contributing to elevated immune response leading to the occurrence ~~of symptoms~~of symptoms among these infections. This phenomenon, which is absent in *P. ovalecurtisi* infections, highlights the biological differences between the two *P. ovale* species. Notably, *P. ovalewallikeri* single-species infections were detected among symptomatic as well as asymptomatic infections while *P. ovalecurtisi* alone was not detected among the symptomatic, suggesting that *P. ovalewallikeri* alone causes symptoms more frequently than *P. ovalecurtisi.*

Single-species infections of *P. falciparum, P. malariae, or P. ovalecurtisi* were mostly detected among asymptomatic individuals than symptomatic. Notably, *P. ovalecurtisi* single-species infections were detected in 4.8% of the asymptomatic infections while none was detected in symptomatic infections. Additionally, infections comprising *P. malariae*/*P. ovalecurtisi* or *P. malariae*/*P. ovalewallikeri* were detected in asymptomatic infections at less than 2% frequency while none were detected among symptomatic infections. *P. malariae* co-infections with *P. ovalecurtisi* or *P. ovalewallikeri* have been associated with low pyrogenic thresholds, occurring in less than 2% of the symptomatic cases (77). This infection characteristic is a challenge for malaria elimination since the chronically infected individual would remain obscure to healthcare facilities while still capable of transmitting the parasite to mosquitoes and therefore sustaining transmission to humans. Such infections have been proposed as the last battlefield for malaria elimination (81) since they will also remain undetected by the histidine-rich protein based malaria rapid diagnostic test that are expedient for population-based strategies of bringing down diseases.

This study has some limitations. The study depended on the vital signs of the participants at the time of enrolment such fever ≥37.5 °C or a history of fever within the preceding 48 hours accompanied by symptoms such as chills, headache, malaise, or sweating, to be categorized as symptomatic participants, while those without these signs were categorized as asymptomatic. Because of the study design, it was difficult to determine when, specifically, the time-point in the previous period preceding enrolment day, at which either the asymptomatic or symptomatic individuals acquired the infection. Consequently, it was not possible to establish whether individuals with asymptomatic infections would subsequently develop symptoms as parasite densities increased to the pyrogenic threshold, or whether the infections in these individuals were chronic and would remain asymptomatic. It was again not possible to ascertain which of the infecting species in individuals harbouring multiple species infections came first and which one came later. This, therefore, made it harder to tell whether there was protectiveness proffered by some species or immune silencing by other species to delay symptoms, or associate entry of a given species with the onset of symptoms. Further, the study was not able to filter out and show within-host response to varying species composition of infections or between-species interactions within each of the mixed infection profiles to filter out specific parasite species-specific biology that infer implication to the progression of disease or epidemiology of each of the species versus presently deployed malaria interventions. Finally, host immunity was not characterized in this study because it ~~as it~~ was not one of the endpoints for the study~~of this study~~.

Conclusion

These findings in a malaria-endemic zone suggest that intervention strategies need to consider the species composition of asymptomatic *Plasmodium* parasite~~malaria~~ infections to progress the region to the elimination phase. The ten-fold higher frequency of *P. ovalewallikeri* species among the symptomatic than asymptomatic infections and higher *P. falciparum* single-species infections in the asymptomatic than symptomatic appears to associate this infection composition with higher likelihood of symptoms. Interventions need to focus on the mixed infections asymptomatic *Plasmodium*~~malaria~~ parasite carriers since these two serves as silent reservoirs for transmission and are a lynchpin to malaria elimination in a given region.

Formatted: Font: Italic

Formatted: Font: Italic

Acknowledgments

Material has been reviewed by the Walter Reed Army Institute of Research. There is no objection to its presentation and/or publication. The opinions or assertions contained herein are the private views of the author, and are not to be construed as official, or as reflecting true views of the Department of the Army or the Department of Defense. The investigators have adhered to the policies for protection of human subjects as prescribed in AR 70–25. We thank the individuals who participated in this study for providing these invaluable samples and time to generate these findings. We also thank the County Government of Kisumu and the Ministry of Health, Kenya, for giving us access to these samples. We thank the Commander, Walter Reed Army Institute of Research - Africa, COL. Lacy Shannon, Director, Walter Reed Army Institute of Research - Africa, Kisumu Field Stations, COL. Gerald Keller, Deputy Director General, Center for Clinical Research, Kenya Medical Research Institute, Kenya, for supporting this study and giving their permission to

Formatted: Indent: First line: 0.5"

publish these data. We also thank all clinical staff at all the participating hospitals for their assistance.

Data Availability

All relevant data are within the paper and its Supporting Information files. Any other additional information can be availed upon requesting the corresponding author.

Formatted: Font: 18 pt

Formatted: Heading 1, Left, Line spacing: single

Formatted: Font: 18 pt, Not Bold

Formatted: Font: Times New Roman, 12 pt

Formatted: Font: Times New Roman, 12 pt

Formatted: Font: Times New Roman, 12 pt

Formatted: Font: 12 pt

References

1. Ansari HR, Templeton TJ, Subudhi AK, Ramaprasad A, Tang J, Lu F, et al. Genome-scale comparison of expanded gene families in Plasmodium ovale wallikeri and Plasmodium ovale curtisi with Plasmodium malariae and with other Plasmodium species. International journal for parasitology. 2016;46(11):685-96.
2. Antinori S, Galimberti L, Milazzo L, Corbellino M. Plasmodium knowlesi: the emerging zoonotic malaria parasite. Acta tropica. 2013;125(2):191-201.
3. Snounou G, Sharp PM, Culleton R. The two parasite species formerly known as Plasmodium ovale. Trends in parasitology. 2024;40(1):21-7.
4. Bhatt S, Weiss D, Cameron E, Bisanzio D, Mappin B, Dalrymple U, et al. The effect of malaria control on Plasmodium falciparum in Africa between 2000 and 2015. Nature. 2015;526(7572):207-11.
5. Sinka ME, Bangs MJ, Manguin S, Rubio-Palis Y, Chareonviriyaphap T, Coetzee M, et al. A global map of dominant malaria vectors. Parasites & vectors. 2012;5(1):1-11.
6. Weiss DJ, Lucas TC, Nguyen M, Nandi AK, Bisanzio D, Battle KE, et al. Mapping the global prevalence, incidence, and mortality of Plasmodium falciparum, 2000–17: a spatial and temporal modelling study. The Lancet. 2019;394(10195):322-31.
7. Battle KE, Lucas TC, Nguyen M, Howes RE, Nandi AK, Twohig KA, et al. Mapping the global endemicity and clinical burden of Plasmodium vivax, 2000–17: a spatial and temporal modelling study. The Lancet. 2019;394(10195):332-43.
8. Price RN, Commons RJ, Battle KE, Thriemer K, Mendis K. Plasmodium vivax in the Era of the Shrinking P. falciparum Map. Trends in parasitology. 2020;36(6):560-70.
9. Mahittikorn A, Masangkay FR, Kotepui KU, Milanez GDJ, Kotepui M. Comparison of Plasmodium ovale curtisi and Plasmodium ovale wallikeri infections by a meta-analysis approach. Scientific reports. 2021;11(1):1-15.
10. Sutherland CJ. Persistent parasitism: the adaptive biology of malariae and ovale malaria. Trends in Parasitology. 2016;32(10):808-19.
11. Diagne A, Sambe BS, Gaba FM, Sarr I, Diatta AS, Sadio O, et al. Variable effects of non-falciparum species infections on malaria disease severity in high transmission regions in Senegal. Tropical Medicine and Health. 2024;52(1):93.
12. Sendor R, Banek K, Kashamuka MM, Mvuama N, Bala JA, Nkalani M, et al. Epidemiology of Plasmodium malariae and Plasmodium ovale spp. in Kinshasa Province, Democratic Republic of Congo. Nature communications. 2023;14(1):6618.
13. Omondi P, Musyoka B, Okai T, Kongere J, Kagaya W, Chan CW, et al. Non-random distribution of Plasmodium Species infections and associated clinical features in children in the lake Victoria region, Kenya, 2012-2018. Trop Med Health. 2024;52(1):52.
14. Zarocostas J. Malaria treatment should begin with parasitological diagnosis where possible, says WHO. BMJ: British Medical Journal (Online). 2010;340.
15. Newman RD. World Malaria Report 2011. 2012.
16. Musuva A, Ejersa W, Kiptui R, Memusi D, Abwao E. The malaria testing and treatment landscape in Kenya: results from a nationally representative survey among the public and private sector in 2016. Malaria journal. 2017;16(1):1-13.
17. Kamal-Yanni MM, Potet J, Saunders PM. Scaling-up malaria treatment: a review of the performance of different providers. Malaria journal. 2012;11(1):1-10.

18. Alonso P, Noor AM. The global fight against malaria is at crossroads. The Lancet. 2017;390(10112):2532-4.
19. Larsen DA, Ngwenya-Kangombe T, Cheelo S, Hamainza B, Miller J, Winters A, et al. Location, location, location: environmental factors better predict malaria-positive individuals during reactive case detection than index case demographics in Southern Province, Zambia. Malaria journal. 2017;16(1):1-9.
20. Hamainza B, Moonga H, Sikaala CH, Kamuliwo M, Bennett A, Eisele TP, et al. Monitoring, characterization and control of chronic, symptomatic malaria infections in rural Zambia through monthly household visits by paid community health workers. Malaria journal. 2014;13(1):1-19.
21. Chen I, Clarke SE, Gosling R, Hamainza B, Killeen G, Magill A, et al. "Asymptomatic" malaria: a chronic and debilitating infection that should be treated. PLoS medicine. 2016;13(1):e1001942.
22. Mueller I, Slutsker L, Tanner M. Estimating the burden of malaria: the need for improved surveillance. PLoS medicine. 2011;8(12):e1001144.
23. Kamau E, Alemayehu S, Feghali KC, Saunders D, Ockenhouse CF. Multiplex qPCR for detection and absolute quantification of malaria. PloS one. 2013;8(8):e71539.
24. Veron V, Simon S, Carme B. Multiplex real-time PCR detection of P. falciparum, P. vivax and P. malariae in human blood samples. Experimental parasitology. 2009;121(4):346-51.
25. Murray CK, Gasser Jr RA, Magill AJ, Miller RS. Update on rapid diagnostic testing for malaria. Clinical microbiology reviews. 2008;21(1):97-110.
26. Okyere B, Owusu-Ofori A, Ansong D, Buxton R, Benson S, Osei-Akoto A, et al. Point prevalence of asymptomatic Plasmodium infection and the comparison of microscopy, rapid diagnostic test and nested PCR for the diagnosis of asymptomatic malaria among children under 5 years in Ghana. PloS one. 2020;15(7):e0232874.
27. van Eijk AM, Sutton PL, Ramanathapuram L, Sullivan SA, Kanagaraj D, Priya GSL, et al. The burden of submicroscopic and asymptomatic malaria in India revealed from epidemiology studies at three varied transmission sites in India. Scientific reports. 2019;9(1):1-11.
28. Perandin F, Manca N, Calderaro A, Piccolo G, Galati L, Ricci L, et al. Development of a real-time PCR assay for detection of Plasmodium falciparum, Plasmodium vivax, and Plasmodium ovale for routine clinical diagnosis. Journal of clinical microbiology. 2004;42(3):1214-9.
29. Kamau E, Tolbert LS, Kortepeter L, Pratt M, Nyakoe N, Muringo L, et al. Development of a highly sensitive genus-specific quantitative reverse transcriptase real-time PCR assay for detection and quantitation of Plasmodium by amplifying RNA and DNA of the 18S rRNA genes. Journal of clinical microbiology. 2011;49(8):2946-53.
30. Ouédraogo AL, Eckhoff PA, Luty AJ, Roeffen W, Sauerwein RW, Bousema T, et al. Modeling the impact of Plasmodium falciparum sexual stage immunity on the composition and dynamics of the human infectious reservoir for malaria in natural settings. PLoS pathogens. 2018;14(5):e1007034.
31. Gholizadeh S, Karimi NN, Zakeri S, Djadid ND. The role of molecular techniques on malaria control and elimination programs in iran: A review article. Iranian journal of parasitology. 2018;13(2):161.
32. Tedla M. A focus on improving molecular diagnostic approaches to malaria control and elimination in low transmission settings. Parasite epidemiology and control. 2019;6:e00107.
33. Njuguna P, Maitland K, Nyaguara A, Mwanga D, Mogeni P, Mturi N, et al. Observational study: 27 years of severe malaria surveillance in Kilifi, Kenya. BMC medicine. 2019;17(1):1-9.

34. Cheaveau J, Mogollon DC, Mohon MAN, Golassa L, Yewhalaw D, Pillai DR. Asymptomatic malaria in the clinical and public health context. Expert review of anti-infective therapy. 2019;17(12):997-1010.
35. Zimmerman PA, Mehlotra RK, Kasehagen LJ, Kazura JW. Why do we need to know more about mixed Plasmodium species infections in humans? Trends in parasitology. 2004;20(9):440-7.
36. Portugal S, Drakesmith H, Mota MM. Superinfection in malaria: Plasmodium shows its iron will. EMBO reports. 2011;12(12):1233-42.
37. Imwong M, Nakeesathit S, Day NP, White NJ. A review of mixed malaria species infections in anopheline mosquitoes. Malaria journal. 2011;10(1):1-12.
38. Beier MS, Schwartz IK, Beier JC, Perkins PV, Onyango F, Koros JK, et al. Identification of malaria species by ELISA in sporozoite and oocyst infected Anopheles from western Kenya. The American journal of tropical medicine and hygiene. 1988;39(4):323-7.
39. Verra F, Angheben A, Martello E, Giorli G, Perandin F, Bisoffi Z. A systematic review of transfusion-transmitted malaria in non-endemic areas. Malaria Journal. 2018;17:1-14.
40. Orish V, Afutu L, Ayodele O, Likaj L, Marinkovic A, Sanyaolu A, editors. A 4-day incubation period of Plasmodium falciparum infection in a nonimmune patient in Ghana: a case report. Open Forum Infectious Diseases; 2019: Oxford University Press US.
41. Okafor CN, Finnigan NA. Plasmodium ovale malaria. 2018.
42. Bartoloni A, Zammarchi L. Clinical aspects of uncomplicated and severe malaria. Mediterranean journal of hematology and infectious diseases. 2012;4(1).
43. Krajden S, Panisko DM, Tobe B, Yang J, Keystone JS. Prolonged infection with Plasmodium falciparum in a semi-immune patient. Trans R Soc Trop Med Hyg. 1991;85(6):731-2.
44. Greenwood T, Vikerfors T, Sjöberg M, Skeppner G, Färnert A. Febrile Plasmodium falciparum malaria 4 years after exposure in a man with sickle cell disease. Clinical Infectious Diseases. 2008;47(4):e39-e41.
45. Szmitko PE, Kohn ML, Simor AE. Plasmodium falciparum malaria occurring 8 years after leaving an endemic area. Diagnostic microbiology and infectious disease. 2009;63(1):105-7.
46. Moonen B, Cohen JM, Snow RW, Slutsker L, Drakeley C, Smith DL, et al. Operational strategies to achieve and maintain malaria elimination. The Lancet. 2010;376(9752):1592-603.
47. Lindblade KA, Steinhardt L, Samuels A, Kachur SP, Slutsker L. The silent threat: asymptomatic parasitemia and malaria transmission. Expert review of anti-infective therapy. 2013;11(6):623-39.
48. Bottius E, Guanzirolli A, Trape J-F, Rogier C, Konate L, Druilhe P. Malaria: even more chronic in nature than previously thought; evidence for subpatent parasitaemia detectable by the polymerase chain reaction. Transactions of the Royal Society of Tropical Medicine and Hygiene. 1996;90(1):15-9.
49. Rouamba T, Samadoulougou S, Ouédraogo M, Hien H, Tinto H, Kirakoya-Samadoulougou F. Asymptomatic malaria and anaemia among pregnant women during high and low malaria transmission seasons in Burkina Faso: household-based cross-sectional surveys in Burkina Faso, 2013 and 2017. Malaria journal. 2021;20(1):1-13.
50. Abdulraheem MA, Ernest M, Ugwuanyi I, Abkallo HM, Nishikawa S, Adeleke M, et al. High prevalence of Plasmodium malariae and Plasmodium ovale in co-infections with Plasmodium falciparum in asymptomatic malaria parasite carriers in southwestern Nigeria. International journal for parasitology. 2022;52(1):23-33.
51. Nundu SS, Culleton R, Simpson SV, Arima H, Muyembe J-J, Mita T, et al. Malaria parasite species composition of Plasmodium infections among asymptomatic and symptomatic school-age

children in rural and urban areas of Kinshasa, Democratic Republic of Congo. Malaria journal. 2021;20:1-13.

52. Kho S, Qotrunnada L, Leonardo L, Andries B, Wardani PA, Fricot A, et al. Evaluation of splenic accumulation and colocalization of immature reticulocytes and Plasmodium vivax in asymptomatic malaria: A prospective human splenectomy study. PLoS medicine. 2021;18(5):e1003632.

53. Pava Z, Handayuni I, Trianty L, Utami RA, Tirta YK, Puspitasari AM, et al. Passively versus actively detected malaria: similar genetic diversity but different complexity of infection. The American journal of tropical medicine and hygiene. 2017;97(6):1788-96.

54. Buchwald AG, Sorkin JD, Sixpence A, Chimenya M, Damson M, Wilson ML, et al. Association between age and Plasmodium falciparum infection dynamics. American journal of epidemiology. 2019;188(1):169-76.

55. Worku L, Damtie D, Endris M, Getie S, Aemero M. Asymptomatic malaria and associated risk factors among school children in Sanja town, Northwest Ethiopia. International scholarly research notices. 2014;2014.

56. Kwiatkowski D, Nowak M. Periodic and chaotic host-parasite interactions in human malaria. Proceedings of the National Academy of Sciences. 1991;88(12):5111-3.

57. Mason DP, McKENZIE FE. Blood-stage dynamics and clinical implications of mixed Plasmodium vivax–Plasmodium falciparum infections. The American journal of tropical medicine and hygiene. 1999;61(3):367.

58. Richie T. Interactions between malaria parasites infecting the same vertebrate host. Parasitology. 1988;96(3):607-39.

59. Bruce MC, Day KP. Cross-species regulation of malaria parasitaemia in the human host. Current opinion in microbiology. 2002;5(4):431-7.

60. Mayxay M, Pukrittayakamee S, Newton PN, White NJ. Mixed-species malaria infections in humans. Trends in parasitology. 2004;20(5):233-40.

61. Kotepui M, Kotepui KU, Milanez GDJ, Masangkay FR. Plasmodium spp. mixed infection leading to severe malaria: A systematic review and meta-analysis. Scientific Reports. 2020;10(1):1-12.

62. Akala HM, Watson OJ, Mitei KK, Juma DW, Verity R, Ingasia LA, et al. Plasmodium interspecies interactions during a period of increasing prevalence of Plasmodium ovale in symptomatic individuals seeking treatment: an observational study. The Lancet Microbe. 2021;2(4):e141-e50.

63. Achieng AO, Muiruri P, Ingasia LA, Opot BH, Juma DW, Yeda R, et al. Temporal trends in prevalence of Plasmodium falciparum molecular markers selected for by artemether–lumefantrine treatment in pre-ACT and post-ACT parasites in western Kenya. International Journal for Parasitology: Drugs and Drug Resistance. 2015;5(3):92-9.

64. Akala HM, Eyase FL, Cheruiyot AC, Omondi AA, Ogutu BR, Waters NC, et al. Antimalarial drug sensitivity profile of western Kenya Plasmodium falciparum field isolates determined by a SYBR Green I in vitro assay and molecular analysis. The American journal of tropical medicine and hygiene. 2011;85(1):34.

65. Rougemont M, Van Saanen M, Sahli R, Hinrikson HP, Bille J, Jaton K. Detection of four Plasmodium species in blood from humans by 18S rRNA gene subunit-based and species-specific real-time PCR assays. Journal of clinical microbiology. 2004;42(12):5636-43.

66. Kamau E, Alemayehu S, Feghali KC, Juma DW, Blackstone GM, Marion WR, et al. Sample-ready multiplex qPCR assay for detection of malaria. Malaria journal. 2014;13(1):1-8.

67. Calderaro A, Piccolo G, Gorrini C, Montecchini S, Rossi S, Medici MC, et al. A new real-time PCR for the detection of Plasmodium ovale wallikeri. PloS one. 2012;7(10):e48033.
68. Hawadak J, Nana RRD, Singh V. Global trend of Plasmodium malariae and Plasmodium ovale spp. malaria infections in the last two decades (2000–2020): a systematic review and meta-analysis. Parasites & vectors. 2021;14(1):1-14.
69. Chen J, Ma X, Tang J, Xu S, Gu Y, Tang F, et al. Disparate selection of mutations in the dihydrofolate reductase gene (dhfr) of Plasmodium ovale curtisi and P. o. wallikeri in Africa. PLOS Neglected Tropical Diseases. 2022;16(12):e0010977.
70. Joste V, Coppée R, Bailly J, Rakotoarivony Y, Tchokoteu FGT, Achache S, et al. Plasmodium ovale spp dhfr mutations associated with reduced susceptibility to pyrimethamine in sub-Saharan Africa: a retrospective genetic epidemiology and functional study. The Lancet Microbe. 2024;5(7):669-78.
71. Bruce MC, Donnelly CA, Alpers MP, Galinski MR, Barnwell JW, Walliker D, et al. Cross-species interactions between malaria parasites in humans. Science. 2000;287(5454):845-8.
72. Bruce MC, Day KP. Cross-species regulation of Plasmodium parasitemia in semi-immune children from Papua New Guinea. Trends in parasitology. 2003;19(6):271-7.
73. Reece SE, Thompson J. Transformation of the rodent malaria parasite Plasmodium chabaudi and generation of a stable fluorescent line PcGFP CON. Malaria journal. 2008;7(1):1-6.
74. Mideo N. Parasite adaptations to within-host competition. Trends Parasitol. 2009;25(6):261-8.
75. Costa G, Gildenhard M, Eldering M, Lindquist RL, Hauser AE, Sauerwein R, et al. Non-competitive resource exploitation within mosquito shapes within-host malaria infectivity and virulence. Nat Commun. 2018;9(1):3474.
76. Ramiro RS, Pollitt LC, Mideo N, Reece SE. Facilitation through altered resource availability in a mixed-species rodent malaria infection. Ecology letters. 2016;19(9):1041-50.
77. Faye FB, Spiegel A, Tall A, Sokhna C, Fontenille D, Rogier C, et al. Diagnostic criteria and risk factors for Plasmodium ovale malaria. The Journal of infectious diseases. 2002;186(5):690-5.
78. Hemmer CJ, Holst FGE, Kern P, Chiwakata CB, Dietrich M, Reisinger EC. Stronger host response per parasitized erythrocyte in Plasmodium vivax or ovale than in Plasmodium falciparum malaria. Tropical Medicine & International Health. 2006;11(6):817-23.
79. McQueen PG, McKenzie FE. Competition for red blood cells can enhance Plasmodium vivax parasitemia in mixed-species malaria infections. The American journal of tropical medicine and hygiene. 2006;75(1):112.
80. Okafor CN, Finnigan NA. Plasmodium ovale malaria. StatPearls [Internet]: StatPearls Publishing; 2020.
81. Nyarko PB, Claessens A. Understanding host–pathogen–vector interactions with chronic asymptomatic malaria infections. Trends in Parasitology. 2020.

Supporting information

S1. Table 1: Primers and probes used for screening and identification of *Plasmodium* species

S2. Table 2: Metadata among symptomatic and asymptomatic *Plasmodium* ~~malaria~~ infections

Formatted: Font: Italic

S3. Table 3: Overall *Plasmodium* species frequency.

S4. Fig 1: Map showing Kombewa region where Kombewa Sub County hospital is located.

S5. Fig 2: *Plasmodium* species frequency among symptomatic infections by molecular diagnosis: Symptomatic means infections that generate malarial parasitemia alongside symptoms.

S6. Fig 3: *Plasmodium* species frequency among asymptomatic infections by molecular diagnosis: Asymptomatic *Plasmodium*~~malaria~~ parasite carriers are the infections that generate malarial parasitemia in the absence of fever or other classical symptoms and signs, in otherwise healthy individuals.

Formatted: Font: Italic

S7. Fig 4: Odds of species composition in *Plasmodium* infection presentation

Manuscript Review: **High prevalence of *Plasmodium ovalewallikeri* in co-infections with *Plasmodium falciparum* in symptomatic than asymptomatic malaria parasite carriers in Kisumu, Kenya**

We wish to convey our appreciation for the critical review and insightful comments from the reviewer. We have addressed all the queries that have made the manuscript better with more clarity and focus. Please see below the responses to the comments:

- The study is fairly well executed and presents results of original research EXCEPT these results have not been explicitly reflected and hence requires some major revision to improve the statistical meaning and clarity.
  Response: Descriptive statistics were employed to illustrate the burden of *Plasmodium* species among the symptomatic and asymptomatic groups with positive infections. For inferential statistics, proportions were compared using the Chi-square test as appropriate for the categorical data including the stratified age bands, gender and *Plasmodium* species proportions. Continuous variables, including age in years, were first assessed for normality using the Shapiro–Wilk test and then expressed as median together with their corresponding interquartile ranges (IQR) when non-normal distributions were observed. Wilcoxon rank-sum test was used for non-parametric continuous variables specifically for age in years. The geometric mean was used as a measure of central tendency for parasite density. Logistic regression analysis was also conducted to assess the varying odds of *Plasmodium* species composition. All analyses were performed using R Studio version 4.5.1. Statistical analyses were performed at the 5% significance level and the corresponding 95% confidence interval (CI). The critical significance level was set at $p \leq 0.05$.
  A detailed description on data management and statistical analysis is indicated on the manuscript line 208-222 and the results section has been revised to include the inferential and descriptive statistical analyses used.

- The results depict malaria co-infections as detected by molecular analysis which is robust but the presentation of the results does not explicitly reflect this which is misleading.
  Response: The molecular analysis results for *Plasmodium* species that were obtained following use of qPCR are presented as percentage frequencies in table 3 and in figures 2-3.
  In addition, the results section highlights the finding of the molecular analysis using qPCR which is mentioned on line 247-283. …" On this basis, *Plasmodium* species detection by qPCR assay showed that, 655/741 (88.3%) of the infections contained *P. falciparum*, 153/741 (20.7%) *P. ovalewallikeri*, 70/741 (9.4%) *P. malariae*, and 64/741 (8.7%) *P. ovalecurtisi* without consideration of the number of species per infection (Table 3). However, 12/741 (1.6%) amplifying for presence of *Plasmodium* genus did not amplify for any species…" The proportions of the *Plasmodium* species composition

following qPCR detection assays have been presented as percentage frequencies in the results and discussion sections.

- No information has been provided on the statistical analysis used to analyze the data. I have highlighted the discrepancies in the reviewed copy of the manuscript as well. For instance, this presentation "Broadly, 741 mostly contained *Pf* 668 (90·15%) followed by *Pow* 154 (20·78%), *Pm* 7041 (9·45%) and *Poc* 64 (8·7%)''. There is need to explicitly indicate that this was a co-infection analysis, without which the information is misleading as the proportions look like mere percentages of 741.
  Response: As stated above descriptive and inferential statistics were employed to illustrate the burden of *Plasmodium* species among the participants with positive infections as indicated in the manuscript, lines 215-222. Furthermore, we have indicated that *Plasmodium* co-infection analyses were conducted to detect the presence of single or multiple species and the raw data presented as percentage frequencies, lines 241-263.

- The wording reflecting the percentages and totals are not explicit. For instance, "The frequency of *Plasmodium* species among the symptomatic and asymptomatic infections were Pf 421 (96·3%) and 247 (81·25%), Pow 123 (28·3%) and 31 (10·19%), Poc 40 (9·2%) and 26 (8·55%) while Pm was 30(6·8%) and 40 (13·2%), respectively." These frequencies are depicted without clearly reflecting the denominators used, which is misleading.
  Response: We have noted this and placed the denominators/ fractions that were used to obtain each frequency in the abstract lines 45-48 and results section lines 247-283. The proportions are now OK and there is no mismatch in the numbers.

- In line 199 "*Plasmodium* speciation by qPCR assay showed that, of the 741 infections 668 (90·15%), contained *P. falciparum,* 154 (20·78%) *P. ovalewallikeri,* 70 (9·45%), *P. malariae* and 64 (8·7%) *P. ovalecurtisi*" is very confusing to readers. Without explicitly stating that co-infections were included, the percentages exceeding 100 are very misleading to readers.
  Response: We have revised the statement in the main text, results section and clarified that "*Plasmodium* co-infections analyses were conducted...", "Therefore, computing the raw frequencies of any detection of *Plasmodium* species yielded varying percentages that cumulatively exceeded 100%, consistent with the expected classification of infections containing multiple species under two or more species profiles…" Please see manuscript lines 241-246.

- In line 201-204, the counts of single species reflected are less than the sum of 544 provided.

  Response: We have rectified this and all the numbers captured in this section for the single species are reflected accurately and reads as follows: "Subsequent analysis with a focus on the presence or absence of more than one species per infection revealed that a total of 535/741 (72.2%) samples had single species while 194/741 (26.1%) had multiple species

infections. *P. falciparum* was the most frequent single-species infection occurring in 470/535 (87.9%) infections, followed *by P. ovalewallikeri* in 34/535 (6.4%), *P. malariae* in 18/535 (3.4%), and *P. ovalecurtisi* in 13/535 (2.4%) …" Please see lines 252-258 in the manuscript.

- In general, the statistics and other analyses have not been performed to a high technical standard and are not described in sufficient detail, which can be very confusing to readers, limiting the potential of replicating similar studies in the future.

  Response: We have revised the manuscript and described in detail the statistical tests used for data analysis in the data management and statistical analysis section in lines 208-222.

- The discussion and conclusions provided are very repetitive/redundant and do not clearly distinguish the alignment to the data presented in the document and the alignment towards policy recommendations.
  Response: We have revised the discussion and conclusion section to omit repetitive and redundant statements. The data has been well discussed and aligned towards policy recommendations, lines 412-419.

- The document requires stringent language revision to correct some grammar errors and syntax. Some sentences are way too long. For instance, in line 268-272 "Studies on inter-parasite interactions within human hosts suggest that parasites often interact with unrelated competitors within the host (74) and use this information to prioritize the resource allocation framework opting to increase replication to occupy most of the red blood cells at the expense of the development of sexual stages as one of the exclusion strategies". The term "use this information" in relation to parasites is misleading and should be replaced with a scientifically acceptable term.
  Response: We have revised the manuscript and corrected all the grammatical errors and syntax. We have replaced the word "information" with "mechanism" in line 328. However, some of the long sentences could not be shortened further as it would lose meaning.

- In line 106, there is a full stop before the citations.
  Response: We have corrected this and the full stop before the citation has been removed.

- Line 330 "Finally, host immunity was not characterized were not the endpoint of this study" is grammatically very poor. Other grammatical and syntax errors have been pointed/highlighted in the copy of the reviewed manuscript.
  Response: We have rectified this statement to read as follows: "Finally, host immunity was not characterized in this study because it was not one of the endpoints for the study…" Please see line 409-410.

- The research was conducted as per the international ethical standards. And the data availability guidelines have been adhered to.
  Response: We have revised the manuscript and included a statement on data availability after the main text, line 436-438.

  Closing statement.
  Many thanks for the comments and queries, we hope the manuscript is well structured.
