# Supplementary material for: High prevalence of Plasmodium ovalewallikeri in co-infections with Plasmodium falciparum in symptomatic than asymptomatic Plasmodium parasite infections in Kisumu, Kenya
Source: PLoS One. 2026 Oct 7;21(10):e0334188. doi: 10.1371/journal.pone.0334188 (PMC13645224; doi:10.1371/journal.pone.0334188)
Supplement: Supplementary file 3 [file pone.0334188.s001.docx]

Manuscript Review: High prevalence of Plasmodium ovalewallikeri in co-infections with Plasmodium falciparum in symptomatic than asymptomatic malaria parasite carriers in Kisumu, Kenya

I wish to appreciate the offer of the opportunity to review the manuscript bearing the aforestated title. The study details interesting findings in the pathogenesis of malaria with great implications for public health. Having reviewed the manuscript, I wish to submit the following remarks:

- The study is fairly well executed and presents results of original research EXCEPT these results have not been explicitly reflected and hence requires some major revision to improve the statistical meaning and clarity.
- The results depict malaria co-infections as detected by molecular analysis which is robust but the presentation of the results does not explicitly reflect this which is misleading.
- No information has been provided on the statistical analysis used to analyze the data. I have highlighted the discrepancies in the reviewed copy of the manuscript as well. For instance, this presentation “Broadly, 741 mostly contained *Pf* 668 (90·15%) followed by *Pow* 154 (20·78%), *Pm* 7041 (9·45%) and *Poc* 64 (8·7%)’’. There is need to explicitly indicate that this was a co-infection analysis, without which the information is misleading as they proportions look like mere percentages of 741.
- The wording reflecting the percentages and totals are not explicit. For instance, “The frequency of *Plasmodium* species among the symptomatic and asymptomatic infections were Pf 421 (96·3%) and 247 (81·25%), Pow 123 (28·3%) and 31 (10·19%), Poc 40 (9·2%) and 26 (8·55%) while Pm was 30(6·8%) and 40 (13·2%), respectively.” These frequencies are depicted without clearly reflecting the denominators used which is misleading.
- In line 199 “*Plasmodium* speciation by qPCR assay showed that, of the 741 infections 668 (90·15%), contained *P. falciparum,* 154 (20·78%) *P. ovalewallikeri,* 70 (9·45%), *P. malariae* and 64 (8·7%) *P. ovalecurtisi*” is very confusing to readers. Without explicitly stating that co-infections were included, the percentages exceeding 100 are very misleading to readers.
- In line 201-204, the counts of single species reflected are less than the sum of 544 provided.
- In general, the statistics, and other analyses have not been performed to a high technical standard and are not described in sufficient detail which can be very confusing to readers limiting the potential of replicating similar studies in the future.
- The discussion and conclusions provided are very repetitive/redundant and do not clearly distinguish the alignment to the data presented in the document and the alignment towards policy recommendations.
- The document requires strigent language revision to correct some errors of grammar and syntax. Some sentences are way too long. For instance, in line 268-272 “Studies on inter-parasite interactions within human hosts suggest that parasites often interact with unrelated competitors within the host (74) and use this information to prioritize the resource allocation framework opting to increase replication to occupy most of the red blood cells at the expense of the development of sexual stages as one of the exclusion strategies”. The term “use this information” in relation to parasites is misleading and should be replaced with a scientifically acceptable term.
- In line 106, there is a full stop before the citations.
- Line 330 “Finally, host immunity was not characterized were not the endpoint of this study” is grammatically very poor. Other grammatical and syntax errors have been pointed/highlighted in the copy of the reviewed manuscript.
- The research was conducted as per the international ethical standards. And the data availability guidelines have been adhered to.
