# Supplementary material for: High prevalence of Plasmodium ovalewallikeri in co-infections with Plasmodium falciparum in symptomatic than asymptomatic Plasmodium parasite infections in Kisumu, Kenya
Source: PLoS One. 2026 Oct 7;21(10):e0334188. doi: 10.1371/journal.pone.0334188 (PMC13645224; doi:10.1371/journal.pone.0334188)
Supplement: Supplementary file 4 [file pone.0334188.s003.docx]

Manuscript Review: High prevalence of *Plasmodium ovalewallikeri* in co-infections with *Plasmodium falciparum* in symptomatic than asymptomatic malaria parasite carriers in Kisumu, Kenya

We wish to convey our appreciation for the critical review and insightful comments from the reviewer. We have addressed all the queries that have made the manuscript better with more clarity and focus. Please see below the responses to the comments:

- The study is fairly well executed and presents results of original research EXCEPT these results have not been explicitly reflected and hence requires some major revision to improve the statistical meaning and clarity.

Response: Descriptive statistics were employed to illustrate the burden of *Plasmodium* species among the symptomatic and asymptomatic groups with positive infections. For inferential statistics, proportions were compared using the Chi-square test as appropriate for the categorical data including the stratified age bands, gender and *Plasmodium* species proportions. Continuous variables, including age in years, were first assessed for normality using the Shapiro–Wilk test and then expressed as median together with their corresponding interquartile ranges (IQR) when non-normal distributions were observed. Wilcoxon rank-sum test was used for non-parametric continuous variables specifically for age in years. The geometric mean was used as a measure of central tendency for parasite density. Logistic regression analysis was also conducted to assess the varying odds of *Plasmodium* species composition. All analyses were performed using R Studio version 4.5.1. Statistical analyses were performed at the 5% significance level and the corresponding 95% confidence interval (CI). The critical significance level was set at p≤0.05.

A detailed description on data management and statistical analysis is indicated on the manuscript line 208-222 and the results section has been revised to include the inferential and descriptive statistical analyses used.

- The results depict malaria co-infections as detected by molecular analysis which is robust but the presentation of the results does not explicitly reflect this which is misleading.

Response: The molecular analysis results for *Plasmodium* species that were obtained following use of qPCR are presented as percentage frequencies in table 3 and in figures 2-3.

In addition, the results section highlights the finding of the molecular analysis using qPCR which is mentioned on line 247-283. …” On this basis, *Plasmodium* species detection by qPCR assay showed that, 655/741 (88.3%) of the infections contained *P*. *falciparum*, 153/741 (20.7%) *P. ovalewallikeri*, 70/741 (9.4%) *P. malariae*, and 64/741 (8.7%) *P. ovalecurtisi* without consideration of the number of species per infection (Table 3). However, 12/741 (1.6%) amplifying for presence of *Plasmodium* genus did not amplify for any species…” The proportions of the *Plasmodium* species composition following qPCR detection assays have been presented as percentage frequencies in the results and discussion sections.

- No information has been provided on the statistical analysis used to analyze the data. I have highlighted the discrepancies in the reviewed copy of the manuscript as well. For instance, this presentation “Broadly, 741 mostly contained *Pf* 668 (90·15%) followed by *Pow* 154 (20·78%), *Pm* 7041 (9·45%) and *Poc* 64 (8·7%)’’. There is need to explicitly indicate that this was a co-infection analysis, without which the information is misleading as the proportions look like mere percentages of 741.

Response: As stated above descriptive and inferential statistics were employed to illustrate the burden of *Plasmodium* species among the participants with positive infections as indicated in the manuscript, lines 215-222. Furthermore, we have indicated that *Plasmodium* co-infection analyses were conducted to detect the presence of single or multiple species and the raw data presented as percentage frequencies, lines 241-263.

- The wording reflecting the percentages and totals are not explicit. For instance, “The frequency of *Plasmodium* species among the symptomatic and asymptomatic infections were Pf 421 (96·3%) and 247 (81·25%), Pow 123 (28·3%) and 31 (10·19%), Poc 40 (9·2%) and 26 (8·55%) while Pm was 30(6·8%) and 40 (13·2%), respectively.” These frequencies are depicted without clearly reflecting the denominators used, which is misleading.

Response: We have noted this and placed the denominators/ fractions that were used to obtain each frequency in the abstract lines 45-48 and results section lines 247-283. The proportions are now OK and there is no mismatch in the numbers.

- In line 199 “*Plasmodium* speciation by qPCR assay showed that, of the 741 infections 668 (90·15%), contained *P. falciparum,* 154 (20·78%) *P. ovalewallikeri,* 70 (9·45%), *P. malariae* and 64 (8·7%) *P. ovalecurtisi*” is very confusing to readers. Without explicitly stating that co-infections were included, the percentages exceeding 100 are very misleading to readers.

Response: We have revised the statement in the main text, results section and clarified that “*Plasmodium* co-infections analyses were conducted...”, “Therefore, computing the raw frequencies of any detection of *Plasmodium* species yielded varying percentages that cumulatively exceeded 100%, consistent with the expected classification of infections containing multiple species under two or more species profiles…” Please see manuscript lines 241-246.

- In line 201-204, the counts of single species reflected are less than the sum of 544 provided.

Response: We have rectified this and all the numbers captured in this section for the single species are reflected accurately and reads as follows: “Subsequent analysis with a focus on the presence or absence of more than one species per infection revealed that a total of 535/741 (72.2%) samples had single species while 194/741 (26.1%) had multiple species infections. *P. falciparum* was the most frequent single-species infection occurring in 470/535 (87.9%) infections, followed *by P. ovalewallikeri* in 34/535 (6.4%), *P. malariae* in 18/535 (3.4%), and *P. ovalecurtisi* in 13/535 (2.4%) …” Please see lines 252-258 in the manuscript.

- In general, the statistics and other analyses have not been performed to a high technical standard and are not described in sufficient detail, which can be very confusing to readers, limiting the potential of replicating similar studies in the future.

Response: We have revised the manuscript and described in detail the statistical tests used for data analysis in the data management and statistical analysis section in lines 208-222.

- The discussion and conclusions provided are very repetitive/redundant and do not clearly distinguish the alignment to the data presented in the document and the alignment towards policy recommendations.

Response: We have revised the discussion and conclusion section to omit repetitive and redundant statements. The data has been well discussed and aligned towards policy recommendations, lines 412-419.

- The document requires stringent language revision to correct some grammar errors and syntax. Some sentences are way too long. For instance, in line 268-272 “Studies on inter-parasite interactions within human hosts suggest that parasites often interact with unrelated competitors within the host (74) and use this information to prioritize the resource allocation framework opting to increase replication to occupy most of the red blood cells at the expense of the development of sexual stages as one of the exclusion strategies”. The term “use this information” in relation to parasites is misleading and should be replaced with a scientifically acceptable term.

Response: We have revised the manuscript and corrected all the grammatical errors and syntax. We have replaced the word “information” with “mechanism” in line 328. However, some of the long sentences could not be shortened further as it would lose meaning.

- In line 106, there is a full stop before the citations.

Response: We have corrected this and the full stop before the citation has been removed.

- Line 330 “Finally, host immunity was not characterized were not the endpoint of this study” is grammatically very poor. Other grammatical and syntax errors have been pointed/highlighted in the copy of the reviewed manuscript.

Response: We have rectified this statement to read as follows: “Finally, host immunity was not characterized in this study because it was not one of the endpoints for the study…” Please see line 409-410.

- The research was conducted as per the international ethical standards. And the data availability guidelines have been adhered to.

Response: We have revised the manuscript and included a statement on data availability after the main text, line 436-438.

Closing statement.

Many thanks for the comments and queries, we hope the manuscript is well structured.
