# Supplementary material for: High prevalence of Plasmodium ovalewallikeri in co-infections with Plasmodium falciparum in symptomatic than asymptomatic Plasmodium parasite infections in Kisumu, Kenya
Source: PLoS One. 2026 Oct 7;21(10):e0334188. doi: 10.1371/journal.pone.0334188 (PMC13645224; doi:10.1371/journal.pone.0334188)
Supplement: Supplementary file 5 [file pone.0334188.s004.docx]

Manuscript Review: High prevalence of Plasmodium ovalewallikeri in co-infections with Plasmodium falciparum in symptomatic than asymptomatic malaria parasite carriers in Kisumu, Kenya

I wish to appreciate the offer of the opportunity to re-examine the manuscript bearing the aforestated title following suggested revisions. The authors have keenly addressed the major concerns that I had raised which is highly commendable. While the science is substantially improved, I noted a few grammatical errors that need to be corrected to improve the quality of the paper. These are very minor changes as illustrated below and equally highlighted in the manuscript as tracked changes:

- In line 40 please add “participants” between symptomatic and at Kombewa for the sentence to read; 437 symptomatic participants at….
- In line 44, please add “a” between within and given for the sentence to read: within a given infection….
- In line 47, please add “samples” after the brackets with the percentages for both the first and the second percentage.
- In line 48, please add, “samples contained”, after the brackets
- In line 57, please add “the” between on and burden and “a” between suggest and need
- In line 70 please add a full-stop after the citation bracket, (6).
- In line 88, please add “a” between as and mono-infection for the sentence to read: as a mono-infection.
- In line 156, please provide the case definition for uncomplicated malaria.
- In line 252, please improve the grammar by including the words “of the samples that amplified” after the brackets with the percentage as highlighted in the manuscript.

In your results section, you have legends above and below the tables (tables 2-3), ignore if this is the required journal format.

- In line 271 please add “while” after the second bracket and “included” after three species.
- In line 283, please add “of”’ between comprised and double and “species” after double
- In line 285, add “species” after triple
- In line 323, please add “are” between and referred for the sentence to read as: and are referred to….
- In line 366, please cite the paper that has been described.
- In line 380, complete the sentence with “individuals”
- In line 394 please add “ as” between such and fever.
