# Supplementary material for: High prevalence of Plasmodium ovalewallikeri in co-infections with Plasmodium falciparum in symptomatic than asymptomatic Plasmodium parasite infections in Kisumu, Kenya
Source: PLoS One. 2026 Oct 7;21(10):e0334188. doi: 10.1371/journal.pone.0334188 (PMC13645224; doi:10.1371/journal.pone.0334188)
Supplement: Supplementary file 6 [file pone.0334188.s006.docx]

| Date: | 18th August 2026 |
| --- | --- |
| To: | The Editor |
| From: | Hoseah Akala |
| Subject: | PLOS ONE: Response to Comments PONE-D-25-45620R2 |

=============================================


PONE-D-25-45620R2
High prevalence of Plasmodium ovalewallikeri in co-infections with Plasmodium falciparum in symptomatic than asymptomatic Plasmodium parasite infections in Kisumu, Kenya

Dear Jesselle Tabamo Ababa,

Thank you for the swift response we appreciate. Please see below line-by-line response to the comments:

1. Comment: We note that there were multiple versions of Manuscript and Revised Manuscript with Track Changes and in your submission's file inventory. We have removed the older file(s), retaining the most recent version(s) for editorial review. Please double check your submission file inventory and let us know if any files appear to be outdated or absent.

Response: We have cross checked and confirmed that the Manuscript and Revised Manuscript with Track Changes versions in the submission’s file inventory contain the most recent version(s) for editorial review. All the relevant files are present including older study approval documents that had been uploaded previously for clarification following queries on the research approval documents for the studies.

2. Comment: Thank you for your detailed reply and noting there should be no supporting information for your paper. So, we wanted to double check with you regarding three files in our system that you have labeled expressly "Supporting Information":
1) WRAIR# 1384 CRR Approval_Study 1 PDF
2) WRAIR# 2064 CRR Approval_Study 2 PDF
3) Content Copyright Permission Form PDF

Please let us know if the label for these files is a mistake and they should be changed to "Other" in the file system.

Response: Concur. We realize this was an oversight on our side and have correctly labelled the three documents mentioned above as “Other” in the editorial submission system. The "Supporting Information" label for these specific documents was an error; we apologize for that.

Thank you for the key insights and thorough review of our manuscript, we hope all comments have been adequately addressed.


Kind regards,
Hoseah Akala
Corresponding Author, PI Malaria Drug Resistance Laboratory.
