# Supplementary material for: High prevalence of Plasmodium ovalewallikeri in co-infections with Plasmodium falciparum in symptomatic than asymptomatic Plasmodium parasite infections in Kisumu, Kenya
Source: PLoS One. 2026 Oct 7;21(10):e0334188. doi: 10.1371/journal.pone.0334188 (PMC13645224; doi:10.1371/journal.pone.0334188)
Supplement: Peer review history

# Peer review: Decision Letter 0

**DOI:** 10.1371/journal.pone.0334188.r001

**Authors:** Kristan Alexander Schneider

23 Jan 2026

Dear Dr. Akala,

Thank you for submitting your manuscript to PLOS ONE. After careful consideration, we feel that it has merit but does not fully meet PLOS ONE’s publication criteria as it currently stands. Therefore, we invite you to submit a revised version of the manuscript that addresses the points raised during the review process.

Please submit your revised manuscript by Mar 09 2026 11:59PM. If you will need more time than this to complete your revisions, please reply to this message or contact the journal office at plosone@plos.org. When you're ready to submit your revision, log on to https://www.editorialmanager.com/pone/ and select the 'Submissions Needing Revision' folder to locate your manuscript file.

- A letter that responds to each point raised by the academic editor and reviewer(s). You should upload this letter as a separate file labeled 'Response to Reviewers'.
- A marked-up copy of your manuscript that highlights changes made to the original version. You should upload this as a separate file labeled 'Revised Manuscript with Track Changes'.
- An unmarked version of your revised paper without tracked changes. You should upload this as a separate file labeled 'Manuscript'.

If you would like to make changes to your financial disclosure, please include your updated statement in your cover letter. Guidelines for resubmitting your figure files are available below the reviewer comments at the end of this letter.

If applicable, we recommend that you deposit your laboratory protocols in protocols.io to enhance the reproducibility of your results. Protocols.io assigns your protocol its own identifier (DOI) so that it can be cited independently in the future. For instructions see: https://journals.plos.org/plosone/s/submission-guidelines#loc-laboratory-protocols. Additionally, PLOS ONE offers an option for publishing peer-reviewed Lab Protocol articles, which describe protocols hosted on protocols.io. Read more information on sharing protocols at https://plos.org/protocols?utm_medium=editorial-email&utm_source=authorletters&utm_campaign=protocols.

We look forward to receiving your revised manuscript.

Kind regards,

Kristan Alexander Schneider, Ph.D.

Academic Editor

PLOS One

Journal Requirements:

When submitting your revision, we need you to address these additional requirements.

1. Please ensure that your manuscript meets PLOS ONE's style requirements, including those for file naming. The PLOS ONE style templates can be found at

https://journals.plos.org/plosone/s/file?id=wjVg/PLOSOne_formatting_sample_main_body.pdf and

https://journals.plos.org/plosone/s/file?id=ba62/PLOSOne_formatting_sample_title_authors_affiliations.pdf

2. We note that Figure 1 in your submission contain map images which may be copyrighted. All PLOS content is published under the Creative Commons Attribution License (CC BY 4.0), which means that the manuscript, images, and Supporting Information files will be freely available online, and any third party is permitted to access, download, copy, distribute, and use these materials in any way, even commercially, with proper attribution. For these reasons, we cannot publish previously copyrighted maps or satellite images created using proprietary data, such as Google software (Google Maps, Street View, and Earth). For more information, see our copyright guidelines: http://journals.plos.org/plosone/s/licenses-and-copyright.

We require you to either (1) present written permission from the copyright holder to publish these figures specifically under the CC BY 4.0 license, or (2) remove the figures from your submission:

1) You may seek permission from the original copyright holder of Figure 1 to publish the content specifically under the CC BY 4.0 license.

We recommend that you contact the original copyright holder with the Content Permission Form (http://journals.plos.org/plosone/s/file?id=7c09/content-permission-form.pdf) and the following text:

“I request permission for the open-access journal PLOS ONE to publish XXX under the Creative Commons Attribution License (CCAL) CC BY 4.0 (http://creativecommons.org/licenses/by/4.0/). Please be aware that this license allows unrestricted use and distribution, even commercially, by third parties. Please reply and provide explicit written permission to publish XXX under a CC BY license and complete the attached form.”

Please upload the completed Content Permission Form or other proof of granted permissions as an ""Other"" file with your submission.

In the figure caption of the copyrighted figure, please include the following text: “Reprinted from [ref] under a CC BY license, with permission from [name of publisher], original copyright [original copyright year].”

2) If you are unable to obtain permission from the original copyright holder to publish these figures under the CC BY 4.0 license or if the copyright holder’s requirements are incompatible with the CC BY 4.0 license, please either i) remove the figure or ii) supply a replacement figure that complies with the CC BY 4.0 license. Please check copyright information on all replacement figures and update the figure caption with source information. If applicable, please specify in the figure caption text when a figure is similar but not identical to the original image and is therefore for illustrative purposes only.

The following resources for replacing copyrighted map figures may be helpful:

USGS National Map Viewer (public domain): http://viewer.nationalmap.gov/viewer/

The Gateway to Astronaut Photography of Earth (public domain): http://eol.jsc.nasa.gov/sseop/clickmap/

Maps at the CIA (public domain): https://www.cia.gov/library/publications/the-world-factbook/index.html and https://www.cia.gov/library/publications/cia-maps-publications/index.html

NASA Earth Observatory (public domain): http://earthobservatory.nasa.gov/

Landsat: http://landsat.visibleearth.nasa.gov/

USGS EROS (Earth Resources Observatory and Science (EROS) Center) (public domain): http://eros.usgs.gov/#

Natural Earth (public domain): http://www.naturalearthdata.com/

3. If the reviewer comments include a recommendation to cite specific previously published works, please review and evaluate these publications to determine whether they are relevant and should be cited. There is no requirement to cite these works unless the editor has indicated otherwise.

**Additional Editor Comments:**

The reviewers found merit in the manuscript and suggested revisions. However, both reviewers raised concerns regarding the quality of the language and the statistical rigor of the manuscript.  Please keep this in mind while carefully preparing your revised manuscript. I also went over the manuscript briefly and agree with the reviewers. I also noted some text editing inconsistencies. In the abstract, sometimes a decimal point is in the normal style, e.g. 1.2, sometimes in the style of a raised decimal point, e.g. "1·2". Please use a consistent format.I also suggest to mention somewhere that *P. ovalewallikeri*  and *P. ovalecurtisi*  are now considered two morphologically identical but distinct species. Also, as far as I am concerned, the names *P. ovalewallikeri*  and *P. ovalecurtisi*  are now preferred over the classical trinominal names. At this point I would like to remind you of the PLoS One policies, which encourage authors to give detailed explanations (there is no word limit) so you can present more background information in the introduction.

[Note: HTML markup is below. Please do not edit.]

Reviewers' comments:

Reviewer's Responses to Questions

**Comments to the Author**

1. Is the manuscript technically sound, and do the data support the conclusions?

Reviewer #1: Partly

Reviewer #2: Partly

**********

2. Has the statistical analysis been performed appropriately and rigorously?

Reviewer #1: No

Reviewer #2: No

**********

3. Have the authors made all data underlying the findings in their manuscript fully available?

The PLOS Data policy

Reviewer #1: Yes

Reviewer #2: Yes

**********

4. Is the manuscript presented in an intelligible fashion and written in standard English?

Reviewer #1: No

Reviewer #2: No

**********

Reviewer #1: Comments and Recommendations

This study investigated the prevalence of co-infections with Plasmodium species among symptomatic and asymptomatic carriers of the malaria parasite in Kisumu, Kenya. It further explored the P. ovale subspecies- P. ovalewallikeri and P. ovalecurtisi in a high malaria burden area.

The authors included both symptomatic and asymptomatic individuals, allowing for direct comparison between the two study groups. Moreover, the use of qPCR enabled sensitive detection of Plasmodium species enables the detection of low-density infections, asymptomatic carriage, and mixed-species infections that microscopy would likely miss.

To enhance the strength and clarity of the manuscript, the following comments and recommendations should be addressed:

Abstract

1. The abstract should include the study design, the detection method (qPCR), and the statistical approach.

2. The authors present the comparison between species prevalence, single vs. mixed infections, symptomatic vs. asymptomatic, and specific species combinations, but fail to tell the reader the implications of each result.

3. Sort the grammatical errors: lines 33, 39, 40. Correct the repetition in the abstract: lines 47-49, 51-52, and 55-56 say essentially the same thing. Correct the style and formatting errors: Missing space: 30(6·8%), Misplaced brackets: [0.02 – 0.36, P = 0.0008) among others.

4. The logistic regression results in the abstract are different from what is displayed in Figure 4. Please reconcile. Additionally, the odds ratios reported should be correctly interpreted.

Introduction

1. The introduction needs to be more focused. The authors tell a lot about malaria before mentioning why this study was necessary.

2. The research gap appears later in the introduction section. Until lines 118-120, does the reader encounter the research gap.

3. Line 63-64- Add Plasmodium before each species name and correct the duplicate citations “(1, 2) (3)” to (1–3).

4. Line 81-85- revise the sentence to read clearly.

5. Line 103-107- please reword this sentence as WHO does not recommend the routine treatment of asymptomatic malaria infections in all settings. Instead, the WHO's general recommendation for case management is to test and treat all suspected cases with a parasitological diagnosis before administering antimalarial medicine.

6. The RDT/microscopy section can be cut without losing impact since it’s too long.

7. Correct the subject-verb agreements. For instance, Clinical symptoms of malaria starts… - start, The WHO guidelines… is based – are based, necessitating treatment seeking – treatment-seeking, etc. Also, correct the inconsistent hyphenation: non-falciparum / non falciparum.

Taken together, the introduction is too long, grammatically inconsistent, and reveals the research question later.

Methodology

1. Line 84- add period.

1. In lines 128 and 167, the headers have each word capitalized, while their sub-headers have sentence cases. Please make it consistent.

2. Line 129- the header should be in plural (Ethical considerations) as more than one approvals were obtained.

Line 130- the first sentence refers to the studies that have not been introduced prior to that sentence. The studies are introduced in the following section. Revise the first sentence, for instance, “Ethical approval was obtained from…”, without mentioning the studies not yet introduced, for a logical flow.

3. Line 136- Revise the phrase “sampling collection” to sample collection for clarity.

The header mentions study sites, but only one study site is mentioned- Kombewa Health and Demographic Surveillance System (HDSS) is in a rural part of Kisumu County. It is not clear where the other site(s) are located.

4. Line 137- the first sentence has a sentence construction error. Please rewrite for clarity.

5. Line 142- remove article “the” from the first sentence, as it implies that the studies have been mentioned before. In addition, replace running with ran or conducted to correct the inconsistent tense in the sentence. Cite the two studies at the end of the first sentence.

6. Capitalization inconsistencies- the word County in lines 138, 146, 157. The word "That" in line 144 should be in lowercase. Line 166- Sub County in the figure legend should be hyphenated for consistency.

7. Line 170- which groups are being referred to here, and where have they been introduced or defined?

8. Line 173- Make the first sentence clear.

9. Line 181- Make Genus lowercase. See line 173.

10. Line 183- Remove the space after the opening bracket.

11. Include a statistical analysis section in the methodology to describe the statistical methods used to analyze the data presented.

Results

1. Line 192- The opening bracket is repetitive and should be rewritten.

2. The sample size inconsistency in the first paragraph (total samples=741 and metadata available=526) should be clearly stated in the text.

In the opening sentence, what analysis was performed on the 741 samples, as the analysis of the metadata was performed for only a subset (526)?

3. Line 195- A statistical test of significance is required to say that a variable in the table is comparable between the two study groups. Add a third column for the p-value.

Include other clinical and laboratory variables of the study participants in the metadata table. For instance, parasitemia, vital signs that were measured (line 160),

Include the number of males and females in the study groups, i.e., n (%).

Table 2- Age is summarized using mean, median, SD, and IQR to describe distribution shape, but with age in bands, the tests within bands restrict variance. As such, the tests should be done on raw age (continuous variable), not stratified age, and then compare age between the symptomatic and asymptomatic groups using the Wilcoxon rank-sum test (Mann–Whitney U test).

Test age category distribution (bands) using χ² test of independence or Fisher’s exact (if any expected count <5). Do the same for sex (Distribution of males and females).

As a continuous variable, report median (IQR), while as bands, report n (%) in the table.

The total percentage for asymptomatic males and females does not add upto 100%.

4. Line 199- Since you have mixed infections, reporting species percentages without stating this upfront is confusing to the readers, as percentages exceed 100%. Add an exlanation.

The numbers presented in this section do not match those presented in Figure 2. Reconcile.

Line 199-204- the author should distinguish clearly between any detection (including mixed infection and single species infections only. As it's written, the reader has to infer this. 15(3·4%) missing sace, line 210.

Line 206- The opening statement is wordy and needs to be rephrased.

Line 217- the figure description in the legend does not match the figure description in Figure 2.

5. Define the symptoms of uncomplicated malaria in the methods section.

The numbers presented in this section do not match those presented in Figure 3. Reconcile.

Line 230- the figure description in the legend does not match the figure description in Figure 3.

6. Line 240-243- logistic regression does not model risk but the odds. Therefore, line 240 should say, higher odds of … instead of higher risk of …

Similarly, in line 243, the sentence should read that mixed species had reduced odds of symptomatic infection.

7. In Figure 4, please indicate in the figure legend what the colors of the error bars (blue and red)represent. Update the legend to reflect the odds and not the risk of symptomatic infection.

Discussion

1. The discussion section needs to be restructured into clear subsections: Key findings, comparison with prior studies, biological interpretation implications for transmission and elimination, and the limitations of the study, if any.

2. Some sentences are overloaded with information. For instance, paragraph 2 defines asymptomatic malaria, summarizes a cohort study, introduces parasite competition theory, and then interprets results, making it overwhelming for the reader.

3. The author repeatedly states that mixed infections differ between symptomatic and asymptomatic groups, species interaction may affect symptoms, and regional differences exist. These points should be stated clearly once, then built upon.

4. There are grammar and language problems in this section. For example, incorrect phrases include: seek treatment instead of seek for the treatment, appear to suggest instead of appear to suggests etc.

Line 259- fix the grammar in the last sentence.

Line 268- add a space after the period.

Line 292- move the citation before the period. The sentence after the citation is incomplete.

Line 330- the limitations paragraph gramartically incorrect.

5. Please reconcile the following statemets that appear as contradictions: Mixed infections are more common in symptomatic individuals [(Lines 251–252) and (Lines 275–277), and Asymptomatic individuals have more diverse species profiles (Lines 280–281). That Symptomatic group had more mixed infections and asymptomatic had greater overall species diversity is an interesting observation that needs to be explained.

6. RBC generalist/specialist argument needs to be clearly framed as a hypothesis but not as an inferred mechanism, because the study did not measure parasitemia by species, measure immune markers or model interaction effects.

7. Line 324- The definition applies to asymptomatic and not symptomatic.

8. Please discuss the logistic regression results in Figure 4

Overall, the discussion requires substantial editing for clarity, flow, and interpretation.

Reviewer #2: The study is fairly well executed and presents results of original research EXCEPT these results have not been explicitly reflected and hence requires some major revision to improve the statistical meaning and clarity. Additionally, the manuscript has grammatical errors and errors of syntax that equally need to be corrected.

**********

what does this mean?). If published, this will include your full peer review and any attached files.

If you choose “no”, your identity will remain anonymous but your review may still be made public.

**Do you want your identity to be public for this peer review?**  For information about this choice, including consent withdrawal, please see our Privacy Policy

Reviewer #1: No

Reviewer #2: No

**********

[NOTE: If reviewer comments were submitted as an attachment file, they will be attached to this email and accessible via the submission site. Please log into your account, locate the manuscript record, and check for the action link "View Attachments". If this link does not appear, there are no attachment files.]

To ensure your figures meet our technical requirements, please review our figure guidelines: https://journals.plos.org/plosone/s/figures

You may also use PLOS’s free figure tool, NAAS, to help you prepare publication quality figures: https://journals.plos.org/plosone/s/figures#loc-tools-for-figure-preparation.

NAAS will assess whether your figures meet our technical requirements by comparing each figure against our figure specifications.

Attachment Submitted filename: PLoS One Manuscript Review 19-1-2026.docx

Attachment Submitted filename: PONE-D-25-45620-reviewed.pdf

# Author comment: Author response to Decision Letter 1

**DOI:** 10.1371/journal.pone.0334188.r002

3 Jun 2026

Manuscript Review: High prevalence of Plasmodium ovalewallikeri in co-infections with Plasmodium falciparum in symptomatic than asymptomatic malaria parasite carriers in Kisumu, Kenya

We wish to convey our appreciation for the critical review and insightful comments from the reviewer. We have addressed all the queries that have made the manuscript better with more clarity and focus. Please see below the responses to the comments:

• The study is fairly well executed and presents results of original research EXCEPT these results have not been explicitly reflected and hence requires some major revision to improve the statistical meaning and clarity.

Response: Descriptive statistics were employed to illustrate the burden of Plasmodium species among the symptomatic and asymptomatic groups with positive infections. For inferential statistics, proportions were compared using the Chi-square test as appropriate for the categorical data including the stratified age bands, gender and Plasmodium species proportions. Continuous variables, including age in years, were first assessed for normality using the Shapiro–Wilk test and then expressed as median together with their corresponding interquartile ranges (IQR) when non-normal distributions were observed. Wilcoxon rank-sum test was used for non-parametric continuous variables specifically for age in years. The geometric mean was used as a measure of central tendency for parasite density. Logistic regression analysis was also conducted to assess the varying odds of Plasmodium species composition. All analyses were performed using R Studio version 4.5.1. Statistical analyses were performed at the 5% significance level and the corresponding 95% confidence interval (CI). The critical significance level was set at p≤0.05.

A detailed description on data management and statistical analysis is indicated on the manuscript line 208-222 and the results section has been revised to include the inferential and descriptive statistical analyses used.

• The results depict malaria co-infections as detected by molecular analysis which is robust but the presentation of the results does not explicitly reflect this which is misleading.

Response: The molecular analysis results for Plasmodium species that were obtained following use of qPCR are presented as percentage frequencies in table 3 and in figures 2-3.

In addition, the results section highlights the finding of the molecular analysis using qPCR which is mentioned on line 247-283. …” On this basis, Plasmodium species detection by qPCR assay showed that, 655/741 (88.3%) of the infections contained P. falciparum, 153/741 (20.7%) P. ovalewallikeri, 70/741 (9.4%) P. malariae, and 64/741 (8.7%) P. ovalecurtisi without consideration of the number of species per infection (Table 3). However, 12/741 (1.6%) amplifying for presence of Plasmodium genus did not amplify for any species…” The proportions of the Plasmodium species composition following qPCR detection assays have been presented as percentage frequencies in the results and discussion sections.

• No information has been provided on the statistical analysis used to analyze the data. I have highlighted the discrepancies in the reviewed copy of the manuscript as well. For instance, this presentation “Broadly, 741 mostly contained Pf 668 (90·15%) followed by Pow 154 (20·78%), Pm 7041 (9·45%) and Poc 64 (8·7%)’’. There is need to explicitly indicate that this was a co-infection analysis, without which the information is misleading as the proportions look like mere percentages of 741.

Response: As stated above descriptive and inferential statistics were employed to illustrate the burden of Plasmodium species among the participants with positive infections as indicated in the manuscript, lines 215-222. Furthermore, we have indicated that Plasmodium co-infection analyses were conducted to detect the presence of single or multiple species and the raw data presented as percentage frequencies, lines 241-263.

• The wording reflecting the percentages and totals are not explicit. For instance, “The frequency of Plasmodium species among the symptomatic and asymptomatic infections were Pf 421 (96·3%) and 247 (81·25%), Pow 123 (28·3%) and 31 (10·19%), Poc 40 (9·2%) and 26 (8·55%) while Pm was 30(6·8%) and 40 (13·2%), respectively.” These frequencies are depicted without clearly reflecting the denominators used, which is misleading.

Response: We have noted this and placed the denominators/ fractions that were used to obtain each frequency in the abstract lines 45-48 and results section lines 247-283. The proportions are now OK and there is no mismatch in the numbers.

• In line 199 “Plasmodium speciation by qPCR assay showed that, of the 741 infections 668 (90·15%), contained P. falciparum, 154 (20·78%) P. ovalewallikeri, 70 (9·45%), P. malariae and 64 (8·7%) P. ovalecurtisi” is very confusing to readers. Without explicitly stating that co-infections were included, the percentages exceeding 100 are very misleading to readers.

Response: We have revised the statement in the main text, results section and clarified that “Plasmodium co-infections analyses were conducted...”, “Therefore, computing the raw frequencies of any detection of Plasmodium species yielded varying percentages that cumulatively exceeded 100%, consistent with the expected classification of infections containing multiple species under two or more species profiles…” Please see manuscript lines 241-246.

• In line 201-204, the counts of single species reflected are less than the sum of 544 provided.

Response: We have rectified this and all the numbers captured in this section for the single species are reflected accurately and reads as follows: “Subsequent analysis with a focus on the presence or absence of more than one species per infection revealed that a total of 535/741 (72.2%) samples had single species while 194/741 (26.1%) had multiple species infections. P. falciparum was the most frequent single-species infection occurring in 470/535 (87.9%) infections, followed by P. ovalewallikeri in 34/535 (6.4%), P. malariae in 18/535 (3.4%), and P. ovalecurtisi in 13/535 (2.4%) …” Please see lines 252-258 in the manuscript.

• In general, the statistics and other analyses have not been performed to a high technical standard and are not described in sufficient detail, which can be very confusing to readers, limiting the potential of replicating similar studies in the future.

Response: We have revised the manuscript and described in detail the statistical tests used for data analysis in the data management and statistical analysis section in lines 208-222.

• The discussion and conclusions provided are very repetitive/redundant and do not clearly distinguish the alignment to the data presented in the document and the alignment towards policy recommendations.

Response: We have revised the discussion and conclusion section to omit repetitive and redundant statements. The data has been well discussed and aligned towards policy recommendations, lines 412-419.

• The document requires stringent language revision to correct some grammar errors and syntax. Some sentences are way too long. For instance, in line 268-272 “Studies on inter-parasite interactions within human hosts suggest that parasites often interact with unrelated competitors within the host (74) and use this information to prioritize the resource allocation framework opting to increase replication to occupy most of the red blood cells at the expense of the development of sexual stages as one of the exclusion strategies”. The term “use this information” in relation to parasites is misleading and should be replaced with a scientifically acceptable term.

Response: We have revised the manuscript and corrected all the grammatical errors and syntax. We have replaced the word “information” with “mechanism” in line 328. However, some of the long sentences could not be shortened further as it would lose meaning.

• In line 106, there is a full stop before the citations.

Response: We have corrected this and the full stop before the citation has been removed.

• Line 330 “Finally, host immunity was not characterized were not the endpoint of this study” is grammatically very poor. Other grammatical and syntax errors have been pointed/highlighted in the copy of the reviewed manuscript.

Response: We have rectified this statement to read as follows: “Finally, host immunity was not characterized in this study because it was not one of the endpoints for the study…” Please see line 409-410.

• The research was conducted as per the international ethical standards. And the data availability guidelines have been adhered to.

Response: We have revised the manuscript and included a statement on data availability after the main text, line 436-438.

Closing statement.

Many thanks for the comments and queries, we hope the manuscript is well structured.

Attachment Submitted filename: Response to Reviewers_11May2026.docx

# Peer review: Decision Letter 1

**DOI:** 10.1371/journal.pone.0334188.r003

**Authors:** Kristan Alexander Schneider

16 Jul 2026

Dear Dr. Akala,

Thank you for submitting your manuscript to PLOS ONE. After careful consideration, we feel that it has merit but does not fully meet PLOS ONE’s publication criteria as it currently stands. Therefore, we invite you to submit a revised version of the manuscript that addresses the points raised during the review process.

Please submit your revised manuscript by Aug 30 2026 11:59PM. If you will need more time than this to complete your revisions, please reply to this message or contact the journal office at plosone@plos.org. When you're ready to submit your revision, log on to https://www.editorialmanager.com/pone/ and select the 'Submissions Needing Revision' folder to locate your manuscript file.

- A letter that responds to each point raised by the academic editor and reviewer(s). You should upload this letter as a separate file labeled 'Response to Reviewers'.
- A marked-up copy of your manuscript that highlights changes made to the original version. You should upload this as a separate file labeled 'Revised Manuscript with Track Changes'.
- An unmarked version of your revised paper without tracked changes. You should upload this as a separate file labeled 'Manuscript'.

If you would like to make changes to your financial disclosure, please include your updated statement in your cover letter. Guidelines for resubmitting your figure files are available below the reviewer comments at the end of this letter.

If applicable, we recommend that you deposit your laboratory protocols in protocols.io to enhance the reproducibility of your results. Protocols.io assigns your protocol its own identifier (DOI) so that it can be cited independently in the future. For instructions see: https://journals.plos.org/plosone/s/submission-guidelines#loc-laboratory-protocols. Additionally, PLOS ONE offers an option for publishing peer-reviewed Lab Protocol articles, which describe protocols hosted on protocols.io. Read more information on sharing protocols at https://plos.org/protocols?utm_medium=editorial-email&utm_source=authorletters&utm_campaign=protocols.

As the corresponding author, your ORCID iD is verified in the submission system and will appear in the published article. PLOS supports the use of ORCID, and we encourage all coauthors to register for an ORCID iD and use it as well. Please encourage your coauthors to verify their ORCID iD within the submission system before final acceptance, as unverified ORCID iDs will not appear in the published article. *Only*  the individual author can complete the verification step; PLOS staff *cannot*  verify ORCID iDs on behalf of authors.

We look forward to receiving your revised manuscript.

Kind regards,

Kristan Alexander Schneider, Ph.D.

Academic Editor

PLOS One

Journal Requirements:

If the reviewer comments include a recommendation to cite specific previously published works, please review and evaluate these publications to determine whether they are relevant and should be cited. There is no requirement to cite these works unless the editor has indicated otherwise.

Please review your reference list to ensure that it is complete and correct. If you have cited papers that have been retracted, please include the rationale for doing so in the manuscript text, or remove these references and replace them with relevant current references. Any changes to the reference list should be mentioned in the rebuttal letter that accompanies your revised manuscript. If you need to cite a retracted article, indicate the article’s retracted status in the References list and also include a citation and full reference for the retraction notice.

Additional Editor Comments:

We are converging. The reviewers were very positive. Reviewer #2 listed a number of minor changes. Please incorporate them inn your revised version.

[Note: HTML markup is below. Please do not edit.]

Reviewers' comments:

Reviewer's Responses to Questions

**Comments to the Author**

Reviewer #1: All comments have been addressed

Reviewer #2: All comments have been addressed

**********

2. Is the manuscript technically sound, and do the data support the conclusions?

Reviewer #1: Yes

Reviewer #2: Yes

**********

3. Has the statistical analysis been performed appropriately and rigorously?

Reviewer #1: Yes

Reviewer #2: Yes

**********

4. Have the authors made all data underlying the findings in their manuscript fully available?

The PLOS Data policy

Reviewer #1: Yes

Reviewer #2: Yes

**********

5. Is the manuscript presented in an intelligible fashion and written in standard English?

Reviewer #1: Yes

Reviewer #2: No

**********

Reviewer #1: (No Response)

Reviewer #2: The authors have keenly addressed the major concerns that I had raised which is highly commendable. While the science is substantially improved, I noted a few grammatical errors that need to be corrected to improve the quality of the paper. These are very minor changes as illustrated below and equally highlighted in the manuscript as tracked changes:

� In line 40 please add “participants” between symptomatic and at Kombewa for the sentence to read; 437 symptomatic participants at….

� In line 44, please add “a” between within and given for the sentence to read: within a given infection….

� In line 47, please add “samples” after the brackets with the percentages for both the first and the second percentage.

� In line 48, please add, “samples contained”, after the brackets

� In line 57, please add “the” between on and burden and “a” between suggest and need

� In line 70 please add a full-stop after the citation bracket, (6).

� In line 88, please add “a” between as and mono-infection for the sentence to read: as a mono-infection.

� In line 156, please provide the case definition for uncomplicated malaria.

� In line 252, please improve the grammar by including the words “of the samples that amplified” after the brackets with the percentage as highlighted in the manuscript.

In your results section, you have legends above and below the tables (tables 2-3), ignore if this is the required journal format.

� In line 271 please add “while” after the second bracket and “included” after three species.

� In line 283, please add “of”’ between comprised and double and “species” after double

� In line 285, add “species” after triple

� In line 323, please add “are” between and referred for the sentence to read as: and are referred to….

� In line 366, please cite the paper that has been described.

� In line 380, complete the sentence with “individuals”

� In line 394 please add “ as” between such and fever.

**********

what does this mean?). If published, this will include your full peer review and any attached files.

If you choose “no”, your identity will remain anonymous but your review may still be made public.

**Do you want your identity to be public for this peer review?**  For information about this choice, including consent withdrawal, please see our Privacy Policy

Reviewer #1: No

Reviewer #2: No

**********

[NOTE: If reviewer comments were submitted as an attachment file, they will be attached to this email and accessible via the submission site. Please log into your account, locate the manuscript record, and check for the action link "View Attachments". If this link does not appear, there are no attachment files.]

To ensure your figures meet our technical requirements, please review our figure guidelines: https://journals.plos.org/plosone/s/figures

You may also use PLOS’s free figure tool, NAAS, to help you prepare publication quality figures: https://journals.plos.org/plosone/s/figures#loc-tools-for-figure-preparation.

NAAS will assess whether your figures meet our technical requirements by comparing each figure against our figure specifications.

Attachment Submitted filename: Manuscript Review comments 29-6-2026.docx

Attachment Submitted filename: PONE-D-25-45620_R1 29-6-2026.pdf

# Author comment: Author response to Decision Letter 2

**DOI:** 10.1371/journal.pone.0334188.r004

18 Aug 2026

1. If the authors have adequately addressed your comments raised in a previous round of review and you feel that this manuscript is now acceptable for publication, you may indicate that here to bypass the “Comments to the Author” section, enter your conflict-of-interest statement in the “Confidential to Editor” section, and submit your "Accept" recommendation.

Reviewer #1: All comments have been addressed

Reviewer #2: All comments have been addressed

Response: Thank you for the positive feedback, we truly appreciate

________________________________________

2. Is the manuscript technically sound, and does the data support the conclusions?

The manuscript must describe a technically sound piece of scientific research with data that supports the conclusions. Experiments must have been conducted rigorously, with appropriate controls, replication, and sample sizes. The conclusions must be drawn appropriately based on the data presented.

Reviewer #1: Yes

Reviewer #2: Yes

Response: Many thanks for the comments, we are grateful.

________________________________________

3. Has the statistical analysis been performed appropriately and rigorously?

Reviewer #1: Yes

Reviewer #2: Yes

Response: This is well noted.

________________________________________

4. Have the authors made all data underlying the findings in their manuscript fully available?

The PLOS Data policy requires authors to make all data underlying the findings described in their manuscript fully available without restriction, with rare exception (please refer to the Data Availability Statement in the manuscript PDF file). The data should be provided as part of the manuscript or its supporting information or deposited to a public repository. For example, in addition to summary statistics, the data points behind means, medians and variance measures should be available. If there are restrictions on publicly sharing data—e.g. participant privacy or use of data from a third party—those must be specified.

Reviewer #1: Yes

Reviewer #2: Yes

Response: Noted.

________________________________________

5. Is the manuscript presented in an intelligible fashion and written in standard English?

PLOS ONE does not copyedit accepted manuscripts, so the language in submitted articles must be clear, correct, and unambiguous. Any typographical or grammatical errors should be corrected at revision, so please note any specific errors here.

Reviewer #1: Yes

Reviewer #2: No

Response: The manuscript has been revised and formatted according to the reviewers’ comments. The current manuscript reads well.

________________________________________

6. Review Comments to the Author

Please use the space provided to explain your answers to the questions above. You may also include additional comments for the author, including concerns about dual publication, research ethics, or publication ethics. (Please upload your review as an attachment if it exceeds 20,000 characters)

Reviewer #1: (No Response)

Reviewer #2: The authors have keenly addressed the major concerns that I had raised which is highly commendable. While the science is substantially improved, I noted a few grammatical errors that need to be corrected to improve the quality of the paper. These are very minor changes as illustrated below and equally highlighted in the manuscript as tracked changes:

� In line 40 please add “participants” between symptomatic and at Kombewa for the sentence to read; 437 symptomatic participants at….

Response: Done

� In line 44, please add “a” between within and given for the sentence to read: within a given infection….

Response: Done.

� In line 47, please add “samples” after the brackets with the percentages for both the first and the second percentage.

Response: Done.

� In line 48, please add, “samples contained”, after the brackets

Response: Done.

� In line 57, please add “the” between on and burden and “a” between suggest and need

Response: Done.

� In line 70 please add a full-stop after the citation bracket, (6).

Response: Done.

� In line 88, please add “a” between as and mono-infection for the sentence to read: as a mono-infection.

Response: Done.

� In line 156, please provide the case definition for uncomplicated malaria.

Response: Thank you for the comment. An appropriate definition of uncomplicated malaria has been added to the draft. The section reads, “… having symptoms consistent with uncomplicated malaria, characterized by symptomatic, laboratory-confirmed Plasmodium infection in a patient who had no clinical or laboratory evidence of severe malaria or vital organ dysfunction and able to tolerate oral antimalarial therapy. These individuals presented with clinical features including fever (≥37.5 °C) or a history of fever within the preceding 48 hours accompanied by symptoms such as Chills, rigors, sweating, Headache, Malaise, fatigue Myalgia and arthralgia, Nausea and/or vomiting.”

� In line 252, please improve the grammar by including the words “of the samples that amplified” after the brackets with the percentage as highlighted in the manuscript.

Response: Done

In your results section, you have legends above and below the tables (tables 2-3), ignore if this is the required journal format.

Response: This is part of the journal format hence no changes.

� In line 271 please add “while” after the second bracket and “included” after three species.

� In line 283, please add “of”’ between comprised and double and “species” after double

� In line 285, add “species” after triple

� In line 323, please add “are” between and referred for the sentence to read as: and are referred to….

Response for lines 271,283, 285, 323: Done, the grammatical errors in the manuscript have been corrected.

� In line 366, please cite the paper that has been described.

Response: Concur. This was an omission. Citations, “(Hemmer et al., 2006, Hemmer et al., 1991)” have been inserted

� In line 380, complete the sentence with “individuals”

� In line 394 please add “as” between such and fever.

Response for lines 380 and 394: Done

________________________________________

Closing Statement; Thank you for your kind consideration and valuable inputs to make the current manuscript even better. We look forward to continuing fruitful engagements in the future.

Attachment Submitted filename: Response to Reviewers_18August2026.docx

# Editor report: Decision Letter 2

**DOI:** 10.1371/journal.pone.0334188.r005

**Authors:** Kristan Alexander Schneider

11 Sep 2026

High prevalence of Plasmodium ovalewallikeri in co-infections with Plasmodium falciparum in symptomatic than asymptomatic Plasmodium parasite infections in Kisumu, Kenya

PONE-D-25-45620R2

Dear Dr. Akala,

We’re pleased to inform you that your manuscript has been judged scientifically suitable for publication and will be formally accepted for publication once it meets all outstanding technical requirements.

Within one week, you’ll receive an e-mail detailing the required amendments. When these have been addressed, you’ll receive a formal acceptance letter and your manuscript will be scheduled for publication.

An invoice will be generated when your article is formally accepted. Please note, if your institution has a publishing partnership with PLOS and your article meets the relevant criteria, all or part of your publication costs will be covered. Please make sure your user information is up-to-date by logging into Editorial Manager at Editorial Manager® and clicking the ‘Update My Information' link at the top of the page. For questions related to billing, please contact billing support.

If your institution or institutions have a press office, please notify them about your upcoming paper to help maximize its impact. If they’ll be preparing press materials, please inform our press team as soon as possible -- no later than 48 hours after receiving the formal acceptance. Your manuscript will remain under strict press embargo until 2 pm Eastern Time on the date of publication. For more information, please contact onepress@plos.org.

Kind regards,

Kristan Alexander Schneider, Ph.D.

Academic Editor

PLOS One

Additional Editor Comments (optional): I noticed there are some inconsistencies in the typesetting. E.g. "Fig.1 Map of Kombewa" at line 178 should be "Fig 1 …". Similarly, “Fig. 2” occurs several times in the text and it should be “Fig 2”. Please carefully check the typesetting for consistency and make sure you follow the journal's guidelines.

Reviewers' comments:

# Editor report: Acceptance letter

**DOI:** 10.1371/journal.pone.0334188.r006

**Authors:** Kristan Alexander Schneider

PONE-D-25-45620R2

PLOS One

Dear Dr. Akala,

I'm pleased to inform you that your manuscript has been deemed suitable for publication in PLOS One. Congratulations! Your manuscript is now being handed over to our production team.

At this stage, our production department will prepare your paper for publication. This includes ensuring the following:

* All references, tables, and figures are properly cited

* All relevant supporting information is included in the manuscript submission,

* There are no issues that prevent the paper from being properly typeset

You will receive further instructions from the production team, including instructions on how to review your proof when it is ready. Please keep in mind that we are working through a large volume of accepted articles, so please give us a few days to review your paper and let you know the next and final steps.

Lastly, if your institution or institutions have a press office, please let them know about your upcoming paper now to help maximize its impact. If they'll be preparing press materials, please inform our press team within the next 48 hours. Your manuscript will remain under strict press embargo until 2 pm Eastern Time on the date of publication. For more information, please contact onepress@plos.org.

You will receive an invoice from PLOS for your publication fee after your manuscript has reached the completed accept phase. If you receive an email requesting payment before acceptance or for any other service, this may be a phishing scheme. Learn how to identify phishing emails and protect your accounts at https://explore.plos.org/phishing.

If we can help with anything else, please email us at customercare@plos.org.

Thank you for submitting your work to PLOS One and supporting open access.

Kind regards,

PLOS One Editorial Office Staff

on behalf of

Professor Kristan Alexander Schneider

Academic Editor

PLOS One
